# Implementation of deep learning-based auto-segmentation for radiotherapy planning structures: a workflow study at two cancer centers

**DOI:** 10.1186/s13014-021-01831-4

**Published:** 2021-06-08

**Authors:** Jordan Wong, Vicky Huang, Derek Wells, Joshua Giambattista, Jonathan Giambattista, Carter Kolbeck, Karl Otto, Elantholi P. Saibishkumar, Abraham Alexander

**Affiliations:** 1 BC Cancer – Vancouver, 600 W 10th Ave, Rm 4550, Vancouver, BC V5Z 4E6 Canada; 2 BC Cancer – Fraser Valley, 13750 96th Avenue, Surrey, BC V3V 1Z2 Canada; 3 BC Cancer – Victoria, 2410 Lee Avenue, Victoria, BC V8R 6V5 Canada; 4 Saskatchewan Cancer Agency, 503-1801 Hamilton St, Regina, SK S4P 4B4 Canada; 5 Limbus AI Inc, 2076 Athol Street, Regina, SK S4T 3E5 Canada

**Keywords:** Machine learning, Radiotherapy, Radiotherapy planning, Computer-assisted

## Abstract

**Purpose:**

We recently described the validation of deep learning-based auto-segmented contour (DC) models for organs at risk (OAR) and clinical target volumes (CTV). In this study, we evaluate the performance of implemented DC models in the clinical radiotherapy (RT) planning workflow and report on user experience.

**Methods and materials:**

DC models were implemented at two cancer centers and used to generate OAR and CTVs for all patients undergoing RT for a central nervous system (CNS), head and neck (H&N), or prostate cancer. Radiation Therapists/Dosimetrists and Radiation Oncologists completed post-contouring surveys rating the degree of edits required for DCs (1 = minimal, 5 = significant) and overall DC satisfaction (1 = poor, 5 = high). Unedited DCs were compared to the edited treatment approved contours using Dice similarity coefficient (DSC) and 95% Hausdorff distance (HD).

**Results:**

Between September 19, 2019 and March 6, 2020, DCs were generated on approximately 551 eligible cases. 203 surveys were collected on 27 CNS, 54 H&N, and 93 prostate RT plans, resulting in an overall survey compliance rate of 32%. The majority of OAR DCs required minimal edits subjectively (mean editing score ≤ 2) and objectively (mean DSC and 95% HD was ≥ 0.90 and ≤ 2.0 mm). Mean OAR satisfaction score was 4.1 for CNS, 4.4 for H&N, and 4.6 for prostate structures. Overall CTV satisfaction score (n = 25), which encompassed the prostate, seminal vesicles, and neck lymph node volumes, was 4.1.

**Conclusions:**

Previously validated OAR DC models for CNS, H&N, and prostate RT planning required minimal subjective and objective edits and resulted in a positive user experience, although low survey compliance was a concern. CTV DC model evaluation was even more limited, but high user satisfaction suggests that they may have served as appropriate starting points for patient specific edits.

**Supplementary Information:**

The online version contains supplementary material available at 10.1186/s13014-021-01831-4.

## Introduction

Manual contouring of organs at risk (OAR) and clinical target volumes (CTV) is an essential task in radiotherapy (RT) planning. However, this process can be time consuming, depends on staff availability, and is a large contributor to RT treatment planning lead time. Auto-segmentation solutions are frequently explored to alleviate workload pressures [1], and deep learning-based auto-segmentation is thought to provide improved results over atlas-based methods [2]. Despite its potential utility, deep learning-based auto-segmentation is not yet widely used in clinical practice [3]. One possible factor associated with the slow adoption is the current lack of knowledge and guidelines regarding the commissioning and implementation of such machine learning applications [3].

In our previous report [4], we compared deep-learning based auto-segmented contours (DC) with multiple expert Radiation Oncologist contours for central nervous system (CNS), head and neck (H&N), and prostate OARs and CTVs and observed close similarity between the two contour sets. DCs were also noted to take substantially less time to produce compared to manual contours, although this did not take into account the amount of potential editing that may be done in clinical practice. Considering the results of our previous study, these auto-segmentation models were approved at our institutions for implementation and testing in the clinical workflow with the intention of facilitating current manual contouring processes.

In the current study, we aim to characterize the impact of these DC models in the clinical workflow at two cancer centers. Capturing DC editing time to quantify time savings was not felt to be feasible given the associated added tasks involved, so other subjective and objective measures were devised to assess DC model performance and degree of editing required. By sharing our experience implementing machine learning auto-segmentation into the workflow, we hope to increase interest in adopting machine learning auto-segmentation applications in other Radiation Oncology clinical practices.

## Methods

The Limbus Contour auto-segmentation software version 1.0.22 was implemented at two British Columbia Cancer centers. Description of the software and its DC models are described in our previous report [4] but complete details have not been made public by the manufacturer. These models were trained using publically available data; no local institutional data was used. Approval for this study was obtained from our institutional research ethics board and consent from the participating departments was obtained. Planning computed tomography (CT) images from both centers were captured using a GE Healthcare Optima CT580 series scanner with the following parameters depending on disease site: 120kVp, 100–700mAs, 1.25–2.5 mm slice thickness, and 0.683–1.270 mm in-plane pixel size. Using these CT images, the auto-segmentation software prospectively generated DCs to be reviewed and edited on all patients undergoing RT treatment planning for CNS, H&N, and prostate malignancies. The software was set up to automatically detect the planning CT image files and create DCs to be imported alongside the images into the treatment planning software.

The OARs to be auto-segmented for each eligible disease site were selected by each center; the OARs available to be selected included brainstem, globe, optic chiasm, optic nerve, parotid, submandibular, mandible, spinal cord, bladder, femoral head, and rectum. The CTV contours available were neck CTV, prostate, and seminal vesicles (SV). DCs for neck CTV included lymph node levels Ib, II, III, IV, V, and the retropharyngeal and retrostyloid nodes; this DC was generated on every third image slice as they were intended to be edited and interpolated according to the clinical scenario.

Generated DCs underwent manual review and were edited as needed prior to being used for RT treatment planning. Radiation Therapists/Dosimetrists are responsible for OAR generation at both centers and performed the majority of OAR DC editing during this study. These contours are then sent to the Radiation Oncologist for review of OARs and creation of the target volumes, including adjusting the CTV DC if present. Uncommonly, unedited OAR DCs could be reviewed and edited by a Radiation Oncologist without a Radiation Therapists/Dosimetrists assessment due to department workload and/or urgency of treatment. The two study centers are involved in resident physician teaching, but no resident physicians were at these centers during the study period and therefore no trainees were involved in reviewing and/or editing study contours.

A subjective assessment of DC editing and workflow impact was captured by having Radiation Therapists/Dosimetrists and Radiation Oncologists complete post-contouring surveys for each RT plan, rating the degree of edits performed on the DCs (1 = minimal, 5 = significant) and their overall OAR and CTV DC satisfaction (1 = poor, 5 = high) based on their own assessment; no further instructions were provided and the surveys did not record the name of the user to encourage candid feedback. A text field was also available for any free text comments. Radiation Therapists/Dosimetrists completed these surveys after their contouring was completed and before the contours were sent to the Radiation Oncologist for their review. Cases could have more than one survey entry since different OARs may be contoured by different Radiation Therapists/Dosimetrists, and Radiation Oncologists would create a separate entry for the CTV before sending the contours for peer review and treatment planning. When multiple survey entries existed for an RT plan, the entries were combined and only the worst score for each structure or satisfaction rating was kept.

Objective comparison metrics were also captured for patients with a completed survey and an approved RT treatment plan by the study end date. In this analysis, the unedited DCs were compared to the final treatment approved contours using Dice similarity coefficient (DSC) and 95% Hausdorff distance (HD) to provide an objective assessment of DC editing. Since inaccurate DCs could still provide workflow benefits compared to wholly manually generated contours, these comparison metrics are less useful than the survey assessments at characterizing workflow impact of DC implementation. However, the objective comparisons do assist in the identification of consistent DC contouring errors that can then be the target of model training and improvement.

DSC is defined as D(A,B) = 2|A ∩ B|/(|A| +|B|) and describes the relative overlap of segmentation volumes A and B. DSC values range from 0 for no overlap to 1 for complete overlap. HD is a bi-directional measure to quantify the distance between two contour surfaces. The 95% HD is the distance that represents the largest surface-to-surface separation among the closest 95% of surface points. For example, if a 95% HD is 2 mm, then 95% of contour A points are within 2 mm of contour B points. No comparisons were performed on RT plans containing DCs without a survey, as we could not verify that these DCs were utilized.

No cropping of the superior or inferior borders for these structures was performed for this analysis. Prostate volumes were compared to the closest CTV volume regardless as to whether the CTV included a portion of the SV, except for post-prostatectomy cases. No contour comparison was performed for SV, as there was no CTV volume that was felt to be appropriate for comparison; a CTV volume that excluded the prostate was only available in a handful of cases and it typically only included the proximal SV. No contour comparisons were performed for neck CTVs, since the DCs were only generated on every third slice.

Assessment of the rectum, prostate, and SV from prostate cases were excluded from the overall analysis when a rectal spacer gel was used, as this scenario was not included in model training; OAR DC models for cases with rectal spacers are currently in development.

## Results

DC models were implemented into the clinical workflow at both centers on September 19, 2019. From this date until March 6, 2020, DCs were generated for 606 RT plans (370 at center A and 236 at center B). However, this number includes an estimated 40–70 non-prostate pelvic RT cases from center A in which auto-segmentation was requested off study; a more specific number was not able to be determined.

Not all eligible cases had a survey completed for them due to compliance. 203 post-contouring surveys were collected on 174 cases (27 CNS, 54 H&N, and 93 prostate RT plans);153 (88%) of these cases were from center A, resulting in an approximate survey compliance rate of 46–51% (153/300-330) at center A and 9% (21/236) at center B.

Approximately 22 and 7 Radiation Therapists/Dosimetrists from center A and B, respectively, participated in completing the surveys, while approximately 10 Radiation Oncologists from center A and none from center B were involved in the surveys. 185 of the surveys (91%) were completed by a Radiation Therapist/Dosimetrists; 29 cases had 2 survey entries that were combined.

**Fig. 1:**
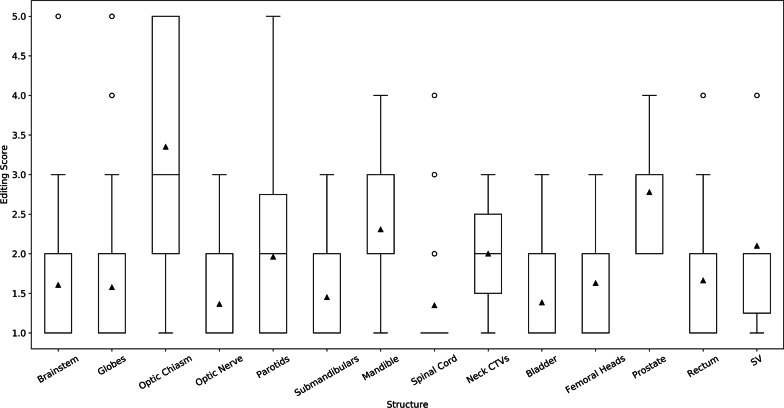
Box plots of user editing scores for central nervous system, head and neck, and prostate organs at risk and clinical target volumes (CTV). 1 = minimal editing required, 5 = significant editing required. SV = seminal vesicles

Five prostate cases had rectal spacer gel in-situ; the editing scores for the rectum, prostate, and SV from these cases were excluded from the overall analysis, but are reported separately in the Additional file 1. Other specific patient and disease characteristics were not captured as part of this study. Table 1 contains the number of entries and editing scores for each OAR and CTV structure. The editing score dataset is also represented as box plots in Fig. 1. The survey editing score data by center is summarized in the Additional file 2.

**Table 1:** Summary of editing scores for central nervous system, head and neck, and prostate organs at risk and clinical target volumes (CTV)

Structure	Number	Median (range)	Mean
Brainstem	38	1 (1–5)	1.6
Globes	66	1 (1–5)	1.6
Optic Chiasm	20	3 (1–5)	3.4
Optic Nerve	22	1 (1–3)	1.4
Spinal Cord	46	1 (1–4)	1.3
Parotids	26	2 (1–5)	2.0
Submandibulars	20	1 (1–3)	1.5
Mandible	26	2 (1–4)	2.3
Neck CTV	2	2 (1–3)	2.0
Bladder	86	1 (1–3)	1.4
Femoral Heads	89	2 (1–3)	1.6
Prostate	9	3 (2–4)	2.8
Rectum	83	1 (1–4)	1.7
Seminal Vesicles	10	2 (1–4)	2.1

1 = minimal editing required, 5 = significant editing required

**Fig. 2:**
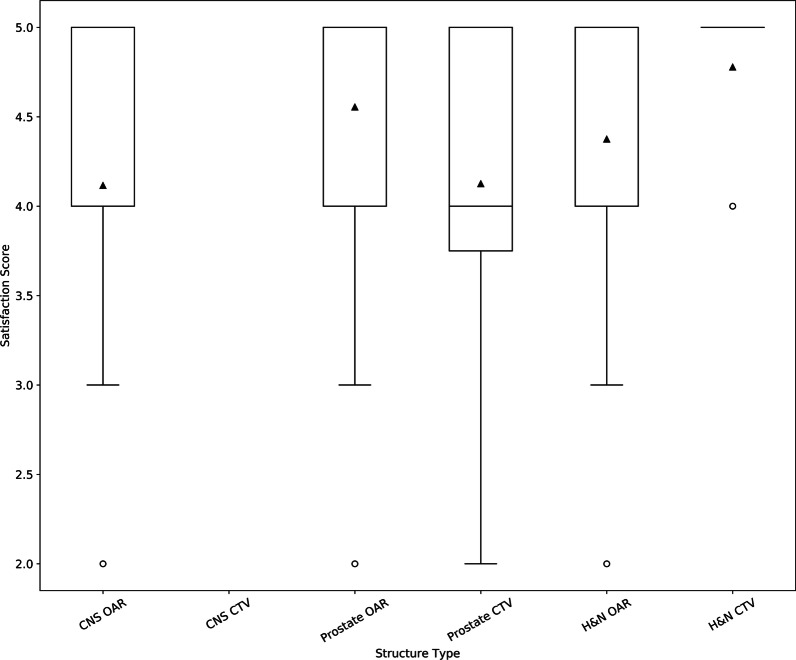
Box plot of user satisfaction scores for central nervous system (CNS), head and neck (H&N), and prostate organs at risk (OAR) and clinical target volumes (CTV). 1 = low satisfaction, 5 = high satisfaction

The submitted surveys contained 157 OAR satisfaction scores with a mean score of 4.4 (range 2–5, median 5). Of these 157 scores, 26, 48, and 83, were from CNS, H&N, and prostate RT plans, respectively, with mean satisfaction scores of 4.1, 4.4, and 4.6, respectively. OAR and CTV satisfaction scores by disease site are represented in Fig. 2. There were 25 CTV satisfaction scores with a mean score of 4.3 (range 2–5, median 5). Of these 25 satisfaction scores, 9 and 16 were from H&N and prostate RT plans, respectively, with mean satisfaction scores of 4.8 and 4.1, respectively.

During the study period, 130 of the 174 cases (75%) had approved RT treatment plans available and were included in the contour comparison analysis. The remaining 25% of cases corresponded to patients whose treatment was cancelled, patients who were re-planned with another planning CT, or RT plans that had not yet been approved by the study end date. The unedited DCs were compared to the final treatment approved contours for 23 CNS, 36 H&N, and 71 prostate RT plans. Examples of the unedited and edited DCs can be found in the Additional file 3. A total of 54 cases (42%; 21 CNS, 12 H&N, 21 prostate) had registered magnetic resonance (MR) images.

**Fig. 3:**
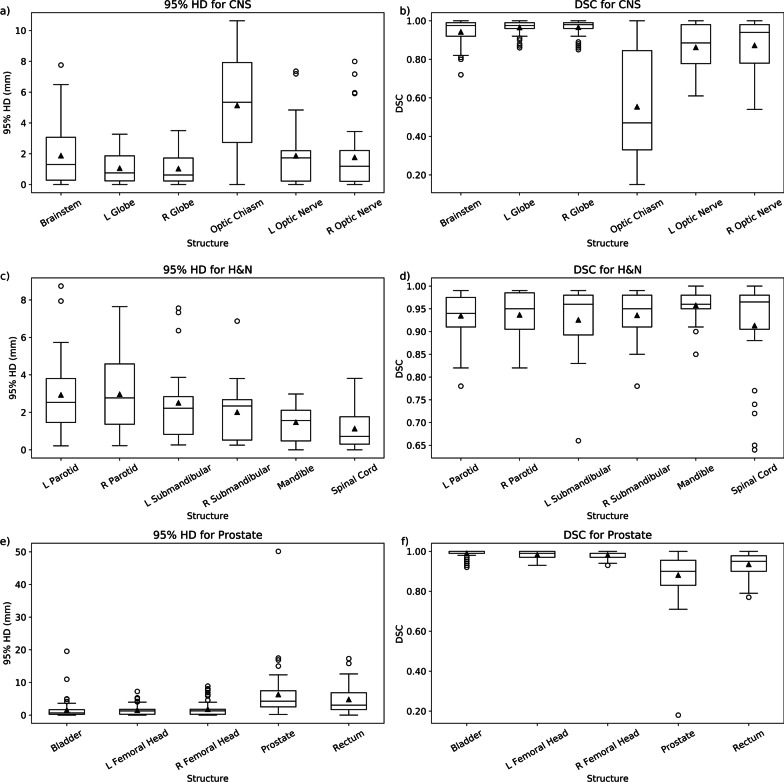
Box plots of Dice similarity coefficient (DSC) and 95% Hausdorff distance (HD) from comparing unedited deep learning-based contours with final treatment approved contours for central nervous system (CNS; **a**, **b**), head and neck (H&N; **c**, **d**), and prostate (**e**, **f**) organs at risk (OAR) and clinical target volumes (CTV). L = left, R = right

**Fig. 4:**
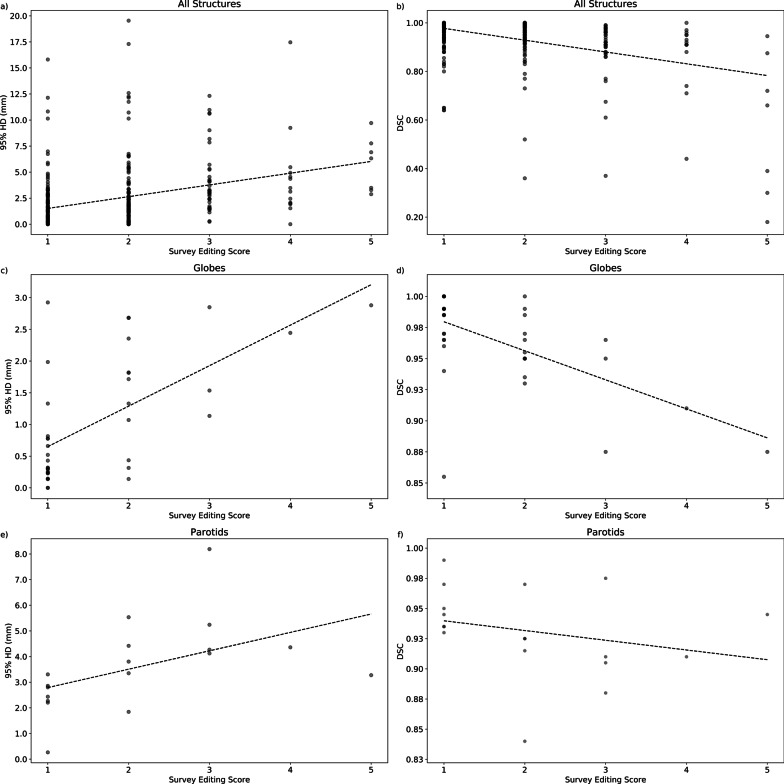
Plots correlating survey editing score with Dice similarity coefficient (DSC) and 95% Hausdorff distance (HD) from comparing unedited deep learning-based contours with final treatment approved contours for all (**a**, **b**) and select (**c**–**f**) central nervous system, head and neck, and prostate organs at risk and clinical target volumes. Best-fit line is shown on each plot

Table 2 contains a summary of the 95% HD and DSC scores and box plots are shown in Fig. 3. The summarized contour comparison data by center are also presented in the Additional file 3. Select graphs correlating the survey editing score with the 95% HD or DSC for each structure can be found in Fig. 4, with the remaining plots shared in the Additional file 2.

**Table 2:** Summary of comparison metrics from comparing unedited deep learning-based auto-segmented contours and final treatment approved contours for central nervous system, head and neck, and prostate organs at risk and clinical target volumes (CTV)

Structure	Number	95% Hausdorff distance (mm)		Dice similarity coefficient	
		Median (range)	Mean	Median (range)	Mean
Brainstem	56	1.31 (0–7.76)	1.87	0.98 (0.72–1)	0.94
Left Globe	48	0.76 (0–3.27)	1.06	0.98 (0.86–1)	0.97
Right Globe	47	0.62 (0–3.5)	1.03	0.98 (0.85–1)	0.97
Optic Chiasm	30	5.35 (0–10.63)	5.13	0.47 (0.15–1)	0.55
Left Optic Nerve	32	1.73 (0–7.36)	1.86	0.89 (0.61–1)	0.86
Right Optic Nerve	33	1.19 (0–7.99)	1.76	0.94 (0.54–1)	0.87
Spinal Cord	38	0.72 (0–3.81)	1.13	0.97 (0.64–1)	0.91
Left Parotid	35	2.53 (0.21–8.74)	2.93	0.94 (0.78–0.99)	0.93
Right Parotid	35	2.77 (0.22–7.64)	2.96	0.95 (0.82–0.99)	0.94
Left Submandibular	20	2.22 (0.26–7.56)	2.51	0.96 (0.66–0.99)	0.93
Right Submandibular	23	2.34 (0.25–6.87)	2.00	0.95 (0.78–0.99)	0.94
Mandible	34	1.56 (0–2.98)	1.47	0.96 (0.85–1)	0.96
Bladder	71	0.64 (0–19.54)	1.51	0.99 (0.92–1)	0.99
Left Femoral Head	71	1.27 (0–7.24)	1.48	0.99 (0.93–1)	0.98
Right Femoral Head	71	1.28 (0–8.88)	1.77	0.99 (0.93–1)	0.98
Prostate	51	4.26 (0.2–50.15)	6.29	0.9 (0.18–1)	0.88
Rectum	71	3.04 (0–17.3)	4.76	0.95 (0.77–1)	0.93

## Discussion

With increasing interest and uptake of machine learning applications and auto-segmentation in Radiation Oncology [3], literature to help promote and guide the commissioning and clinical implementation of these algorithms is becoming more readily available [5]. While machine learning auto-segmentation is widely hypothesized to be associated with workflow benefits and time savings, limited prospective data exists to confirm this claim. To our knowledge, only one other study characterizing the workflow impact of DC model implementation has been published to date and this report evaluated the use of prostate magnetic resonance (MR)-based DCs [6], as opposed to the CT-based models in this study that may be more widely applicable to typical Radiation Oncology practices.

We implemented previously validated DC models for CNS, H&N, and prostate RT planning and found that OAR DCs required minimal subjective editing and were associated with high user satisfaction. Objectively comparing the unedited DC with the final treatment approved contour also indicated that major edits were uncommon, including in cases with moderate artifact (e.g. example H&N case in Additional file 2). Evaluation of CTV DCs for H&N and prostate RT planning was more limited but the submitted survey satisfaction scores suggest that they may have been used favorably as a template for patient specific edits and interpolation.

Both centers in our study reported noticeable time savings with using DCs to the study team, but we unfortunately did not have a feasible method to evaluate RT plan contouring times before and after auto-segmentation implementation, which would have demonstrated workflow benefit more definitively. For this study, there was consensus within the two participating centers that having users record contouring and editing times for each RT plan was not practical. Other available metrics, such as tracking contouring task completion time, were also not considered to be reliable given that this metric could encompass staffing availability delays much larger in scale than any auto-segmentation time savings.

We therefore relied on post-contouring survey feedback as a quantifiable indicator as to whether DCs impeded, rather than streamlined, existing workflow with the presumption that any unusable DCs would result in poor editing scores and overall satisfaction results. Survey assessments were used in the previously mentioned workflow study [6] and such an evaluation approach appears consistent with published auto-segmentation implementation recommendations [5]; these recommendations acknowledge that while time savings is the rationale, evaluating the degree of manual editing required and having an avenue for feedback are also important results to capture.

To minimize possible bias and obtain as close to a real-world representation of impact as possible, no specific instructions on how to assess the auto-segmentations on the surveys were provided. On a 5 point scale (1 = minimal, 5 = significant edits required), average editing scores were 2 or less for brainstem, globe, optic nerve, parotid, submandibular, spinal cord, bladder, femoral head, and rectum DCs. The OARs with higher average editing scores (optic chiasm and mandible) are discussed separately. Overall satisfaction scores for OAR DCs from all 3 disease sites had a mean satisfaction score of > 4 (1 = low satisfaction, 5 = high user satisfaction).

These favourable survey results suggest that the OAR DCs were associated with a clinical workflow benefit and likely resulted in time savings. This finding is supported by a recent contouring study that utilized the same commercial software as the present study and demonstrated time savings with editing DCs over manual contours for the bladder and rectum [7].

However, compliance with the surveys was not ideal; survey completion rates were approximately 48% and 9% at the two centers despite multiple reminders sent during the study and some survey entries did not have assessments of all relevant structures even though those DCs appear to have been utilized. From discussion with the participating centers, the DCs were regularly used in workflow and the staff felt that the surveys were unnecessary as DCs lead to a noticeable improvement in contouring processes; however, no metrics to support this claim are available. An additional consideration is that the survey results could have been influenced by a multitude of factors related to the subjective nature of this assessment method, including differing user contouring experience and biases for or against auto-segmentation implementation.

Objective comparison metrics were also evaluated, but given that there are no clinically relevant thresholds at which DCs can be determined to be beneficial to workflow, the subjective results are likely more indicative of whether DCs were felt to facilitate RT contouring. This is supported by the aforementioned workflow study by Cha et al., which noted a 30% time savings with using prostate MR-based DCs compared to historic controls but found that their geometric comparison results did not strongly correlate with contouring time [6].

Even so, we found the comparison metrics to still be useful for identifying DC outliers with lower similarity to the final treatment approved contour and these outliers are apparent on the 95% HD and DSC box plots (Fig. 3). On review of these cases, we discovered instances of the bladder DCs including adjacent bowel or prostate, rectum DCs being inaccurate in certain cases with significant amounts of gas, and salivary gland DCs being under-contoured when there was adjacent tumor. These scenarios and inaccuracies occurred infrequently so should not preclude the implementation of DCs, but they highlight an important benefit of DC models over atlas-based models. Through ongoing user feedback and monitoring of DC performance, areas of poor performance can be identified to guide further training and improve DC accuracy, which is generally not possible with atlas-based auto-segmentation methods.

The optic chiasm (editing score 3.4) was one of the two OAR DC models with a mean survey editing score > 2. From the contour comparison analysis, the optic chiasm DC can be seen in Fig. 3 to also objectively require more user adjustments with mean 95% HD and DSC of 5.13 mm and 0.55, respectively. The increased amount of editing needed is consistent with our previous findings of this structure having a large degree of inter-observer variability in contouring [4], which likely pertains to varying contouring preferences of optic nerve and optic chiasm junctions and difficulty visualizing the optic chiasm on CT images. Furthermore, 23 of the 30 cases in this study that utilized the optic chiasm DC had MR images registered to their planning CT scan to help delineate this structure. An MR-based DC model for the optic chiasm and other CNS OARS are currently in development, but some degree of inter-observer variations will likely always be present [8].

The mandible DC model was not evaluated in our previous validation study, but was included in this study since we hypothesized that the minimal anatomic variability in this structure would lead to high performance. However, this structure was the other OAR DC that was noted to subjectively require a larger degree of manual edits with a mean editing score of 2.3. Despite this higher mean and relatively wider spread of editing scores seen in Fig. 1, the mandible was minimally edited objectively, as shown by low mean 95% HD of 1.47 mm and high mean DSC values of 0.96. Our contour comparison results were also comparable or improved to other DC and atlas based methods [9–11]. One possible explanation for the discrepancy between objective and subjective editing scores could be that small edits may have been performed on many image slices of this larger structure and felt to be manually tedious resulting in a higher editing score, but these edits may have had minimal effects on the geometric contour comparison metrics. Conversely, large and obvious inaccuracies on only a few image slices may have had a similar effect. We are unable to confirm what influenced the discrepancy without having known the rater’s thoughts at the time of editing, but ongoing evaluation of the mandible DC model performance in clinical workflow will help characterize if any specific editing is consistently needed for this structure.

CTVs for H&N and prostate were evaluated in this study, but a fewer number of survey entries for these structures were available. Radiation Therapists/Dosimetrists would defer contouring of these structures to Radiation Oncologists and compliance with the post-contouring surveys among this user group was much lower. From the small amount of data collected, CTVs subjectively seemed to require a moderate amount of editing, which was to be expected as these contours depend on the clinical scenario. The 25 submitted overall CTV satisfaction scores were moderate to high (Fig. 2), perhaps suggesting that CTV DCs likely achieved their purpose of being appropriate templates for manual patient specific edits.

No contour comparison analysis was performed for neck CTVs or SVs, since there were no appropriate approved structures to compare the DCs to. CTVs for prostate RT planning can include a portion of the proximal SV, so a direct geometric evaluation of the prostate contours was also not possible. However, we opted to still compare the unedited prostate DC to the closest matching approved CTV volume to estimate how much editing for this structure might have occurred. From this comparison, we observed a mean 95% HD of 6.29 mm and mean DSC of 0.88. These values appear to be within range of the inter-observer variability seen for the prostate in our previous validation study (average worst expert to expert 95% HD and DSC of 5.3 mm and 0.83, respectively) [4] and are similar to the prostate and SV comparison indices in the aforementioned MR-based workflow study (mean DSC 0.89) [6], so it is possible that the prostate DCs at least did not require an excessive degree of editing.

The primary goal of this study was to evaluate the workflow impact of DC implementation through subjective and objective measures. As a secondary ad hoc analysis, the values from each case were plotted together for each structure (Fig. 4 and Additional file 1) to explore the relationship between survey editing scores and comparison metrics. The slope of the best-fit lines on these graphs suggests that higher editing scores were associated with increased objective editing (i.e. higher 95% HD and lower DSC). On the other hand, Cha et al. observed in their workflow study that their geometric indices were not strongly correlated with the physician quality scores [6]. This disagreement in findings may be related to the differing subjective assessment scale used (3 point vs 5 point scale) or the method of score assignment (rating each structure vs a global rating).

On closer examination of our graphs, some plots had more shallow best-fit lines than others, potentially indicating that there was a less prominent association with editing score and comparison metrics for these structures. One hypothesis for this observation was that users may have assigned higher editing scores to small DC inaccuracies that could have significant clinical implications, while these inaccuracies would have minimal effects on the 95% HD and DSC metrics which only consider geometric information.

For example, the steep best-fit line of the optic globe (Fig. 4c, d) suggests a close association with editing scores and comparison metrics; this structure typically will have few clinically significant inaccuracies since it is usually not in close proximity to a target and the dose-constraint of concern is a maximum dose [12] which would tend to not change significantly with minor contouring differences. On the other hand, the parotid gland has a more shallow best-fit line (Fig. 4e, f) suggesting a weaker association; this structure more likely will have DC inaccuracies that are felt to be more clinically impactful since the relevant dose constraint considers the mean dose to the whole parotid volume [13].

As we touched upon earlier, the absence of a contouring and editing time metric, low survey compliance, and possible survey biases represented limitations of this study. Additionally, the limited utility of the objective comparison metrics in assessing DC model performance in the clinical workflow should be considered when interpreting those results. Other than there being no relevant threshold and their not being able to take into account any clinical information, these metrics are also susceptible to inconsistencies secondary to operational procedures. For instance, many of the spinal cord DSC outliers were from comparing spinal cord DCs with a treatment approved spinal cord structure that actually represented the spinal canal. Other comparisons were not able to be performed because the DC was used to create a differently named structure (e.g. the globe was used to create a retina structure). Moreover, falsely high similarity was possible when DCs were used but not closely reviewed, which might occur when a requested OAR DC is far away from the target volume (e.g. the optic chiasm for an oropharyngeal or larynx target).

In summary, we implemented deep-learning based auto-segmentation for CNS, H&N and prostate OARs and CTVs into the clinical RT planning workflow at two cancer centers and captured subjective and objective measures of DC performance. Our results suggest that well-trained DC models were associated with a positive user experience and did not require any degree of manual editing that would appear to inhibit their usability. As this software continues to be utilized by our centers, scenarios associated with consistent DC underperformance can be identified and targeted with additional training to further improve DC model accuracy. Additional OAR and CTV DC models, such as those applicable to breast, thoracic, and gynecological RT treatment planning, are currently being developed and tested in workflow by a variety of groups, including at our own institutions [14–17].

## Supplementary Information

**Additional file 1**. Supplementary tables.

**Additional file 2**. Additional plots correlating survey editing scores with Dice similariy coefficients and 95% Hausdorff distances.

**Additional file 3**. Examples cases with unedited and edited organs and risk and clinical target volume contours. 

## Data Availability

The datasets generated and/or analysed during the current study are not publicly available due to patient confidentiality, but may be available from the corresponding author on reasonable request.
