# Supplementary material for: Implementation of deep learning-based auto-segmentation for radiotherapy planning structures: a workflow study at two cancer centers
Source: Radiat Oncol. 2021 Jun 8;16:101. doi: 10.1186/s13014-021-01831-4 (PMC8186196; doi:10.1186/s13014-021-01831-4)

## Slide 1
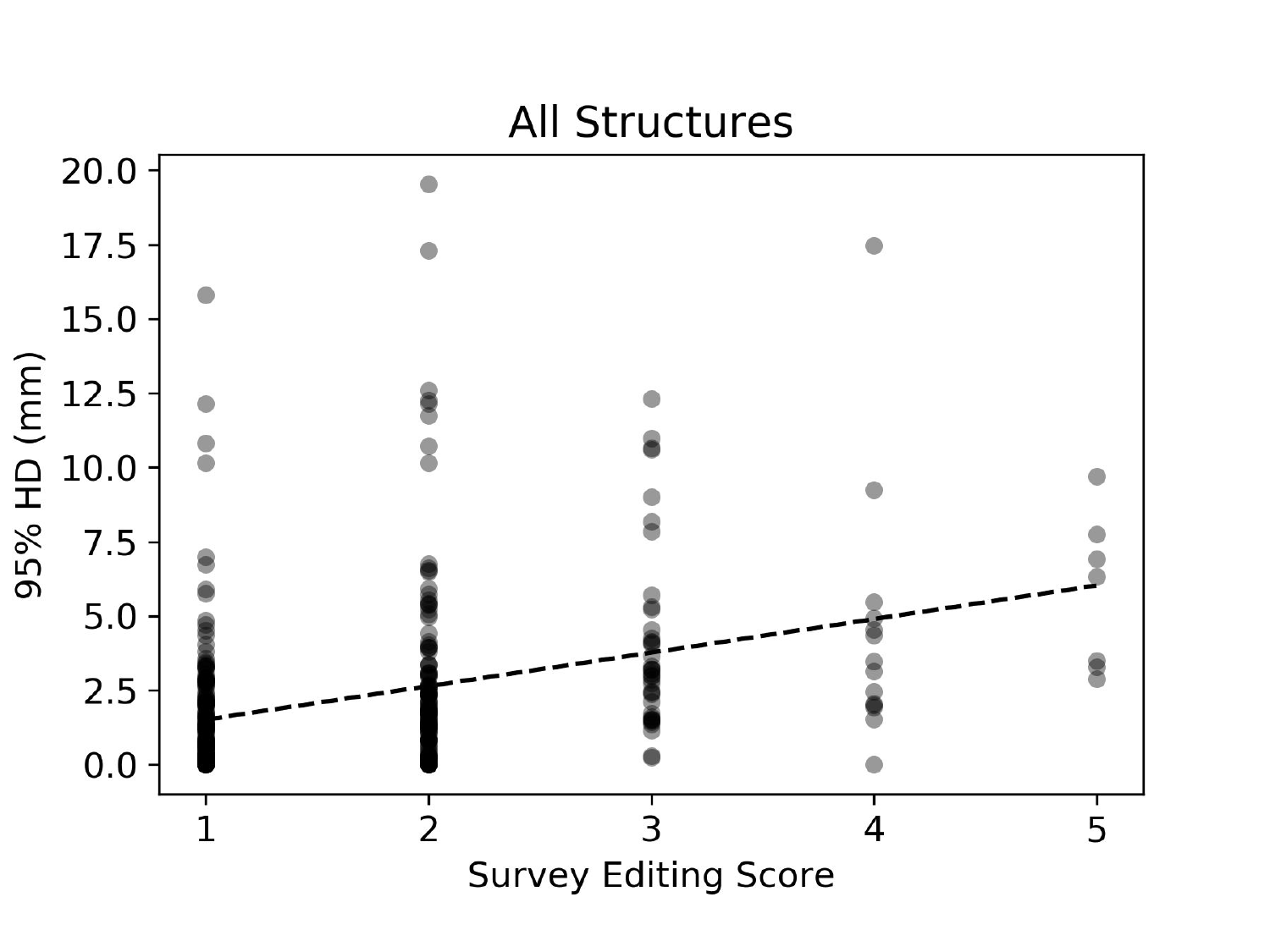

## Slide 2
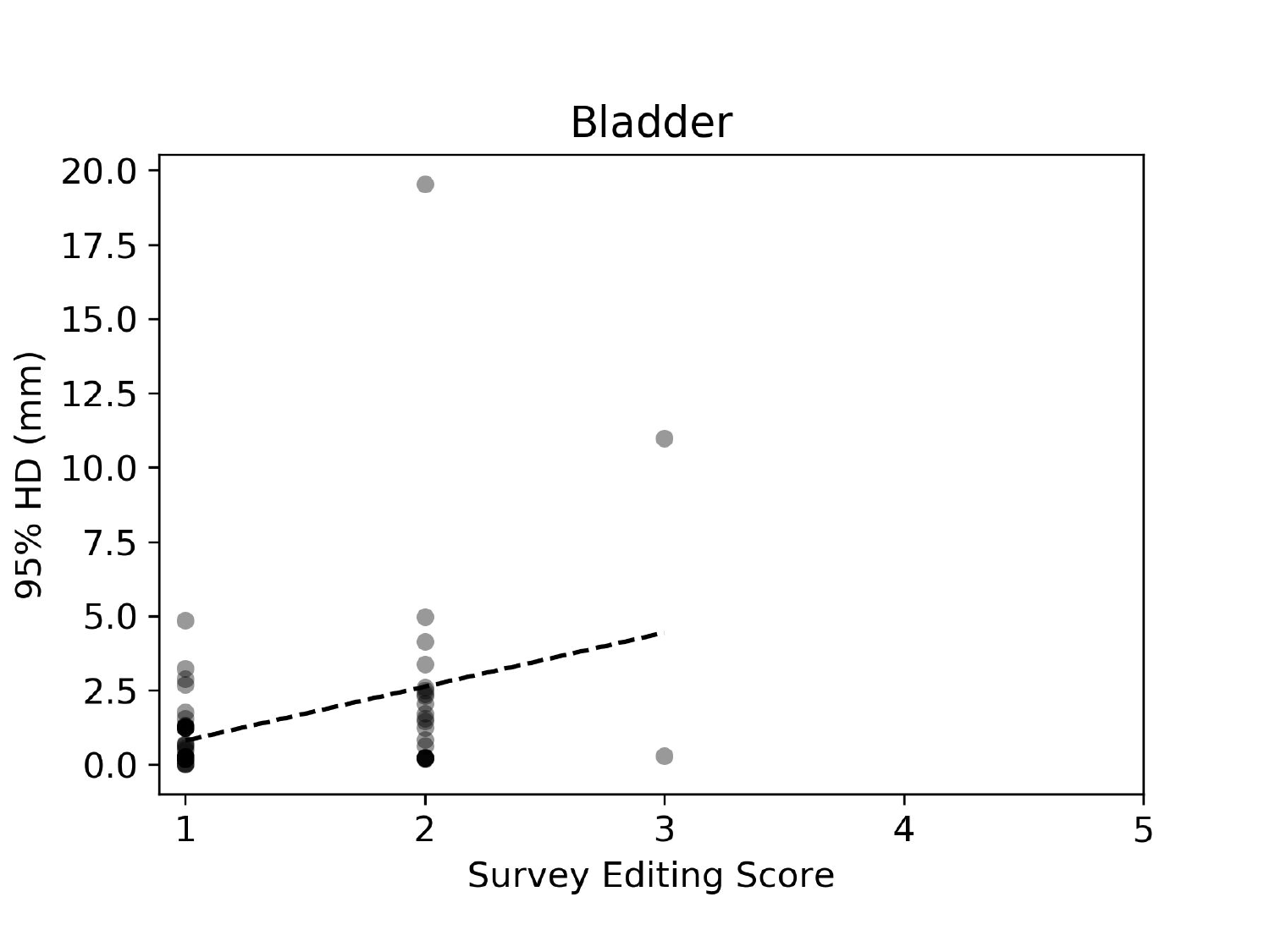

## Slide 3
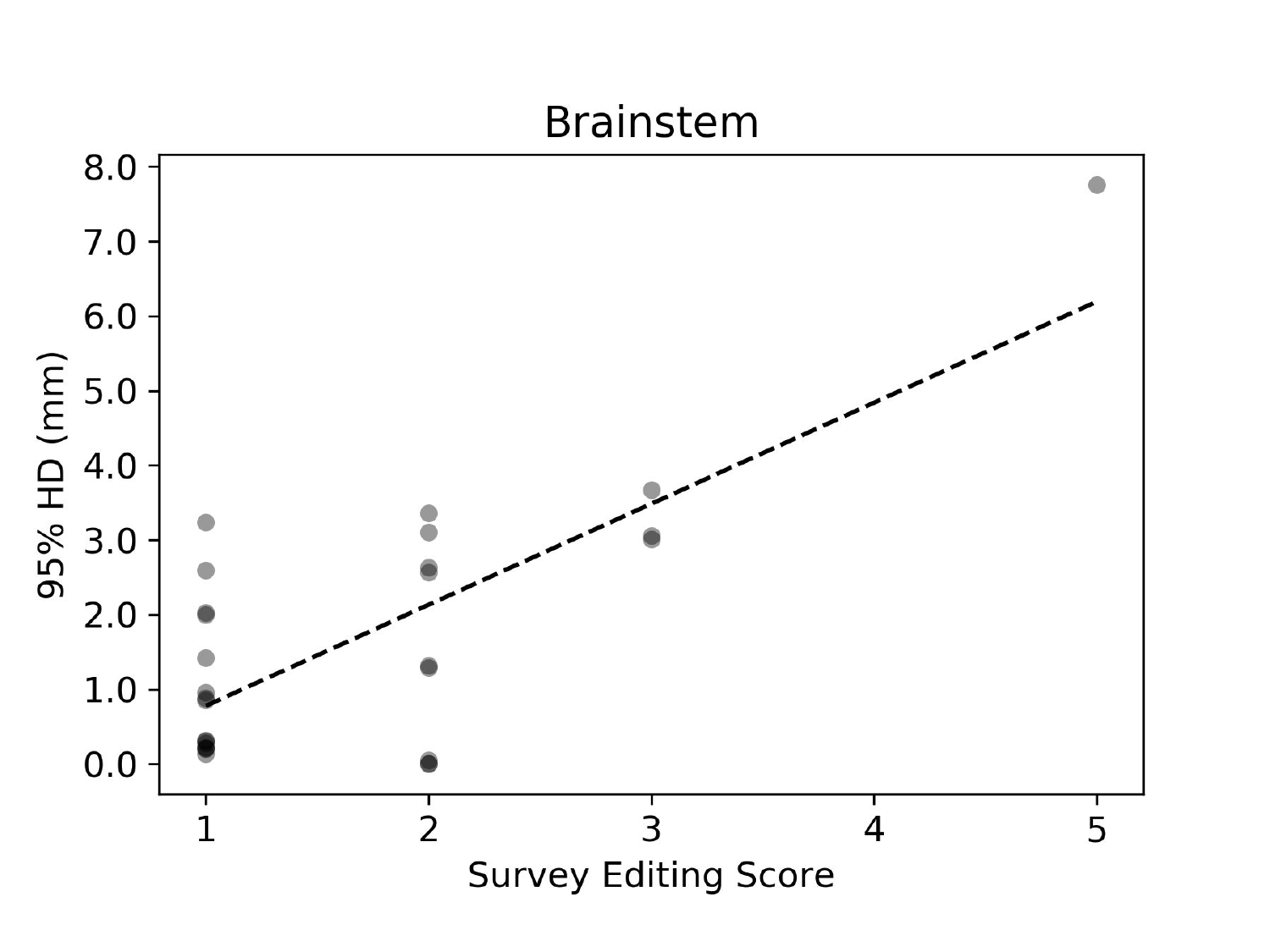

## Slide 4
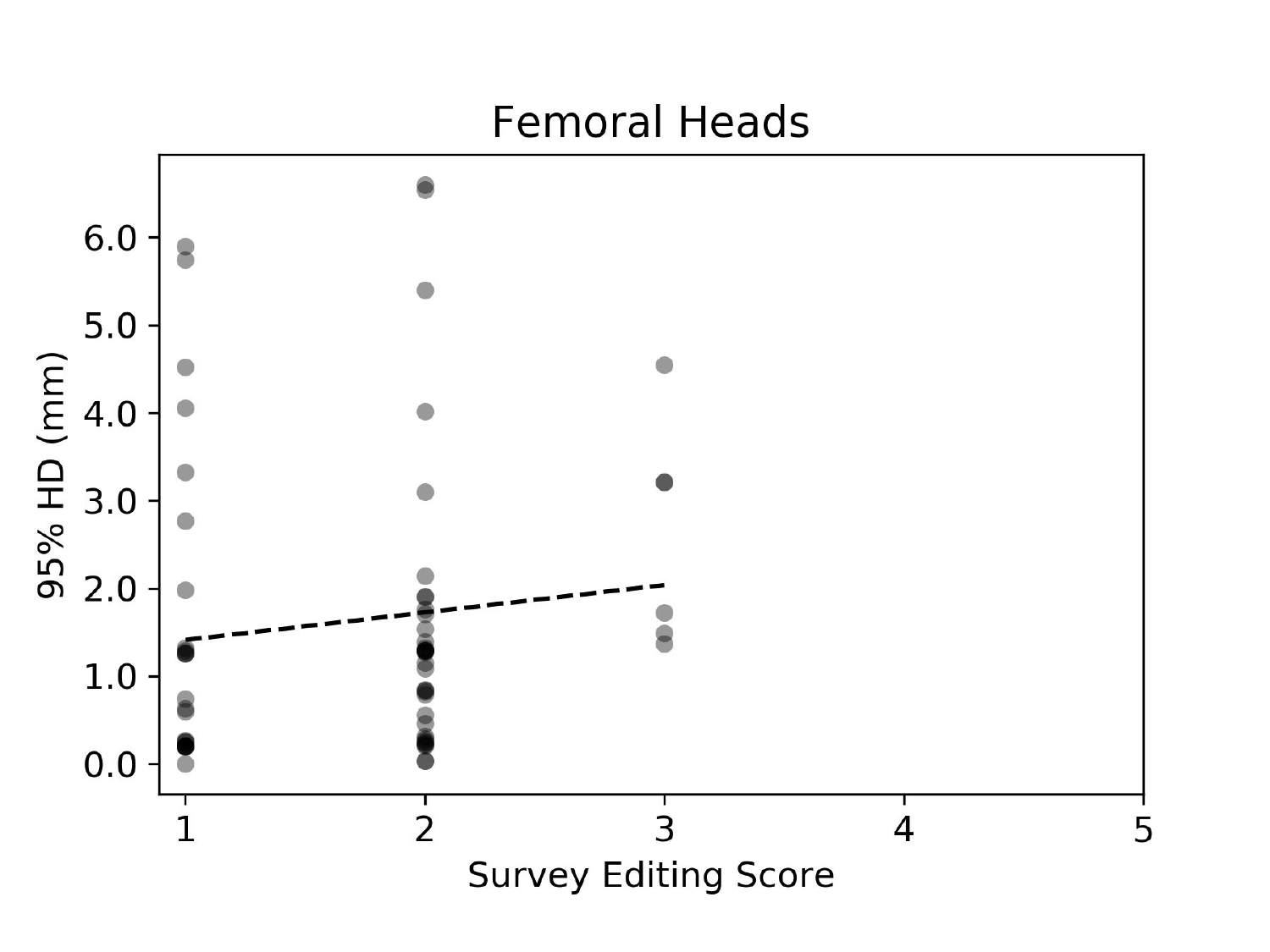

## Slide 5
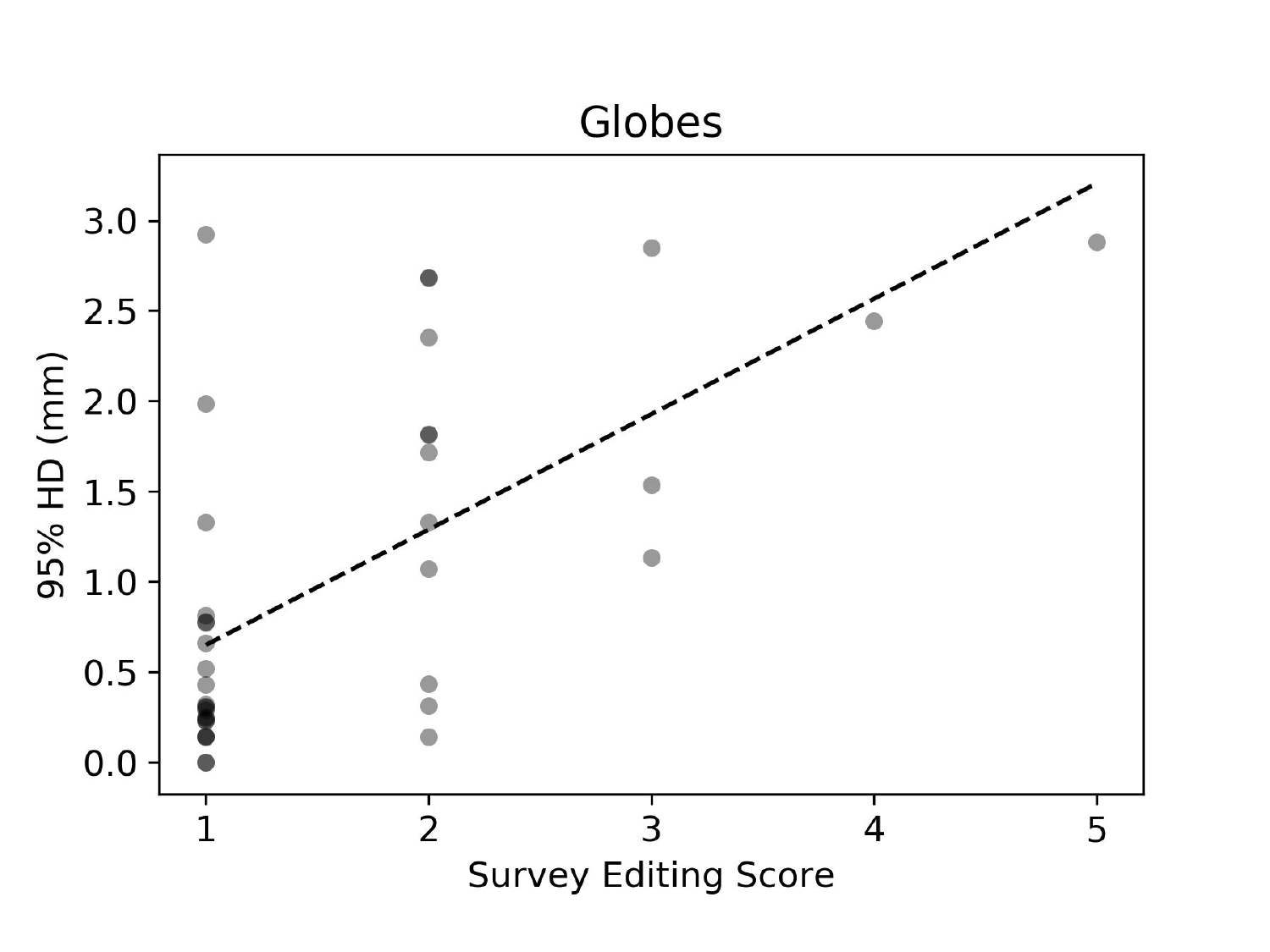

## Slide 6
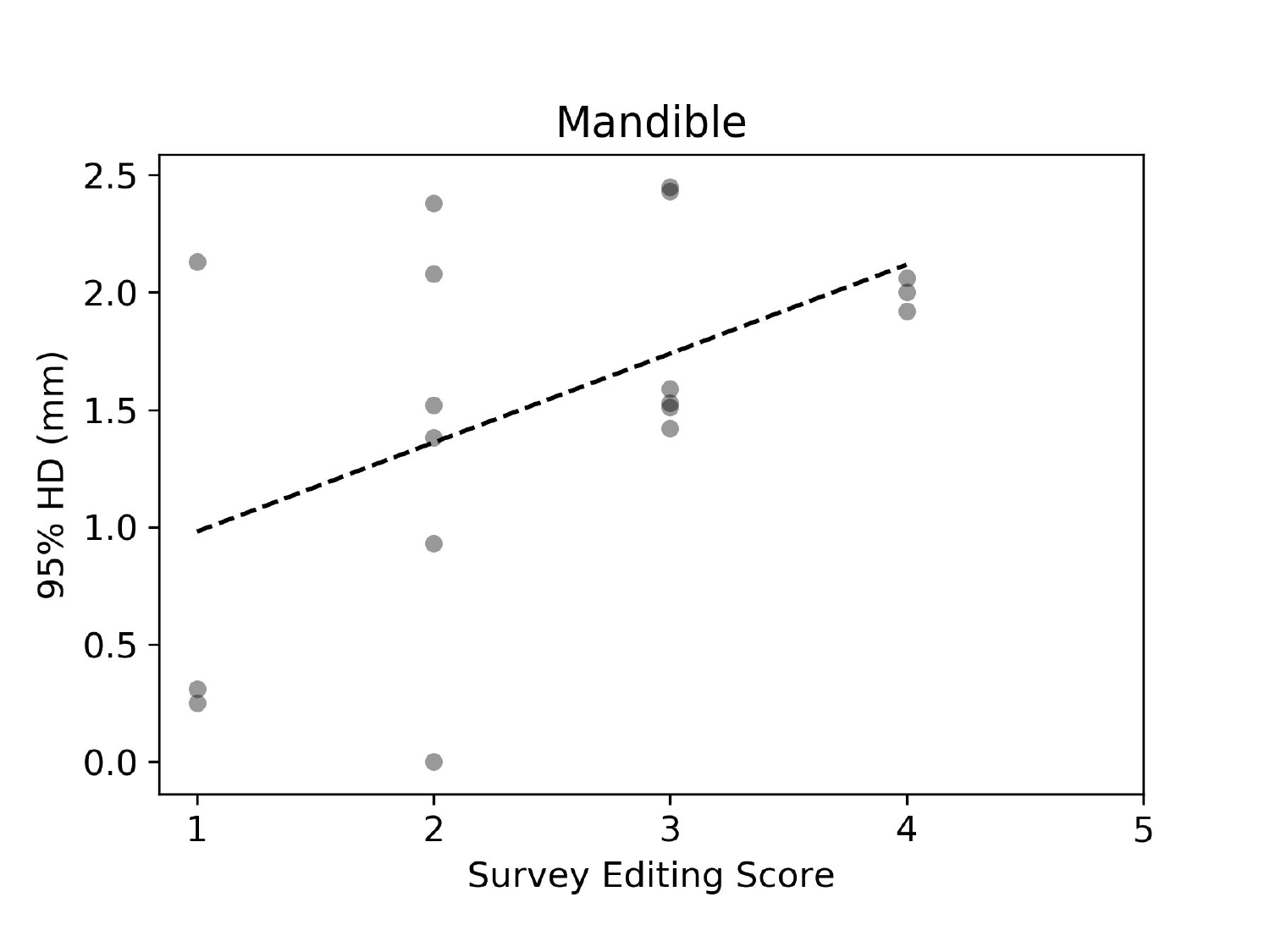

## Slide 7
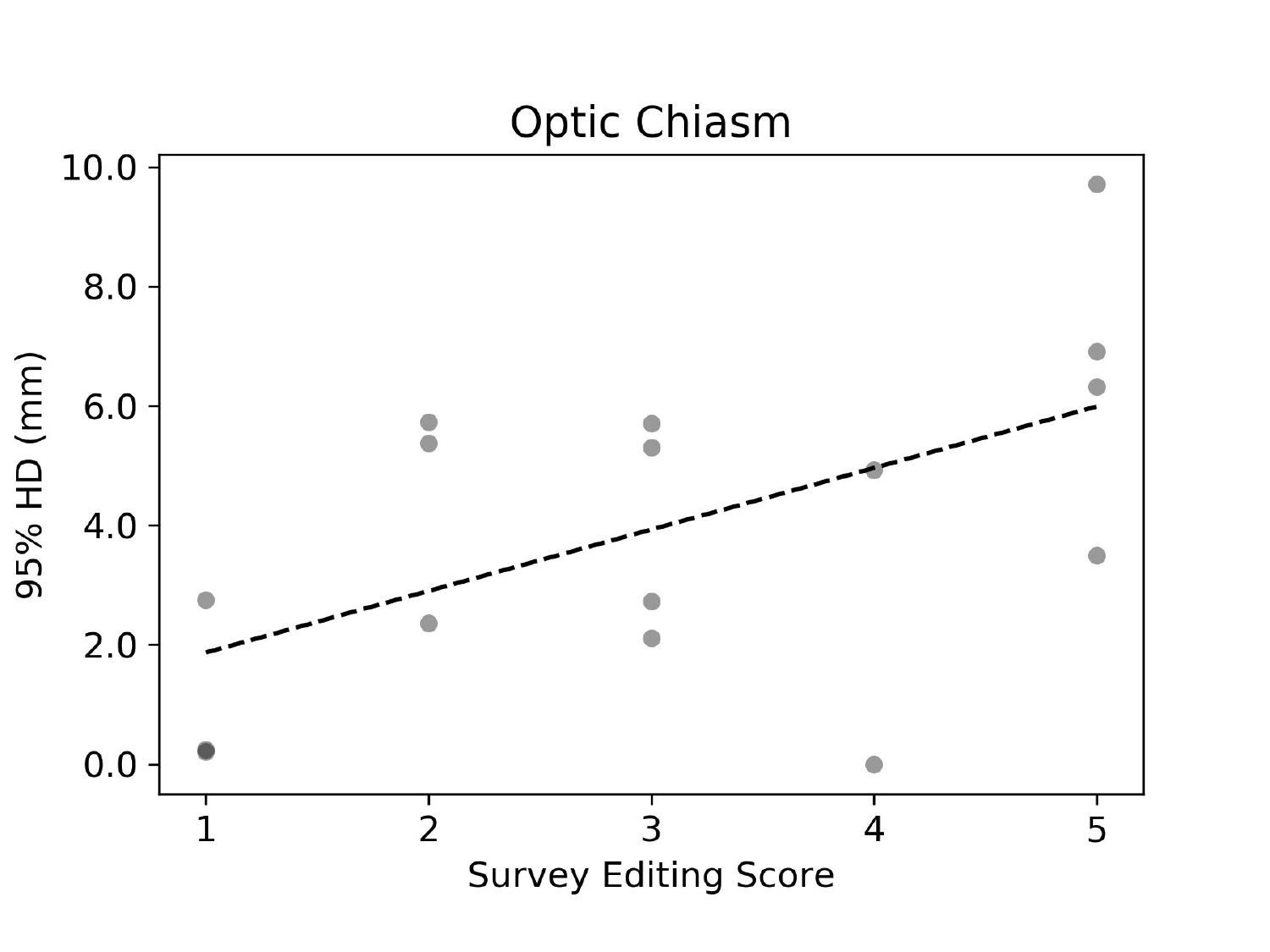

## Slide 8
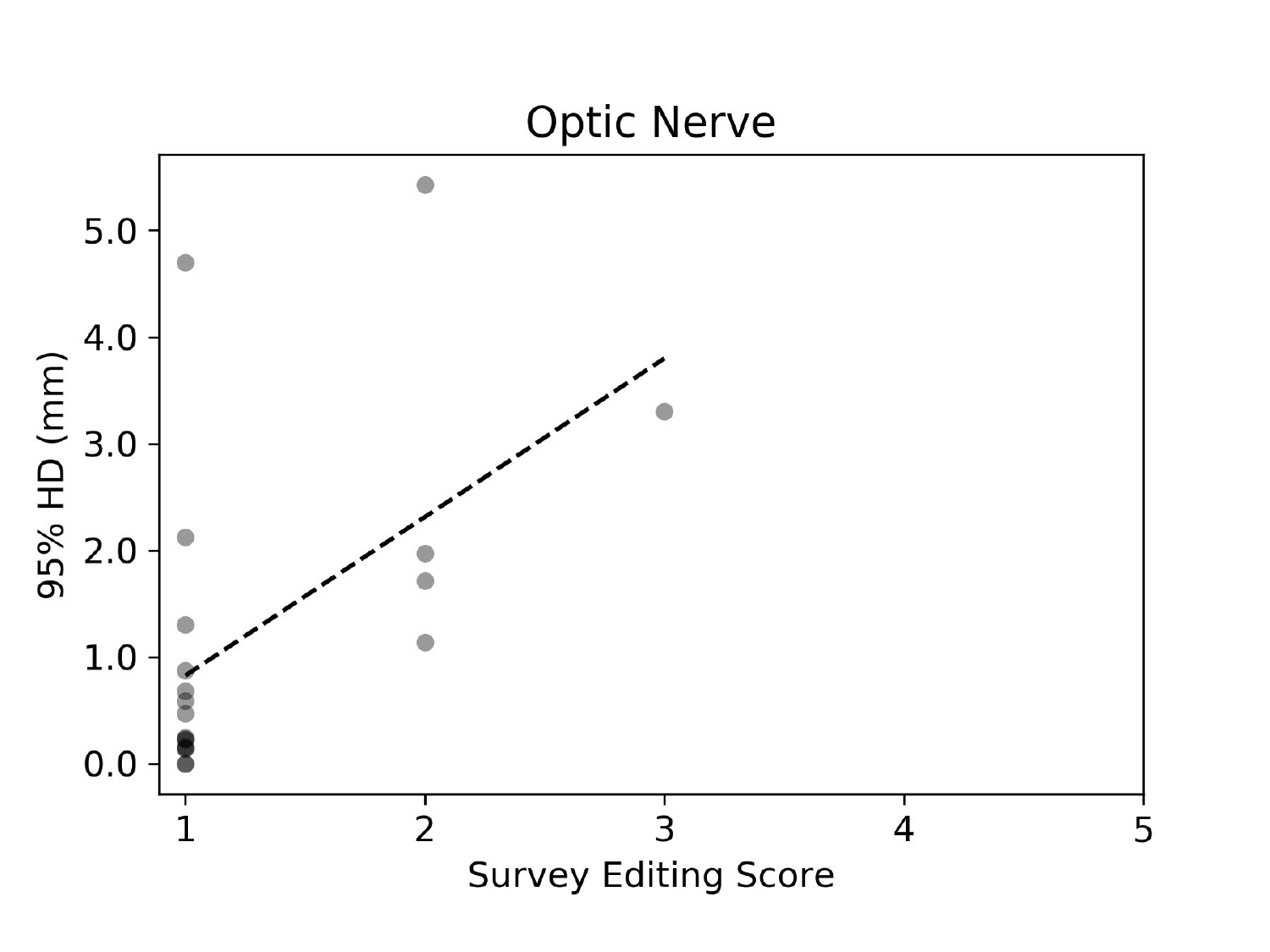

## Slide 9
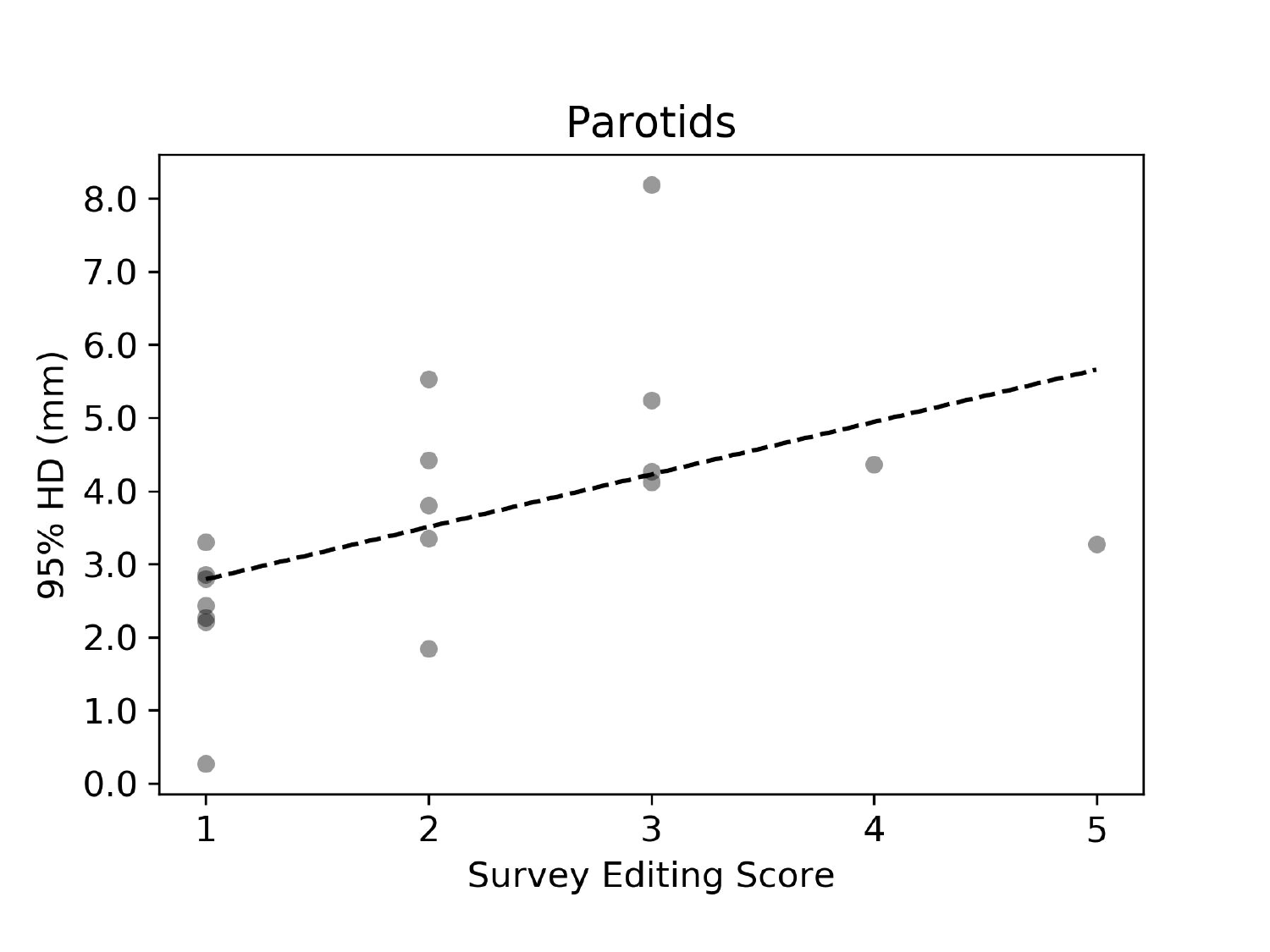

## Slide 10
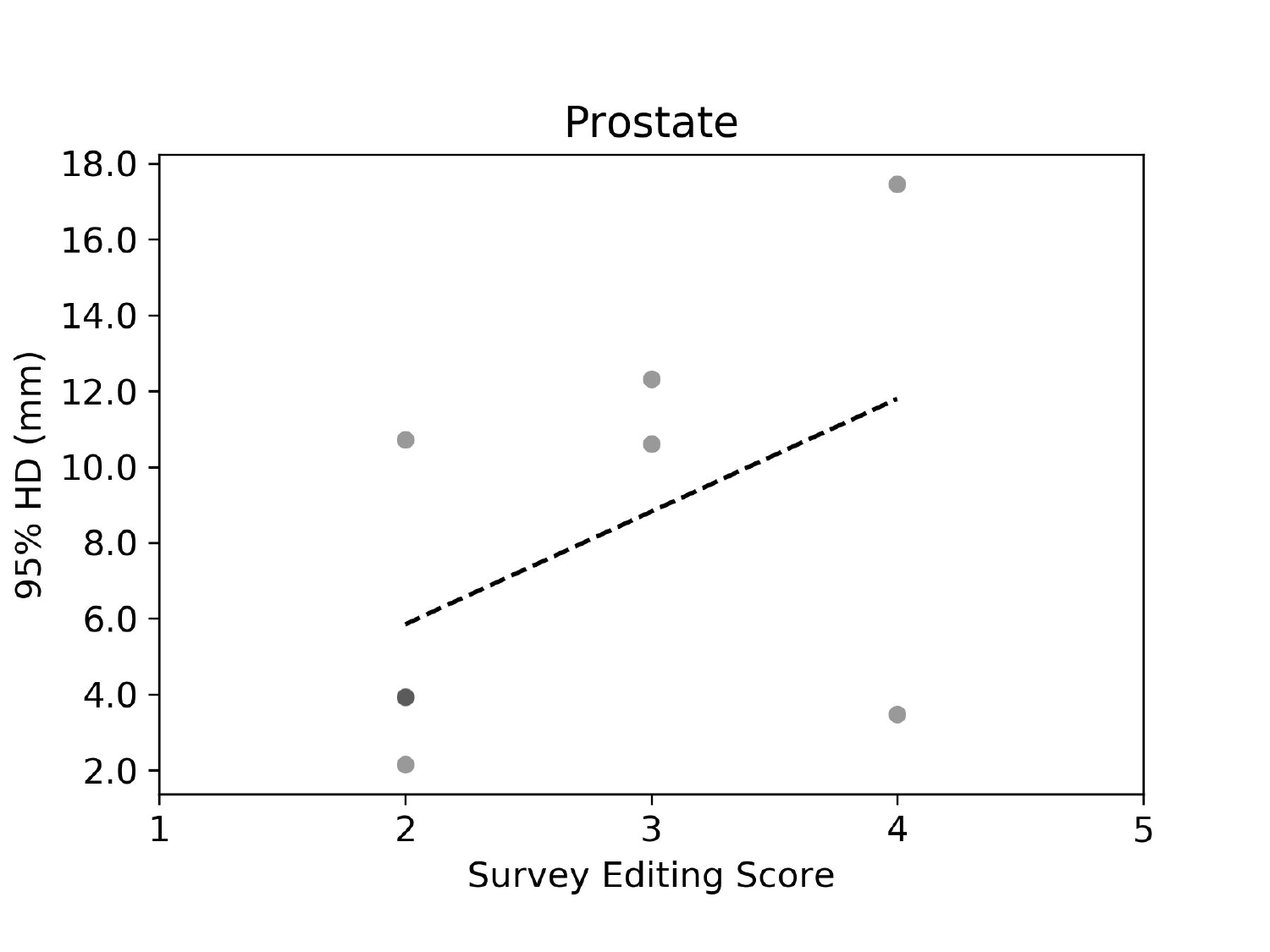

## Slide 11
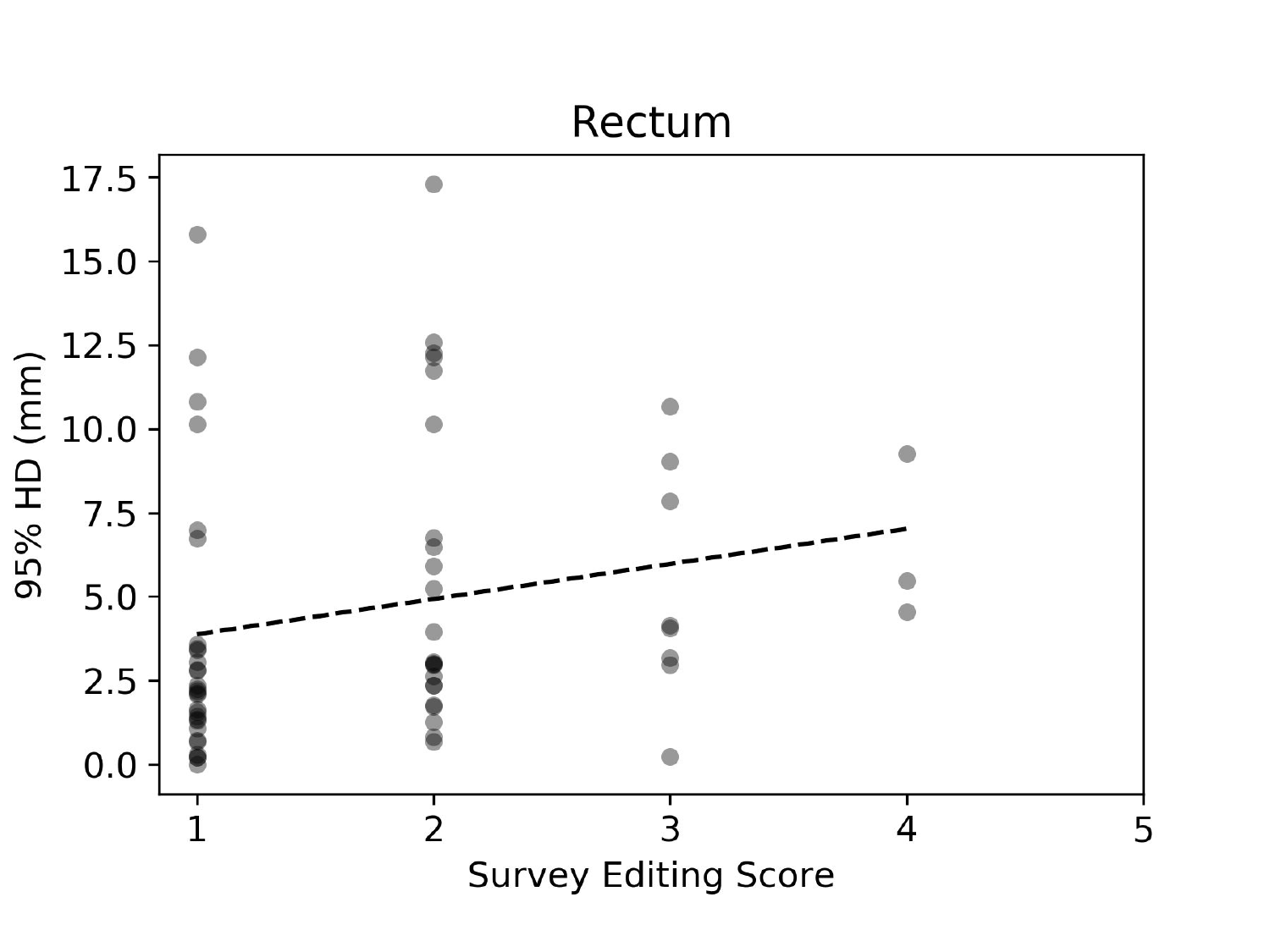

## Slide 12
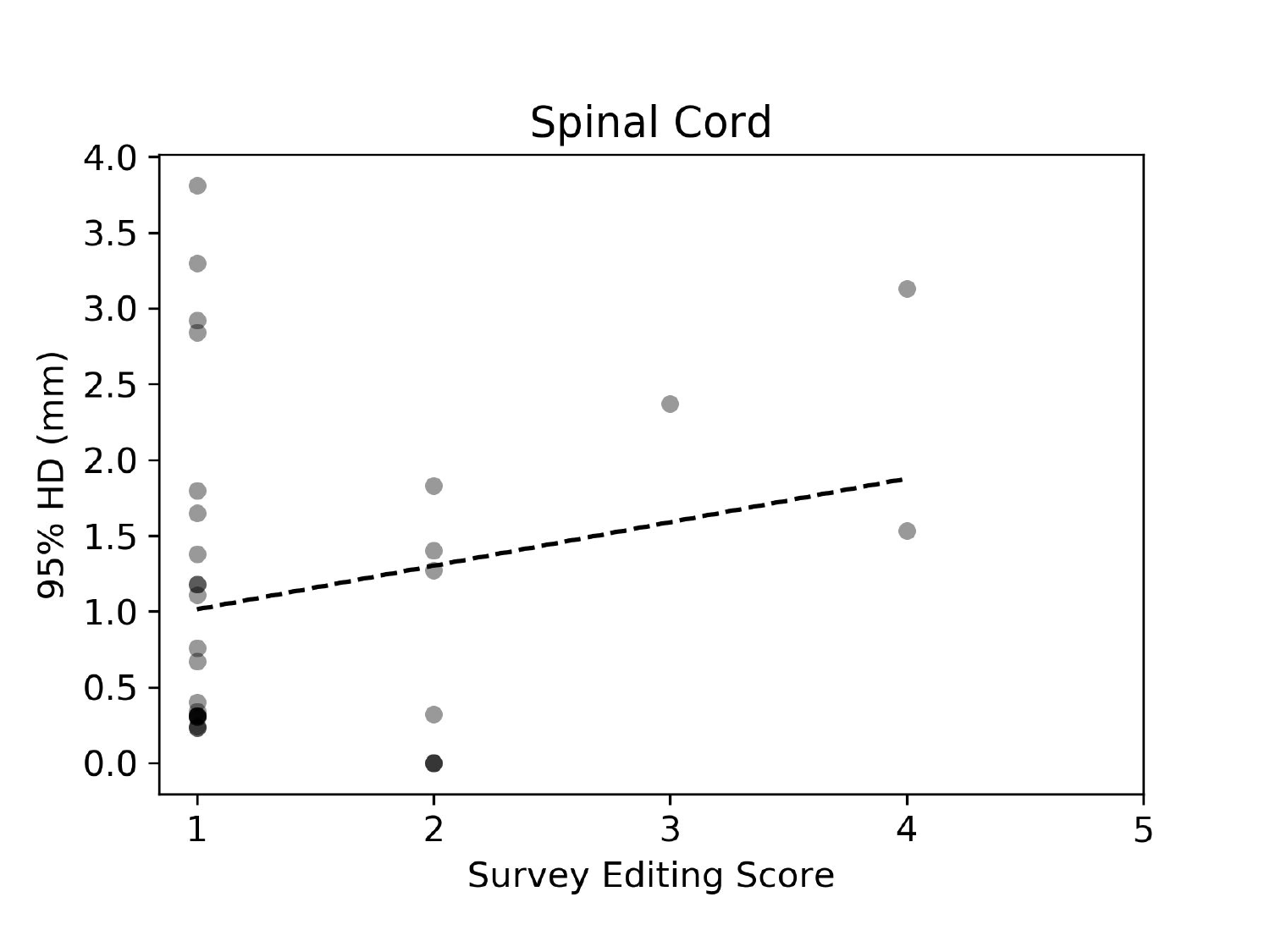

## Slide 13
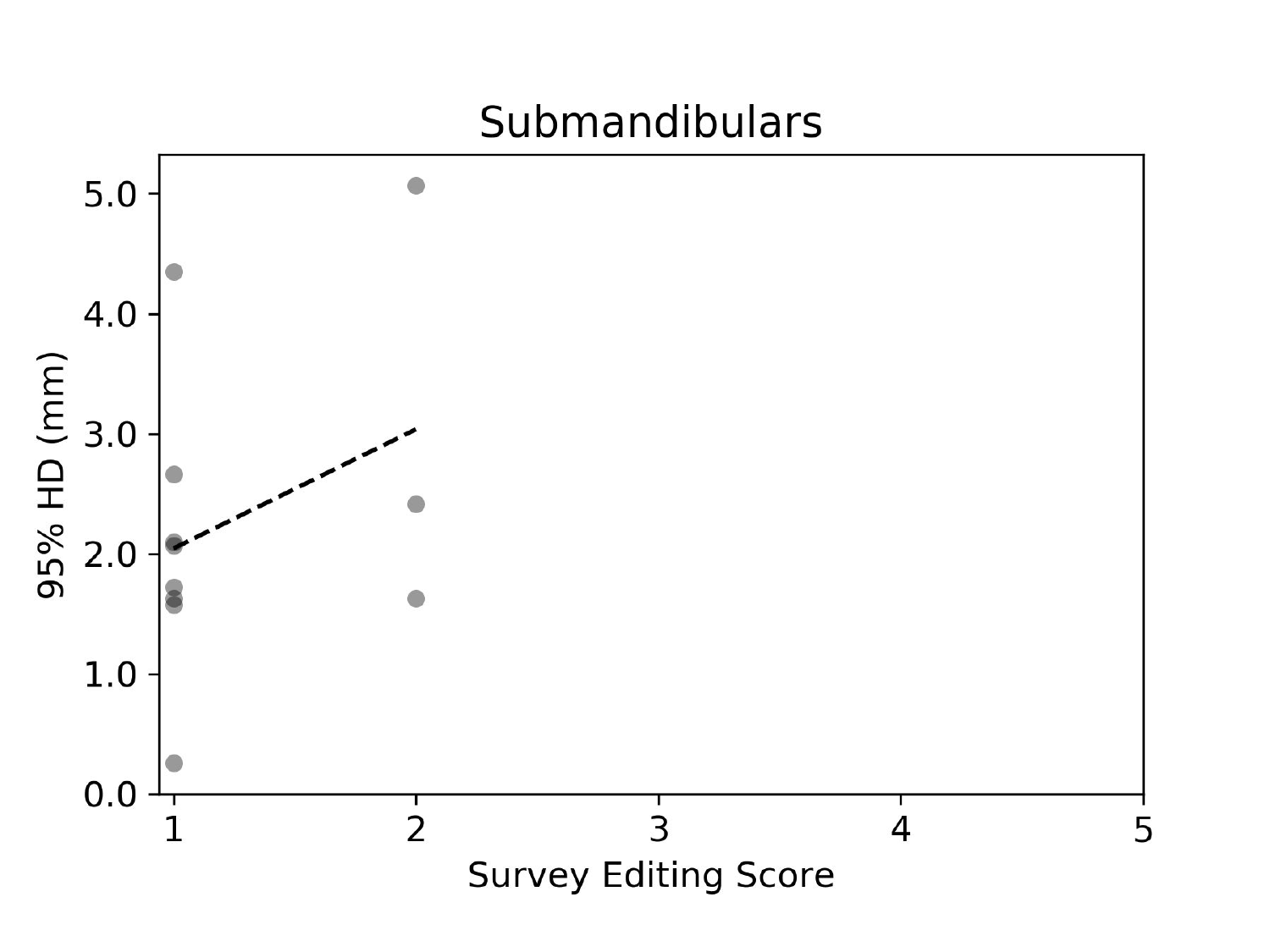

## Slide 14
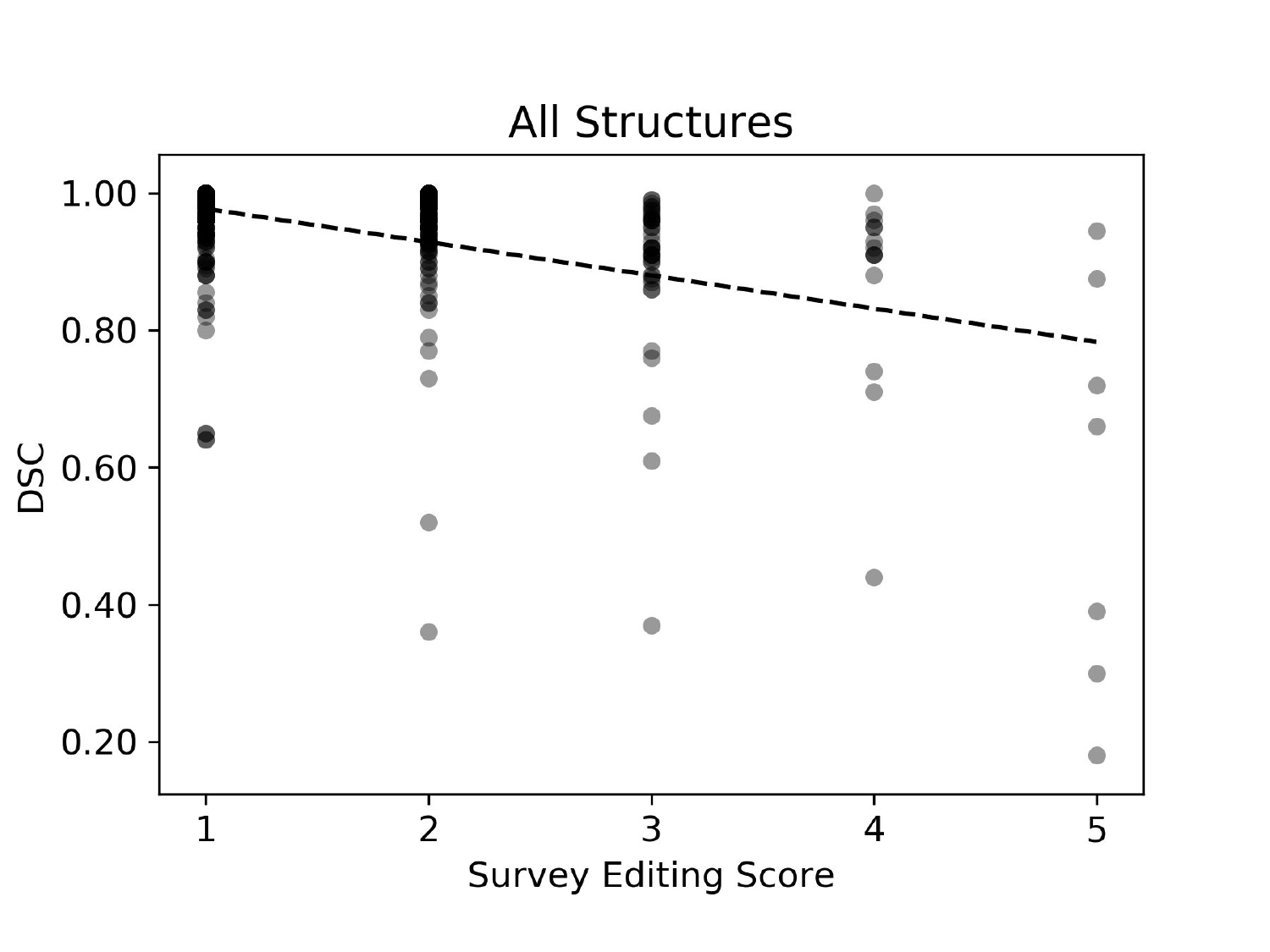

## Slide 15
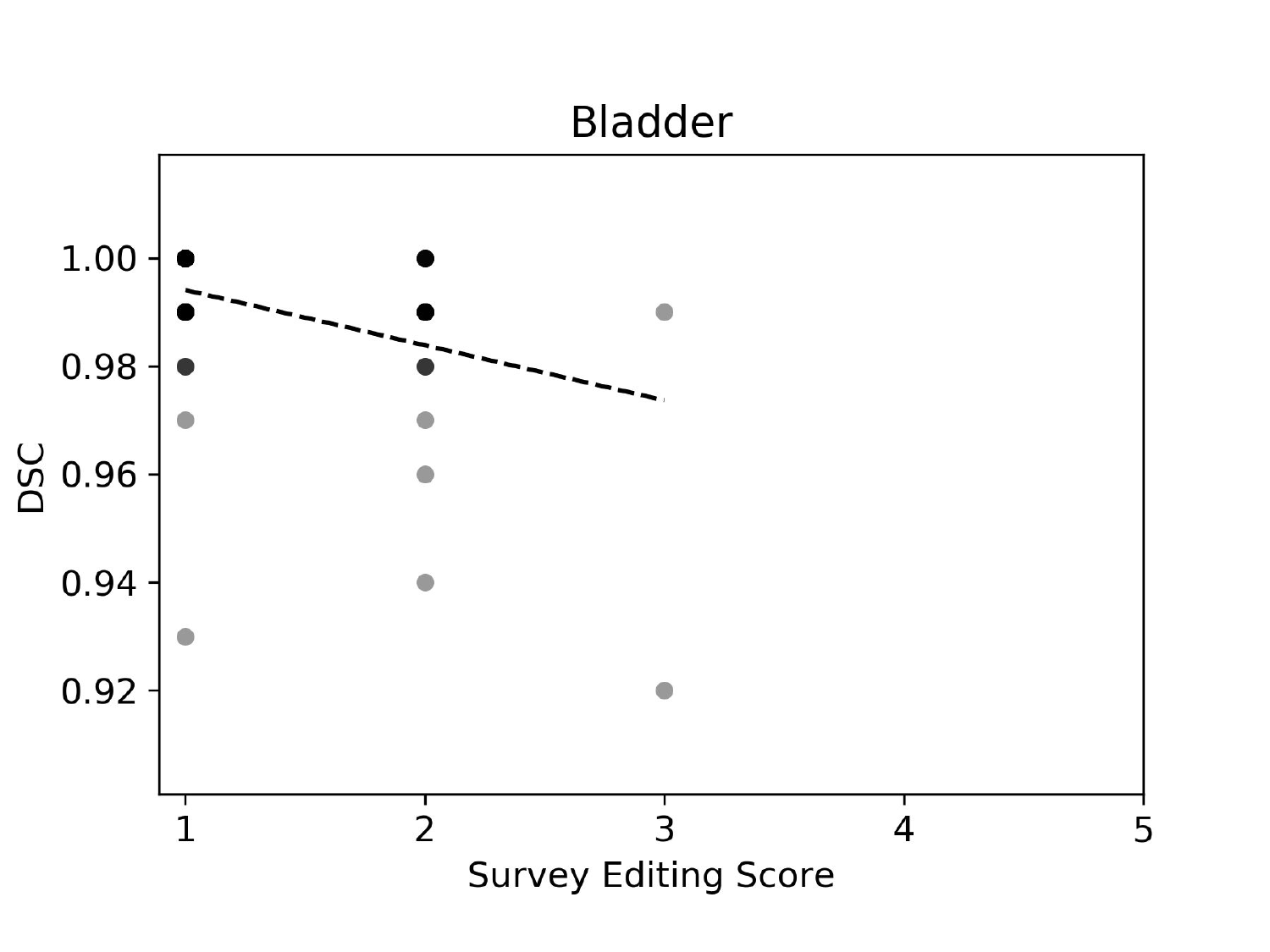

## Slide 16
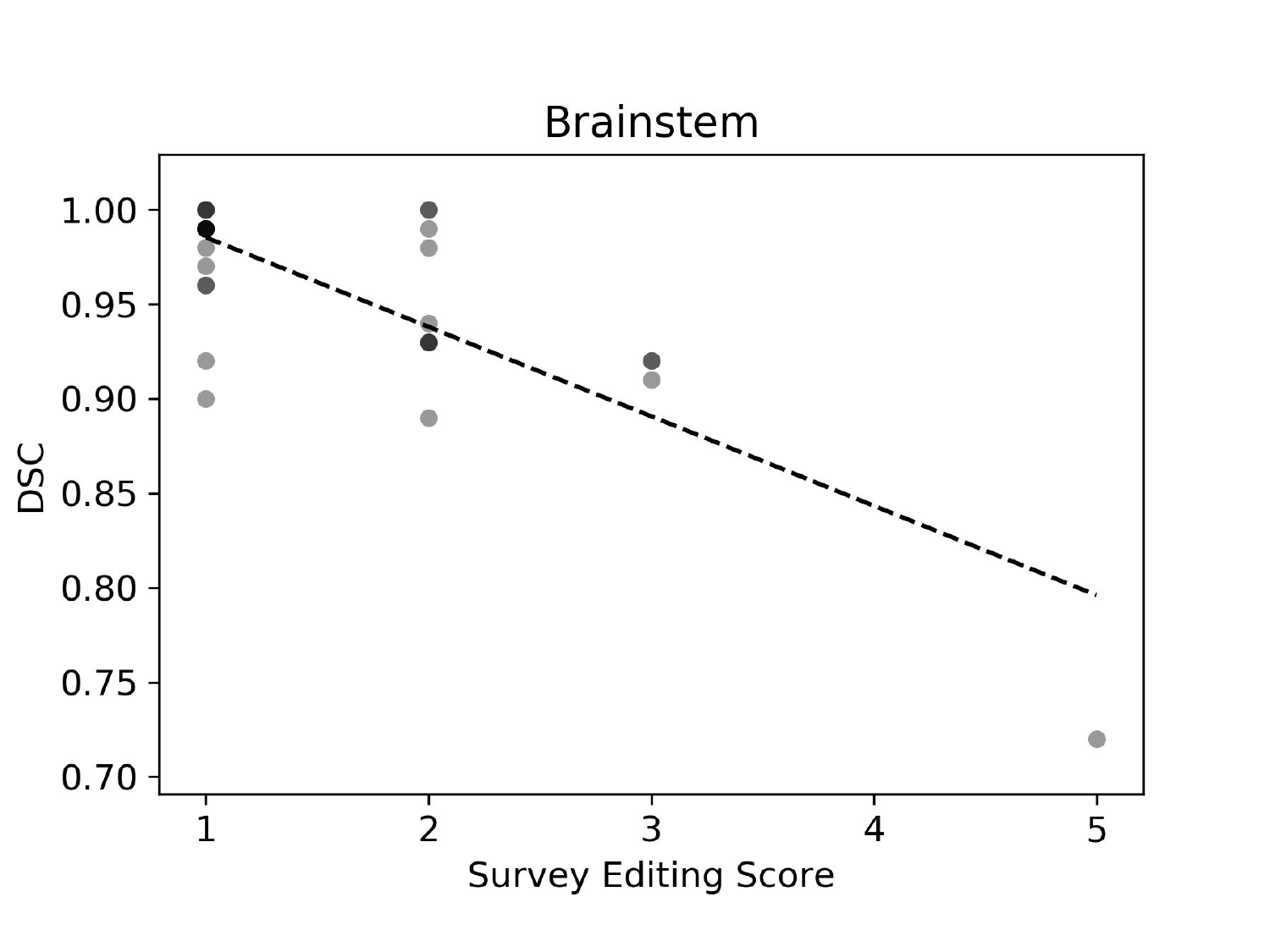

## Slide 17
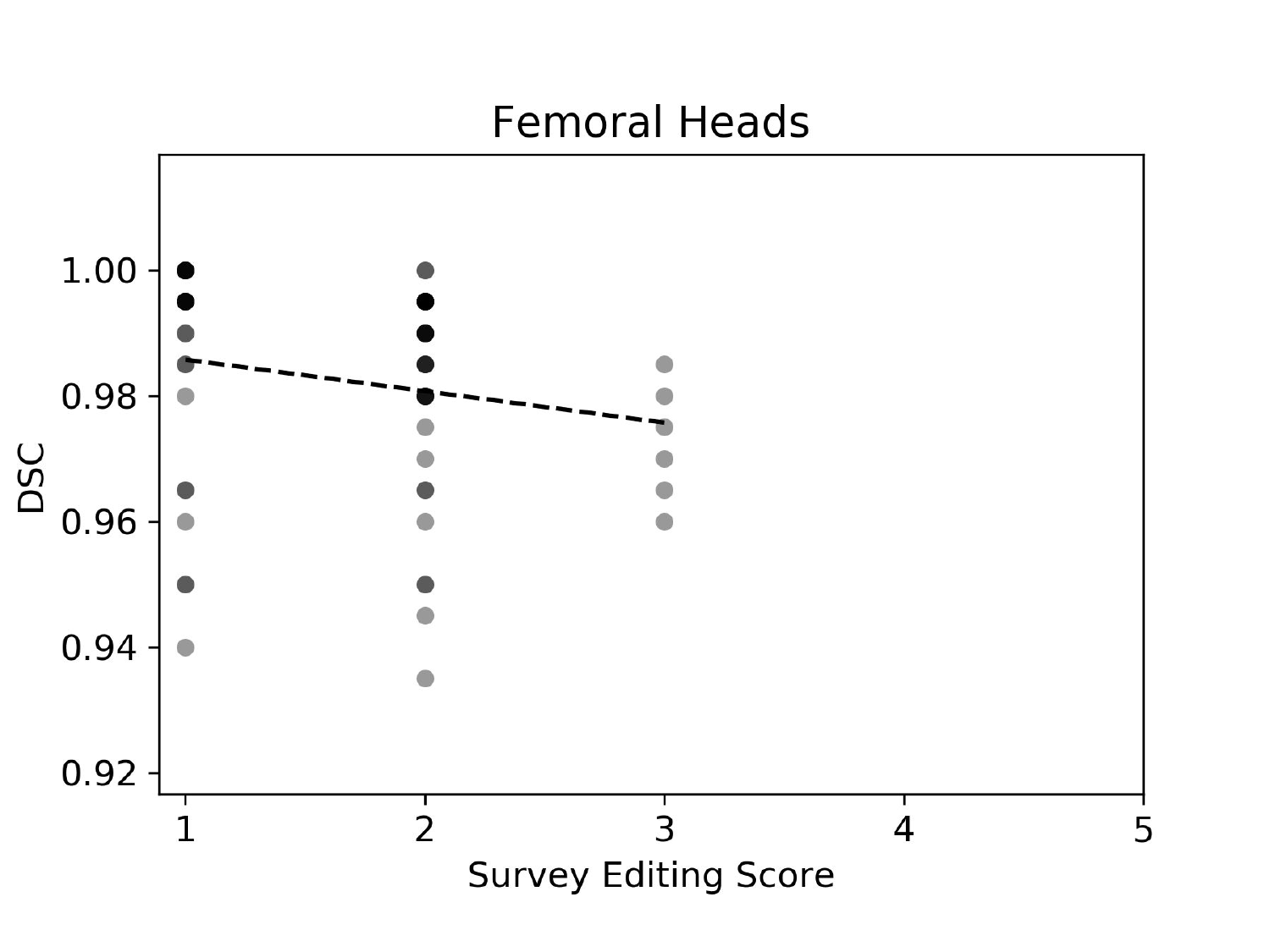

## Slide 18
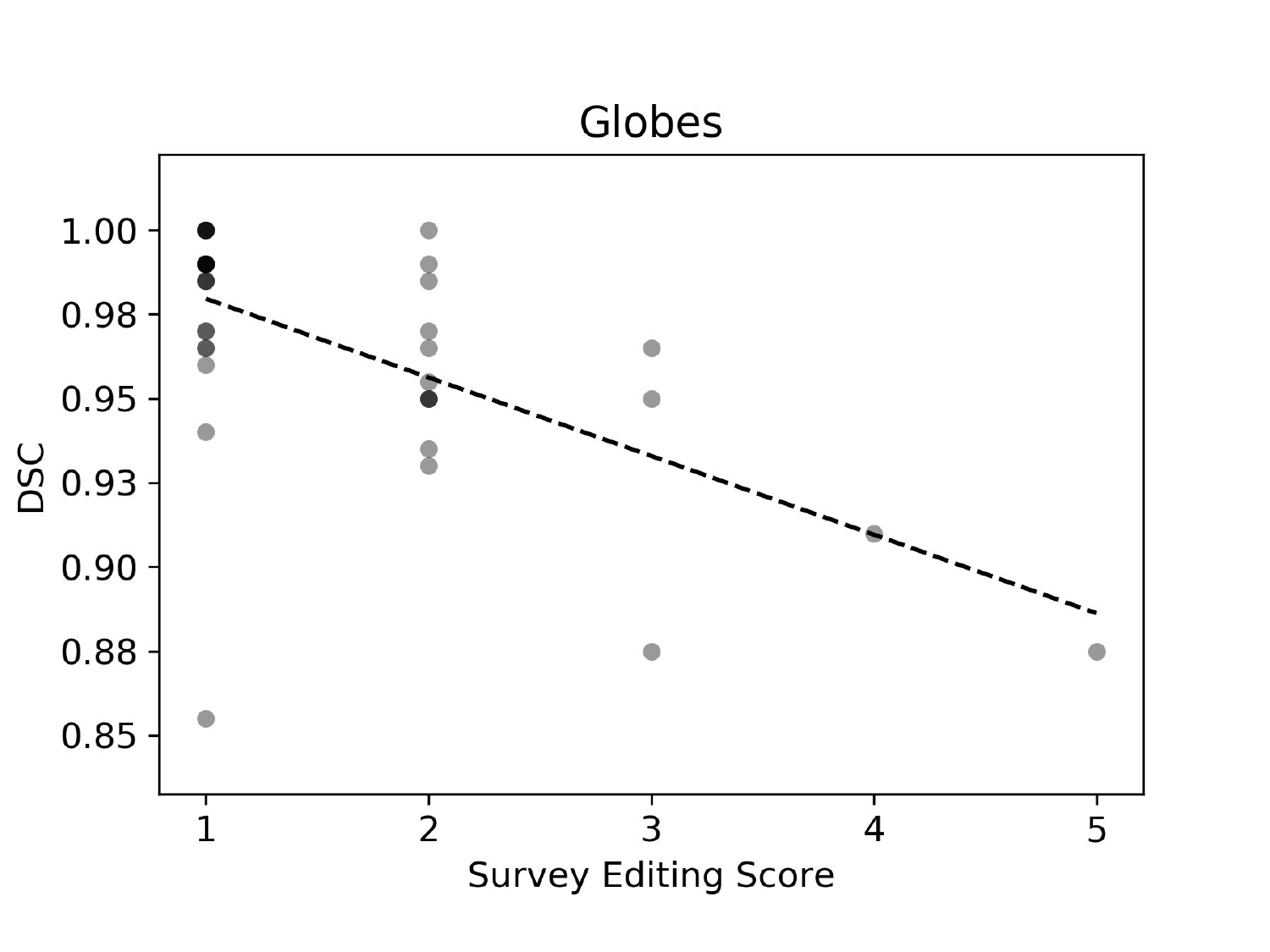

## Slide 19
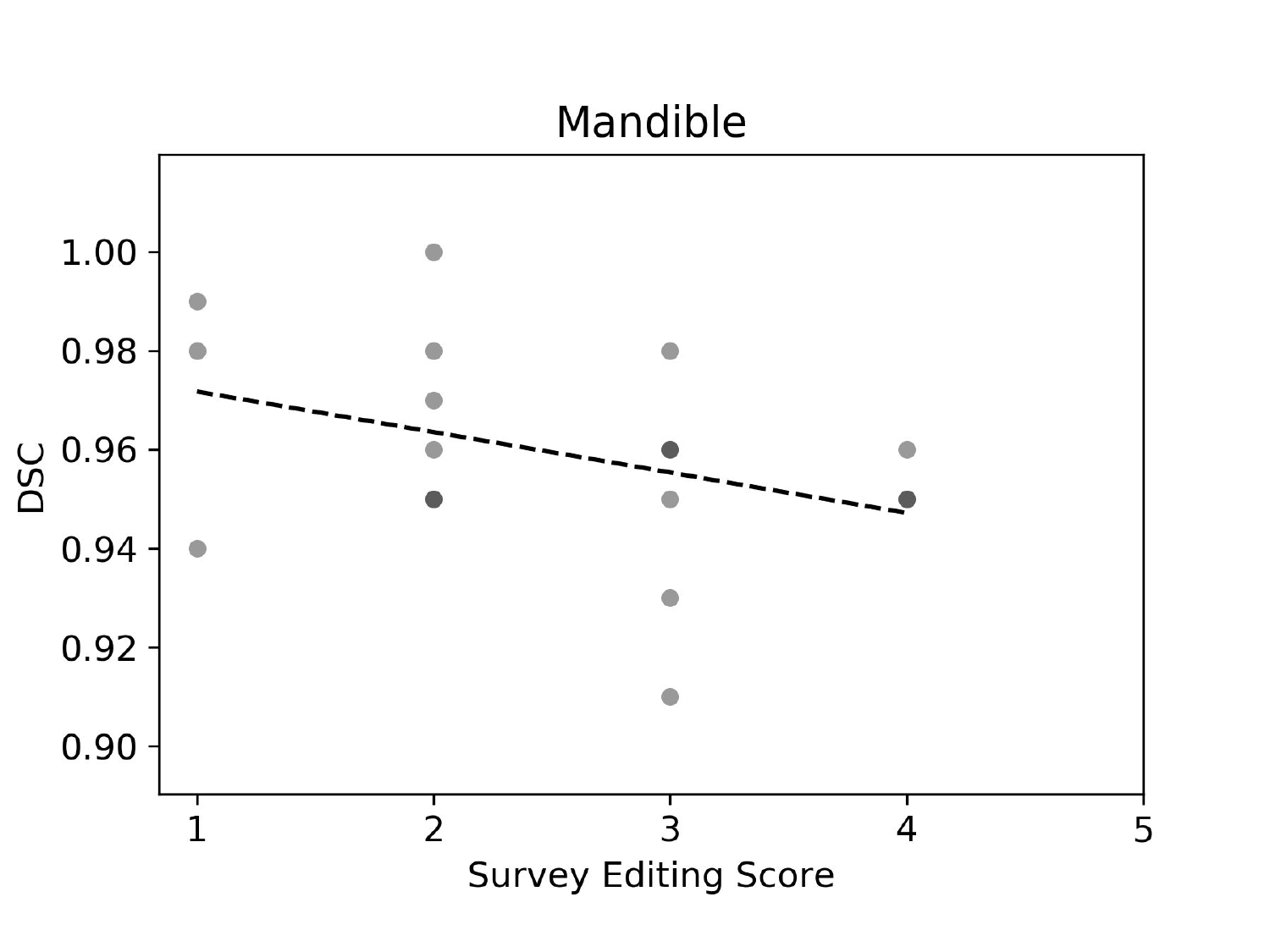

## Slide 20
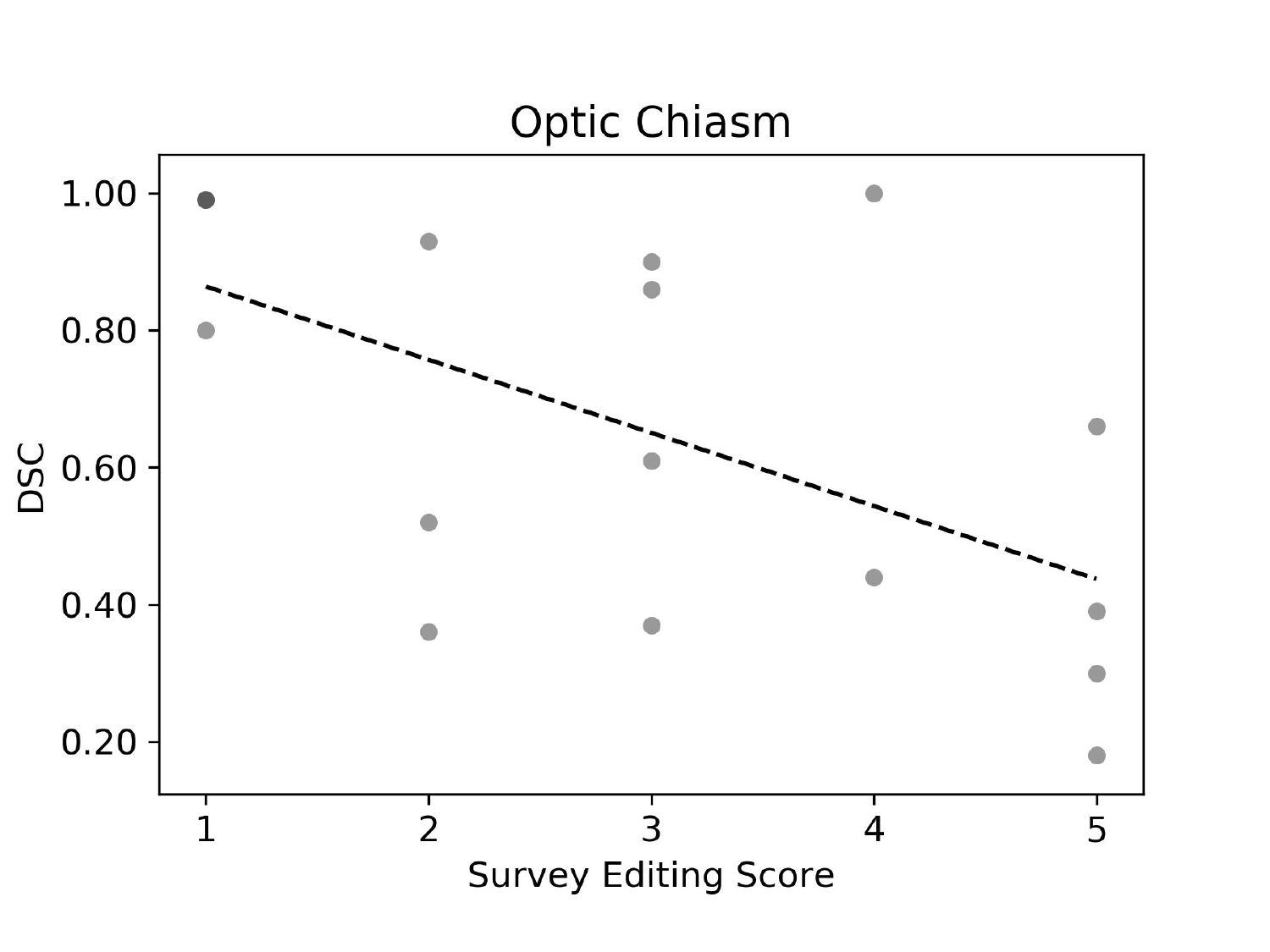

## Slide 21
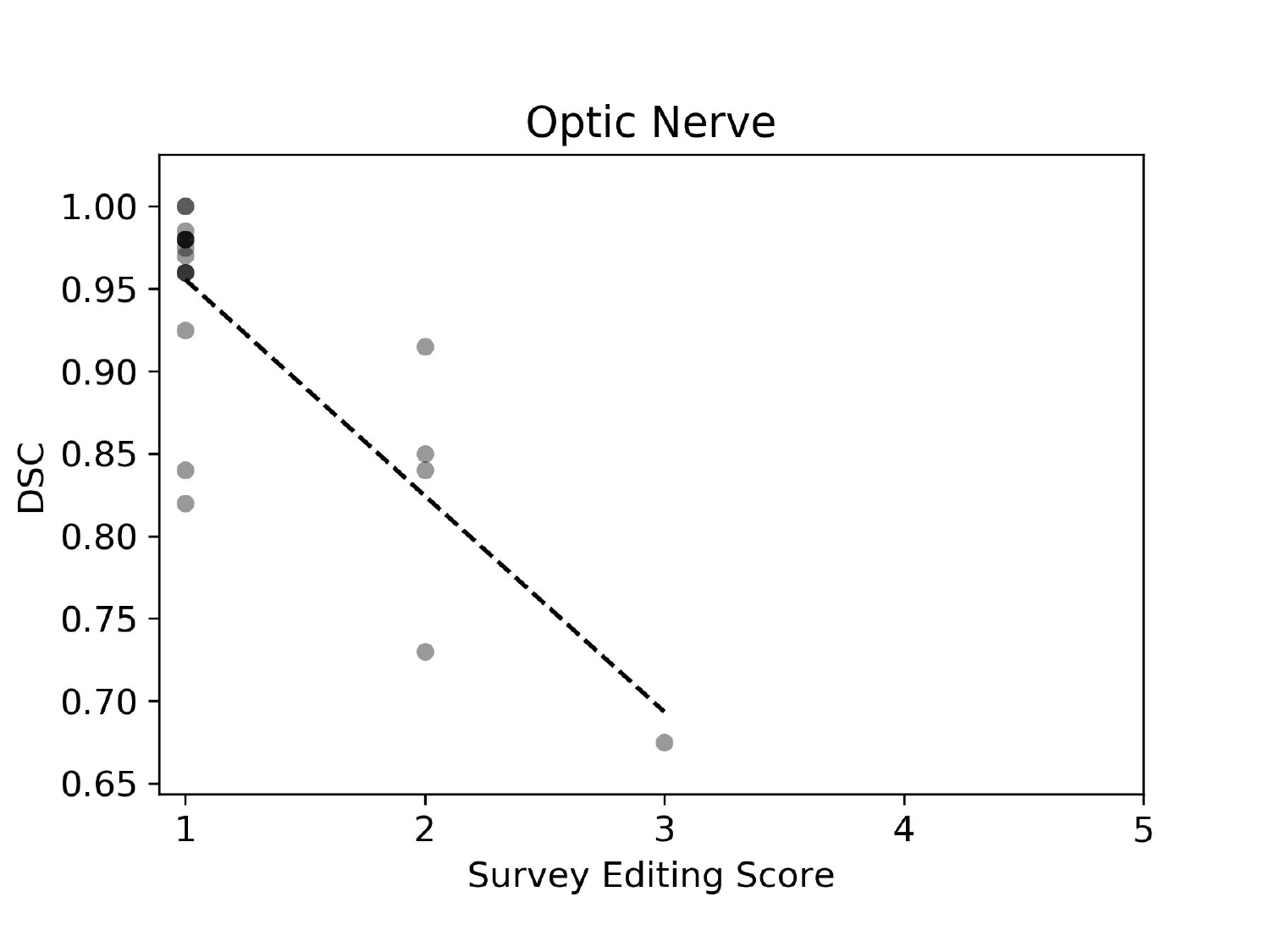

## Slide 22
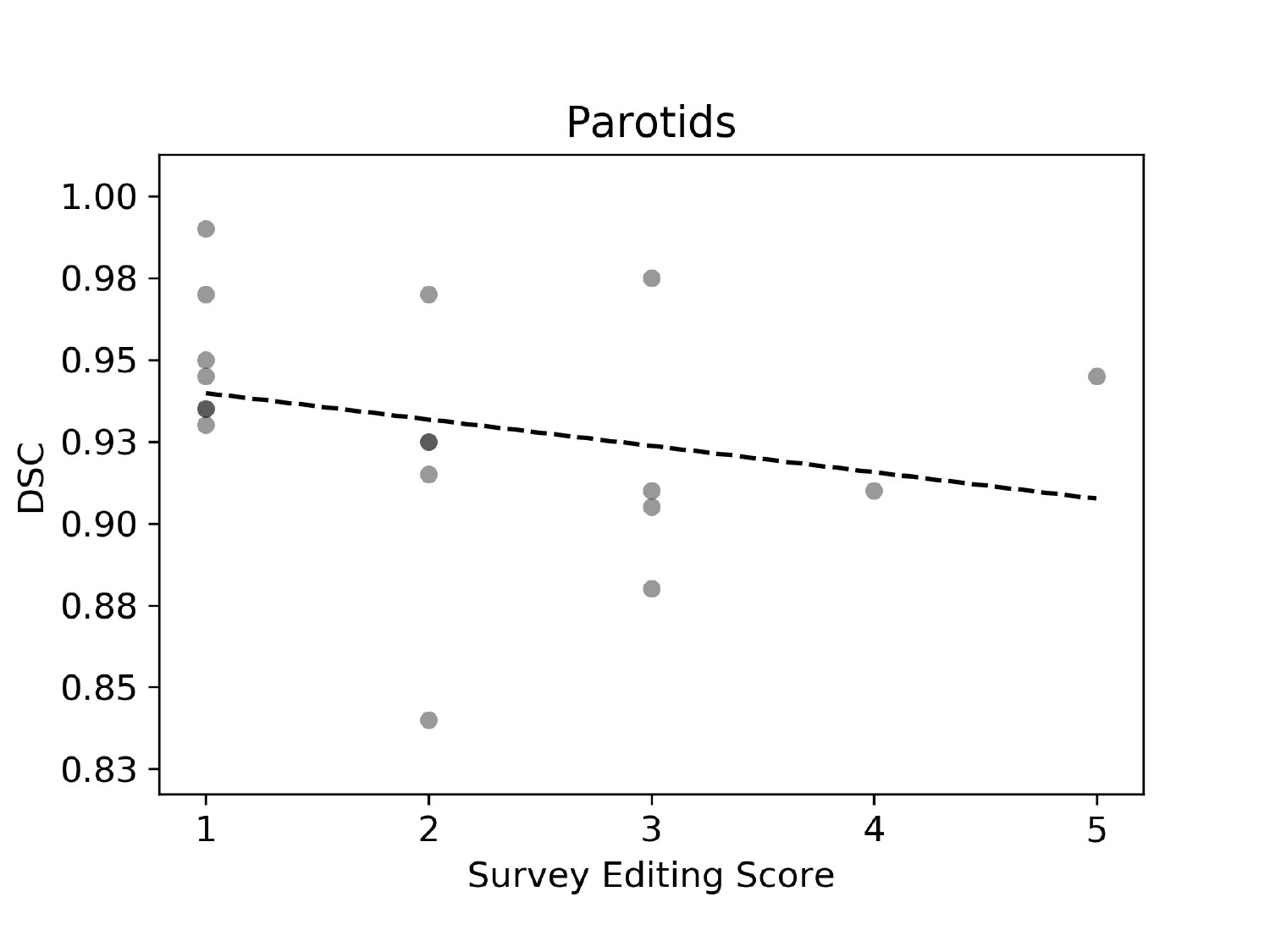

## Slide 23
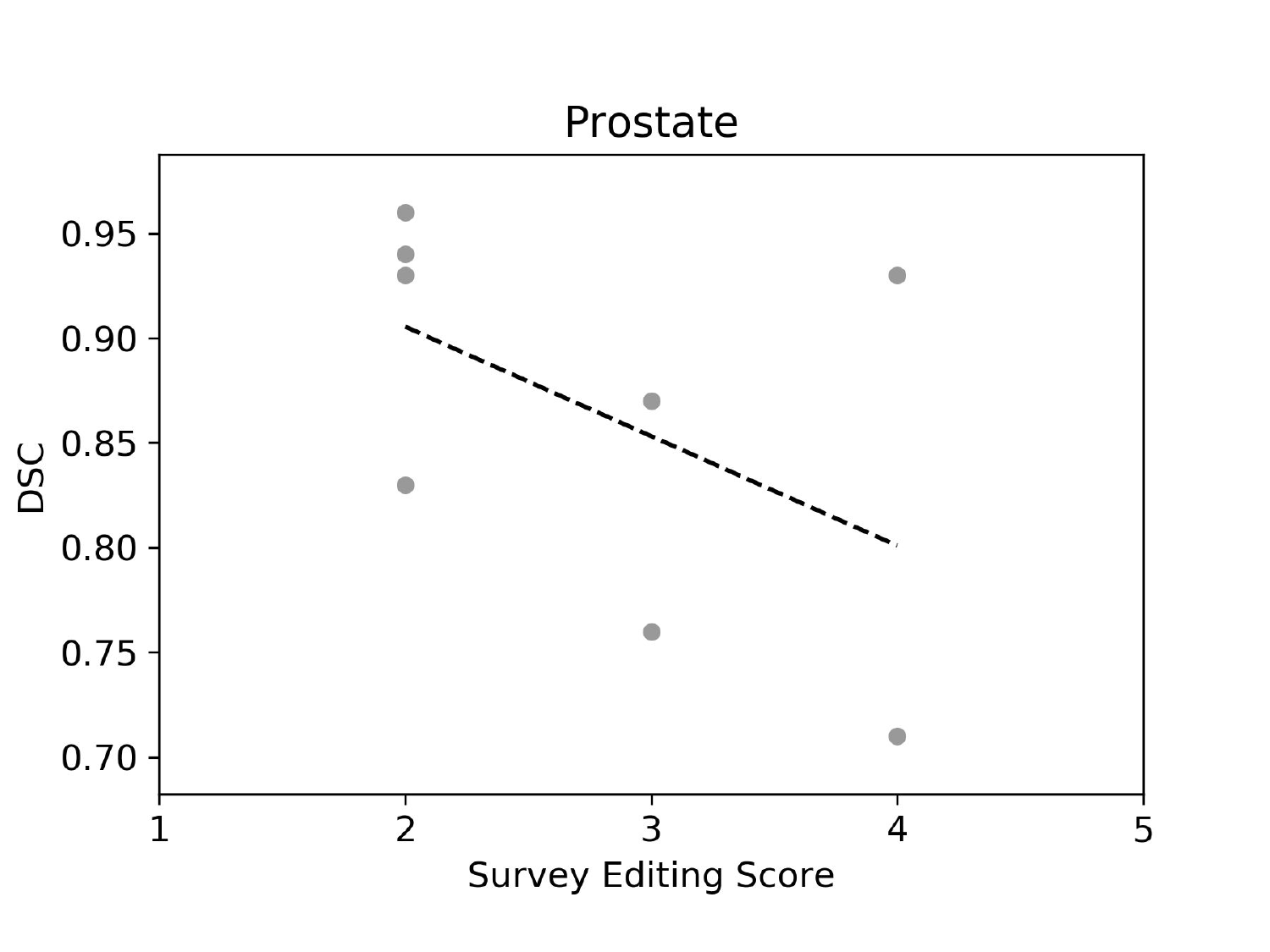

## Slide 24
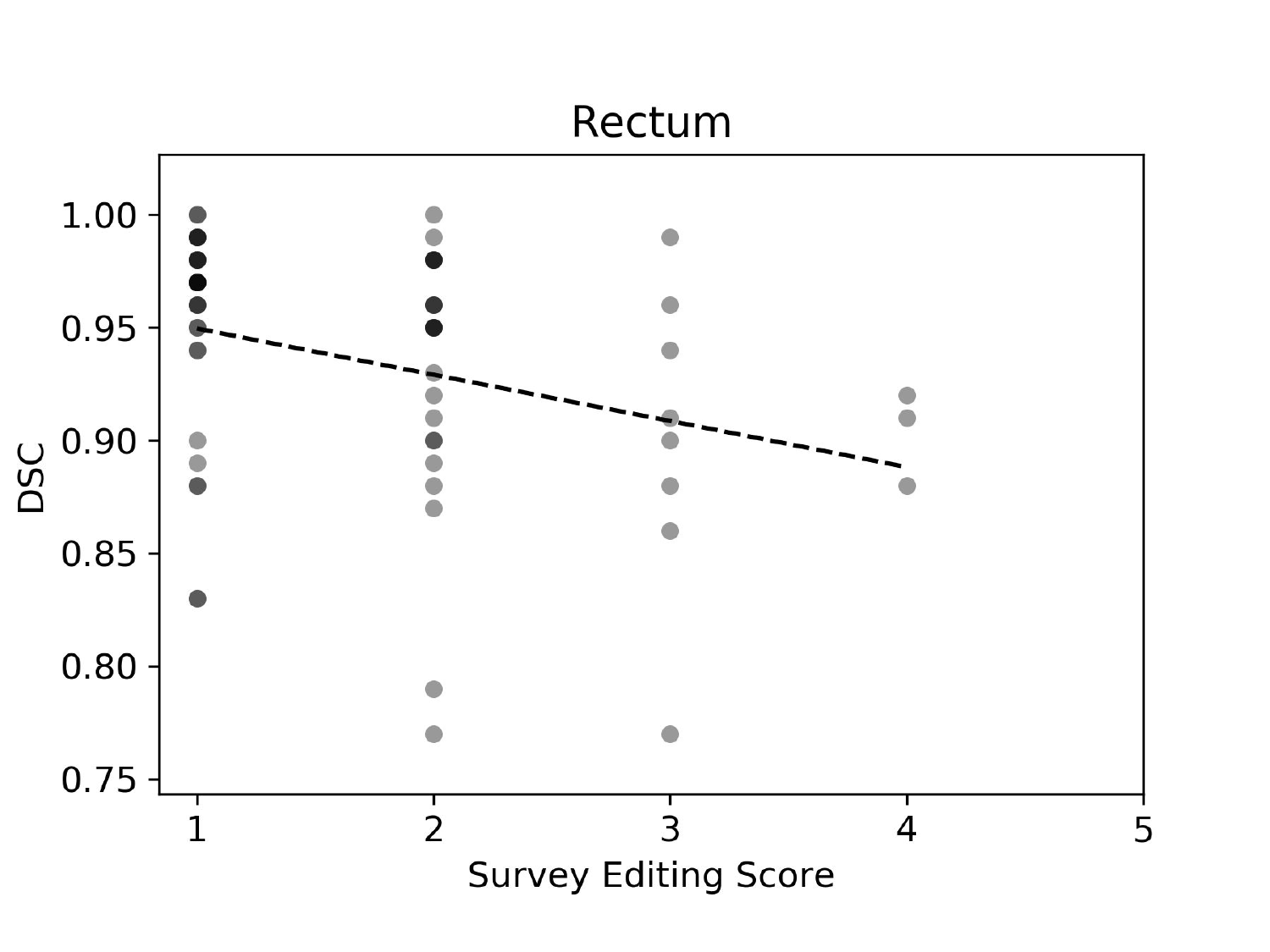

## Slide 25
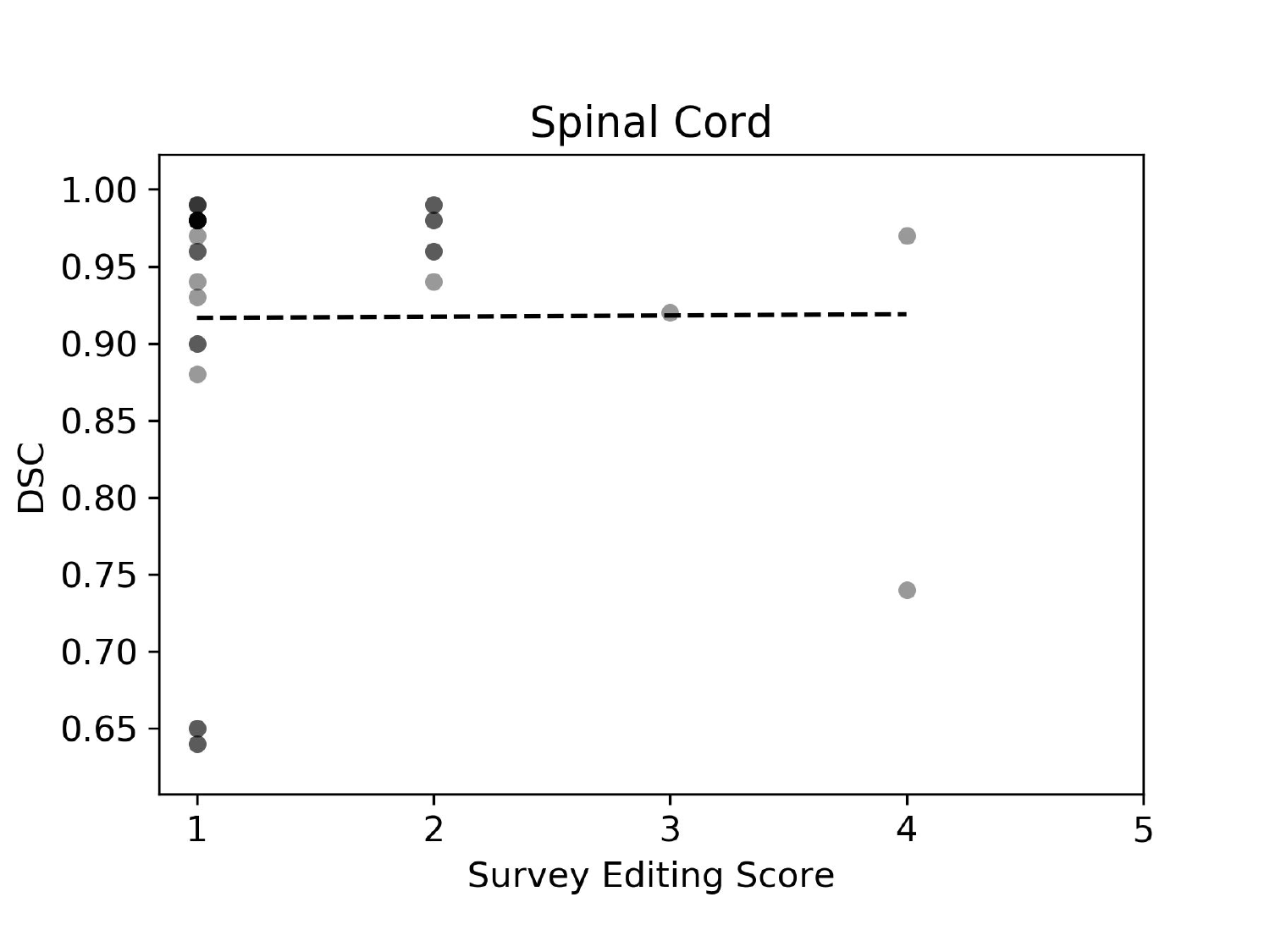

## Slide 26
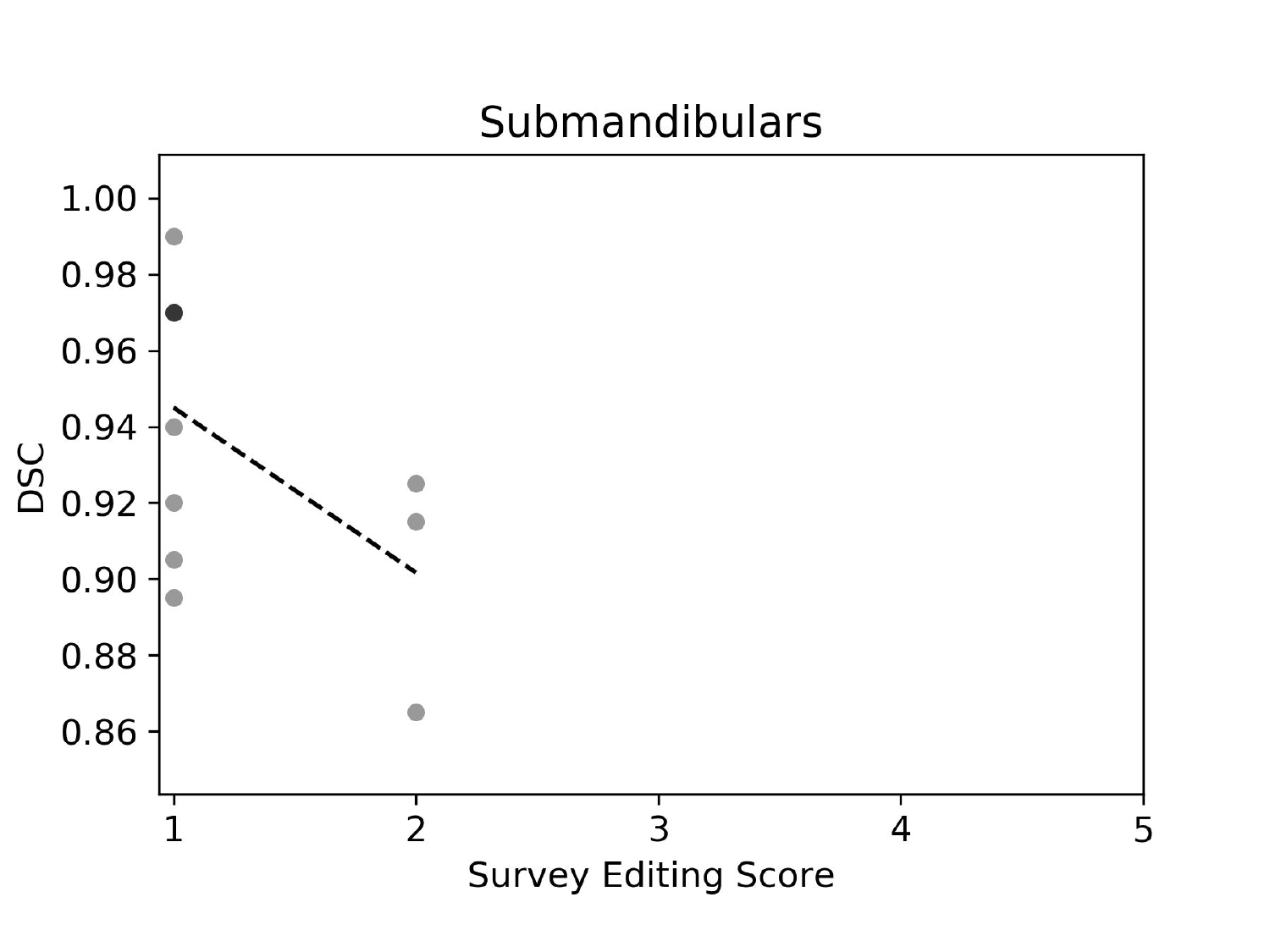

Supplement: Supplementary file 1 — Additional file 1. Supplementary tables. [file 13014_2021_1831_MOESM1_ESM.pptx]
