# Supplementary material for: Implementation of deep learning-based auto-segmentation for radiotherapy planning structures: a workflow study at two cancer centers
Source: Radiat Oncol. 2021 Jun 8;16:101. doi: 10.1186/s13014-021-01831-4 (PMC8186196; doi:10.1186/s13014-021-01831-4)

## Slide 1
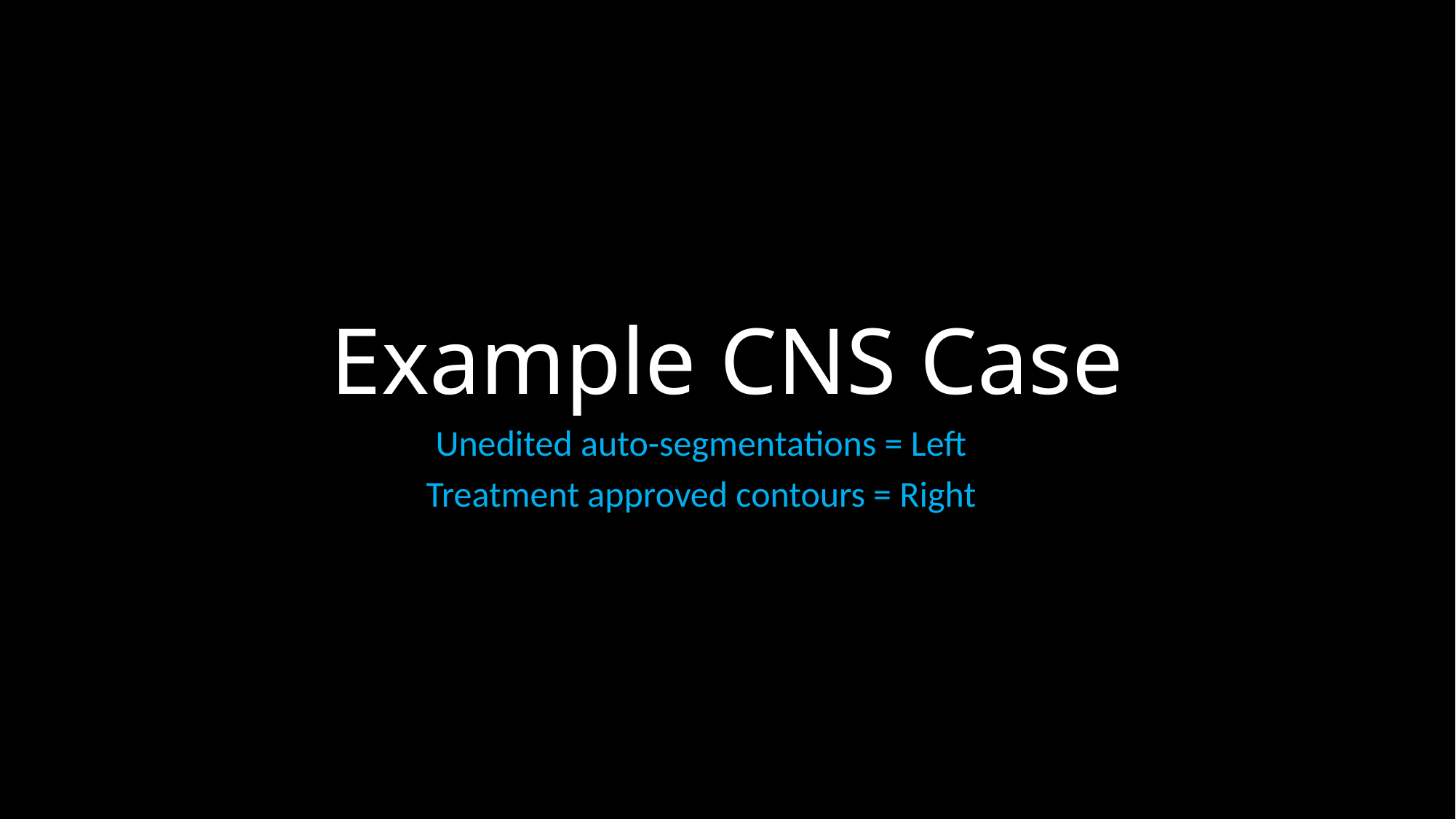

# Example CNS Case
Unedited auto-segmentations = Left
Treatment approved contours = Right

## Slide 2
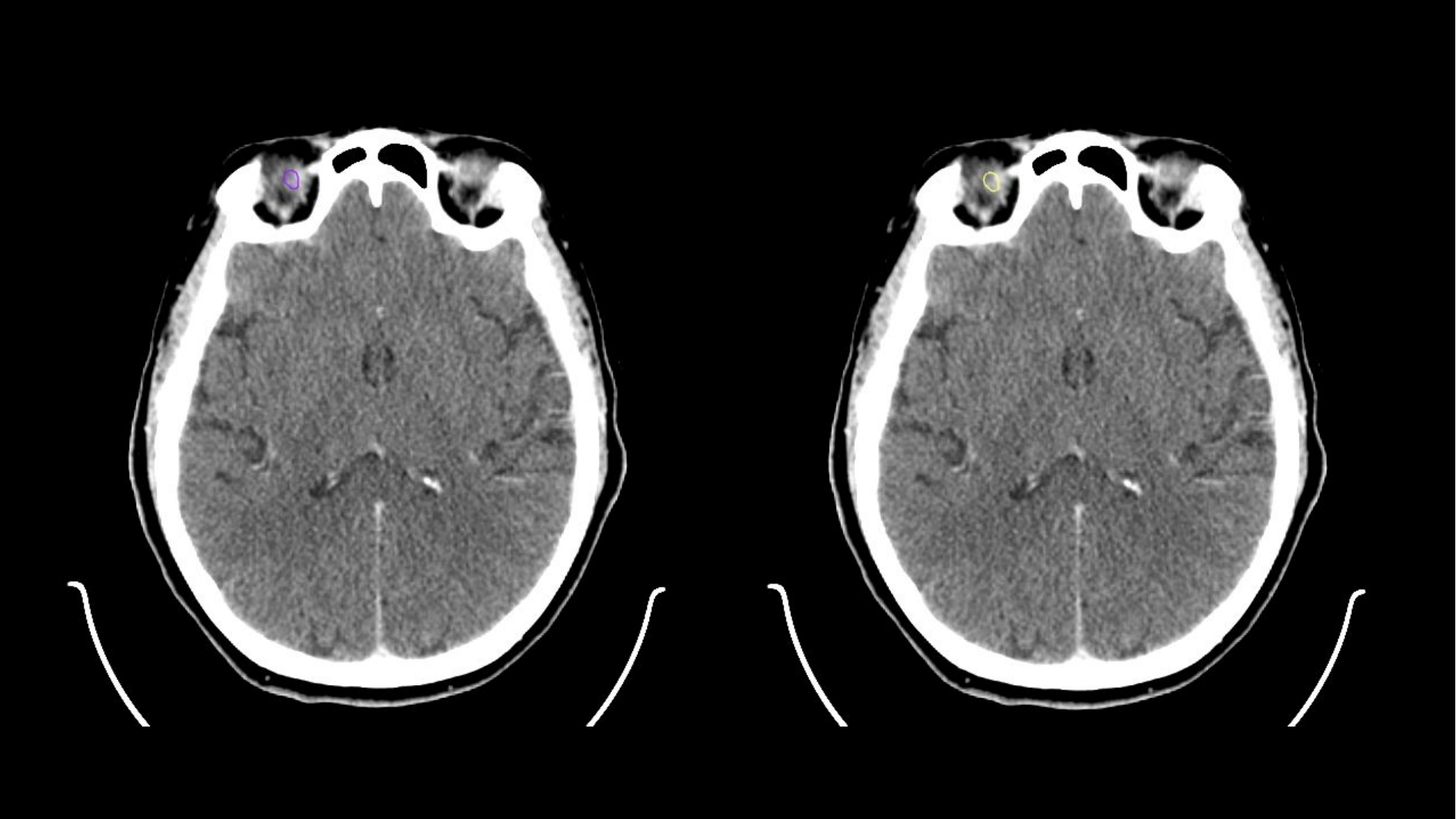

## Slide 3
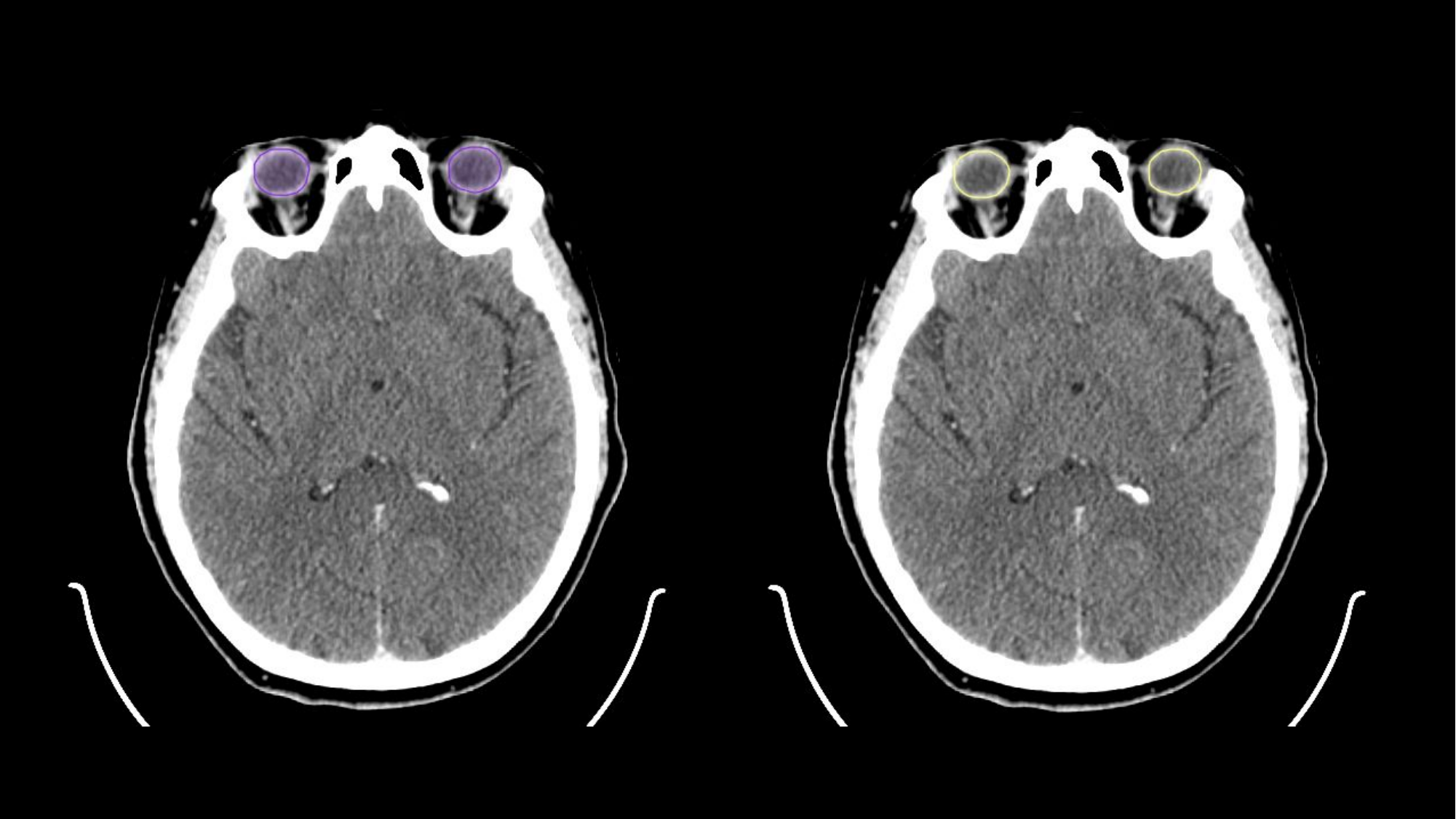

## Slide 4
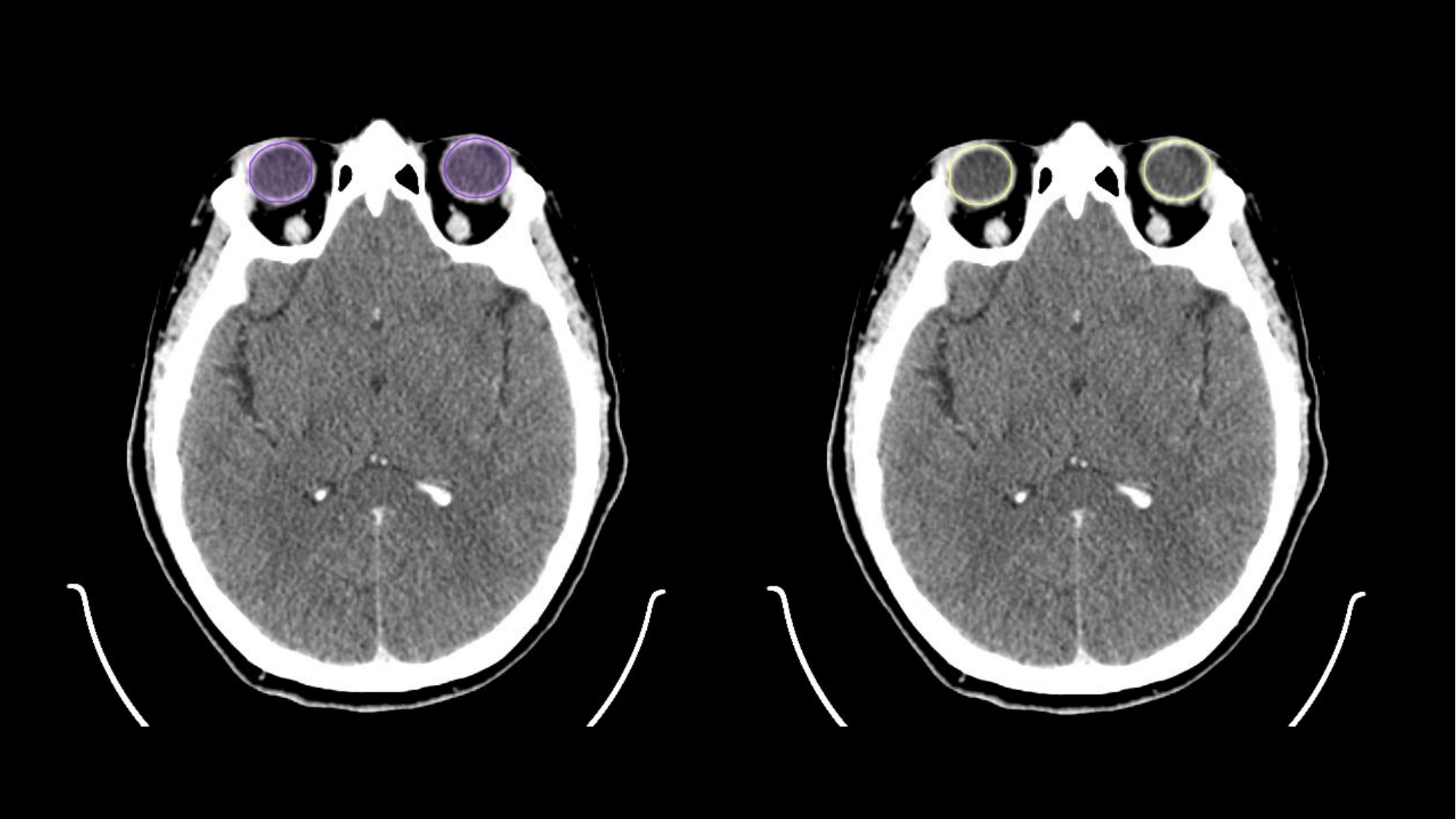

## Slide 5
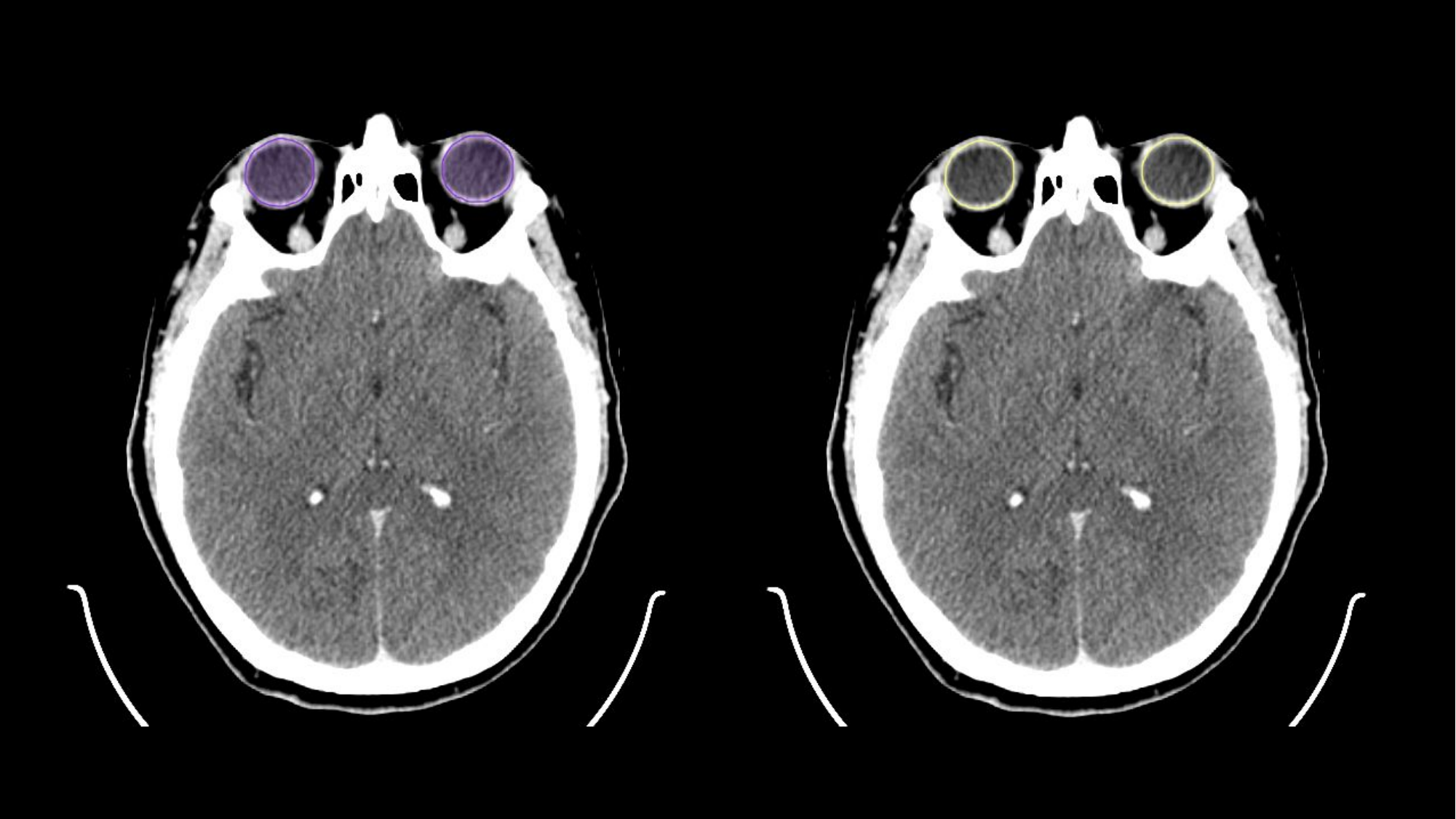

## Slide 6
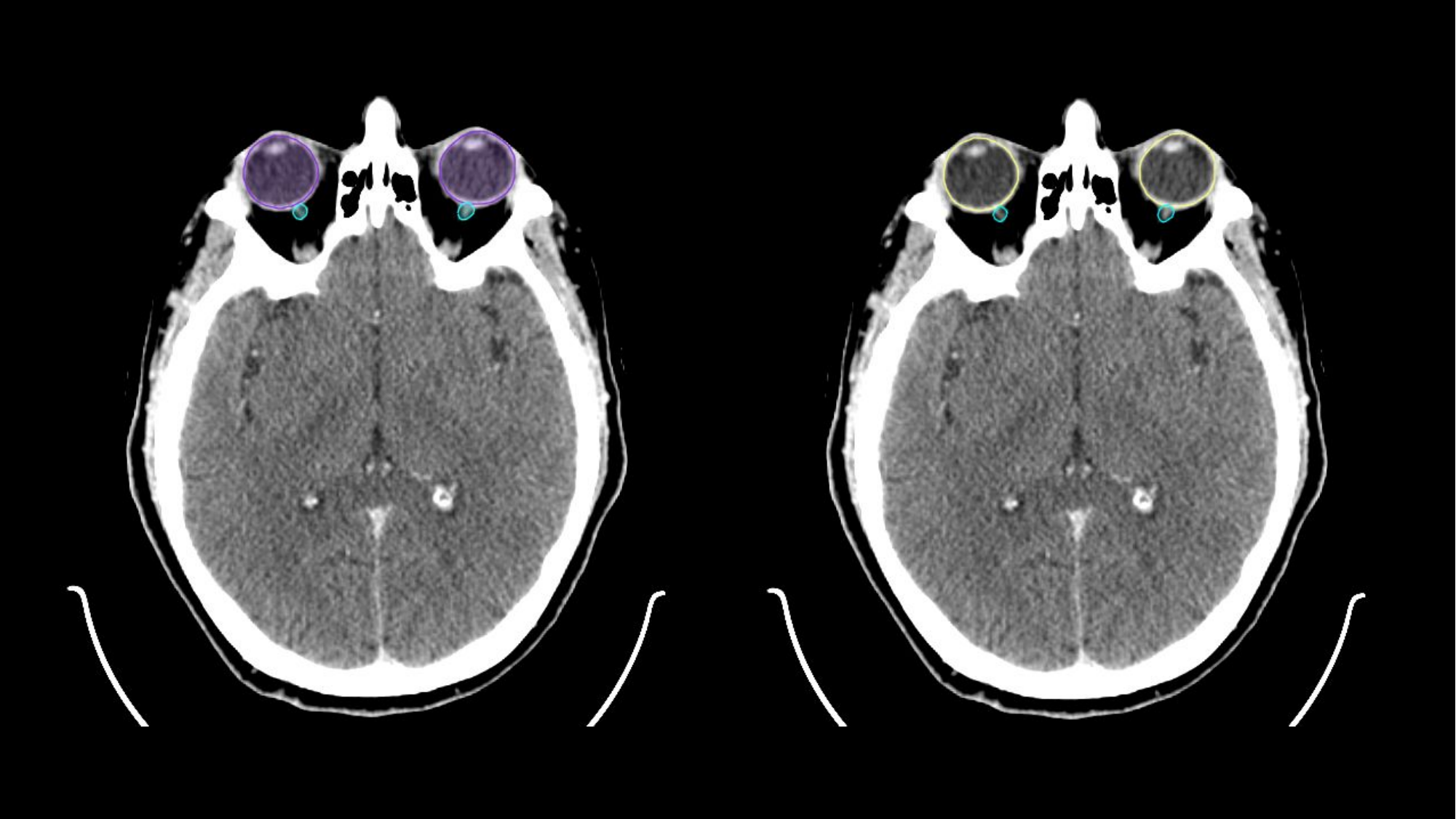

## Slide 7
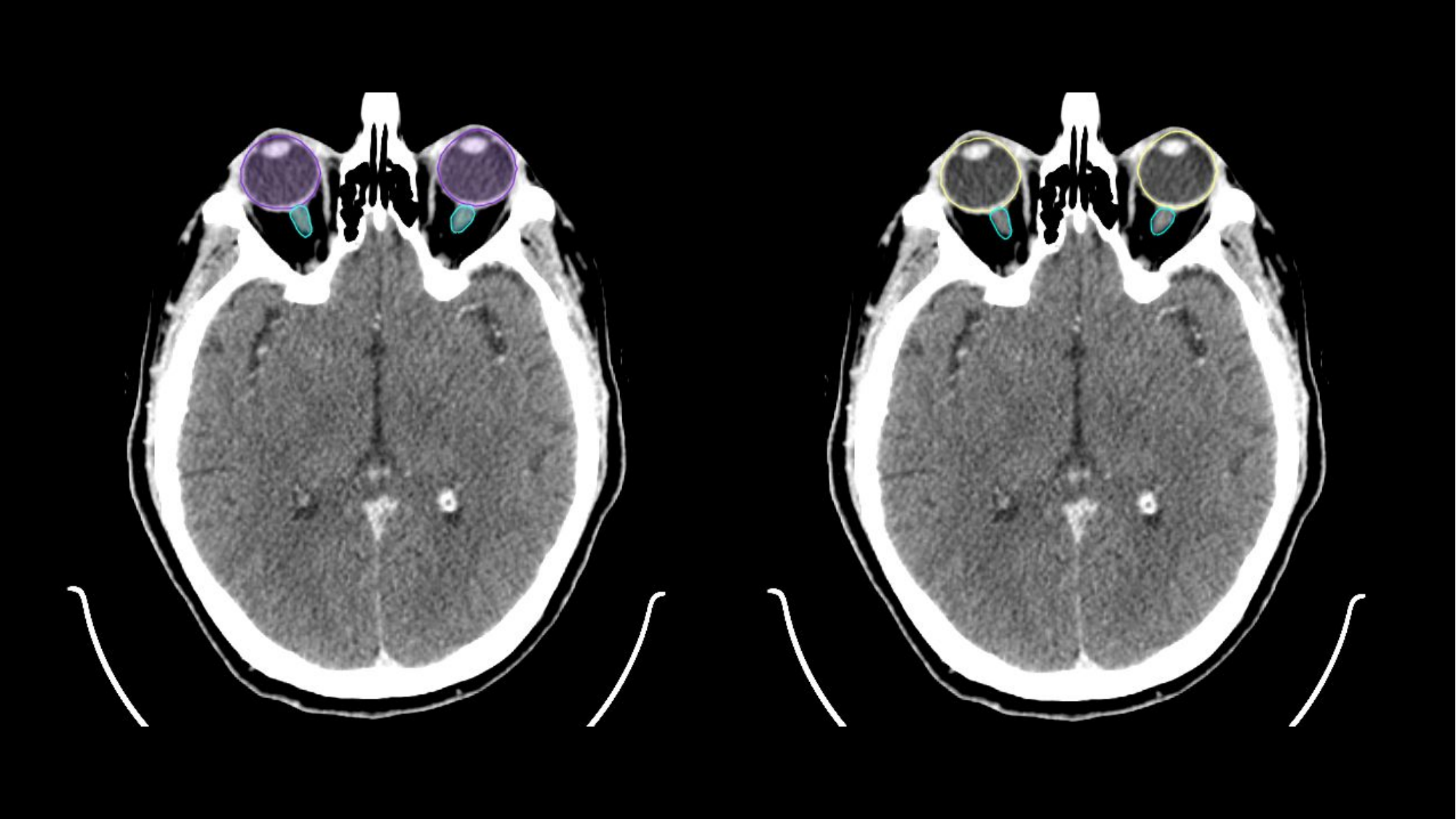

## Slide 8
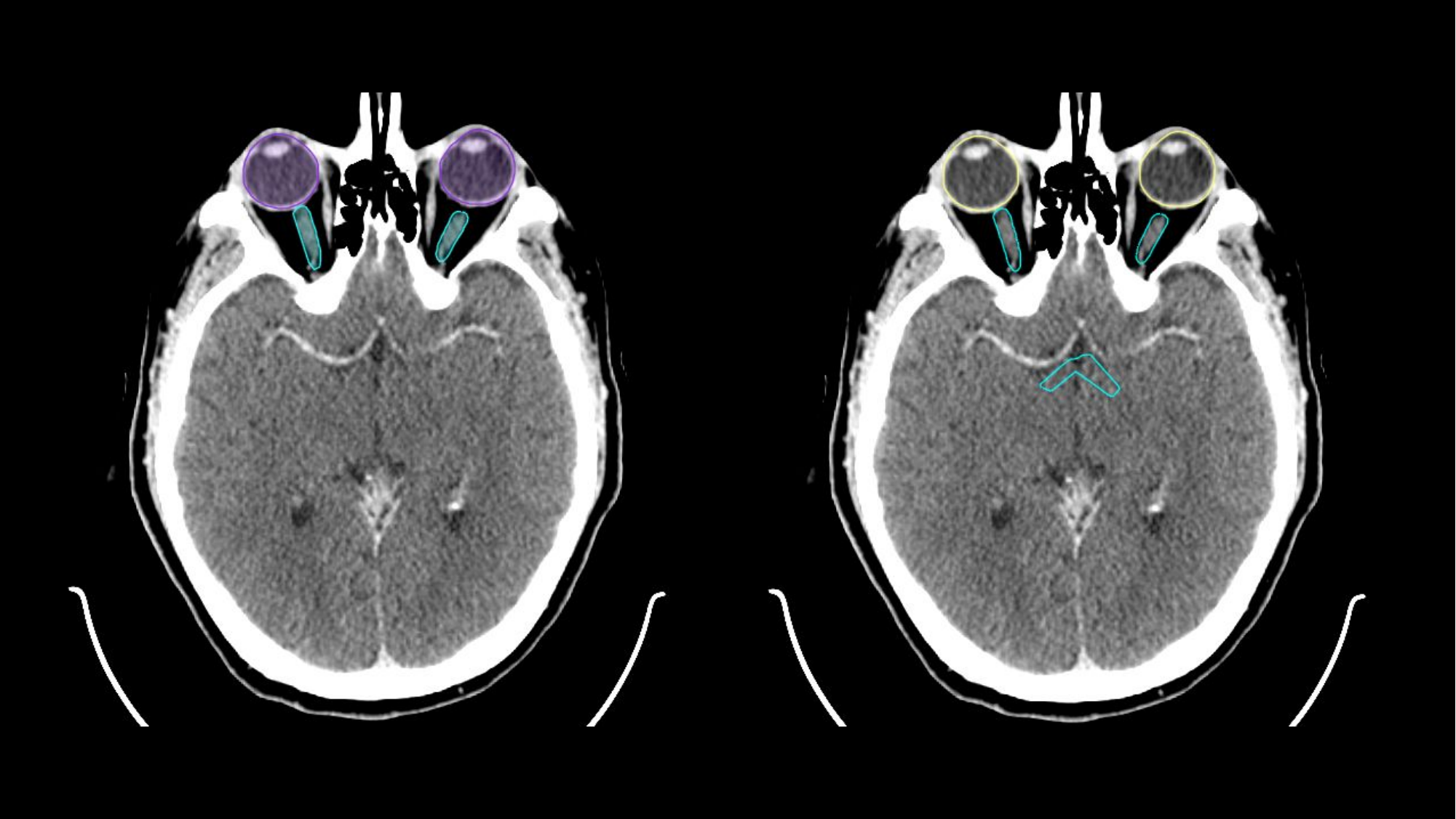

## Slide 9
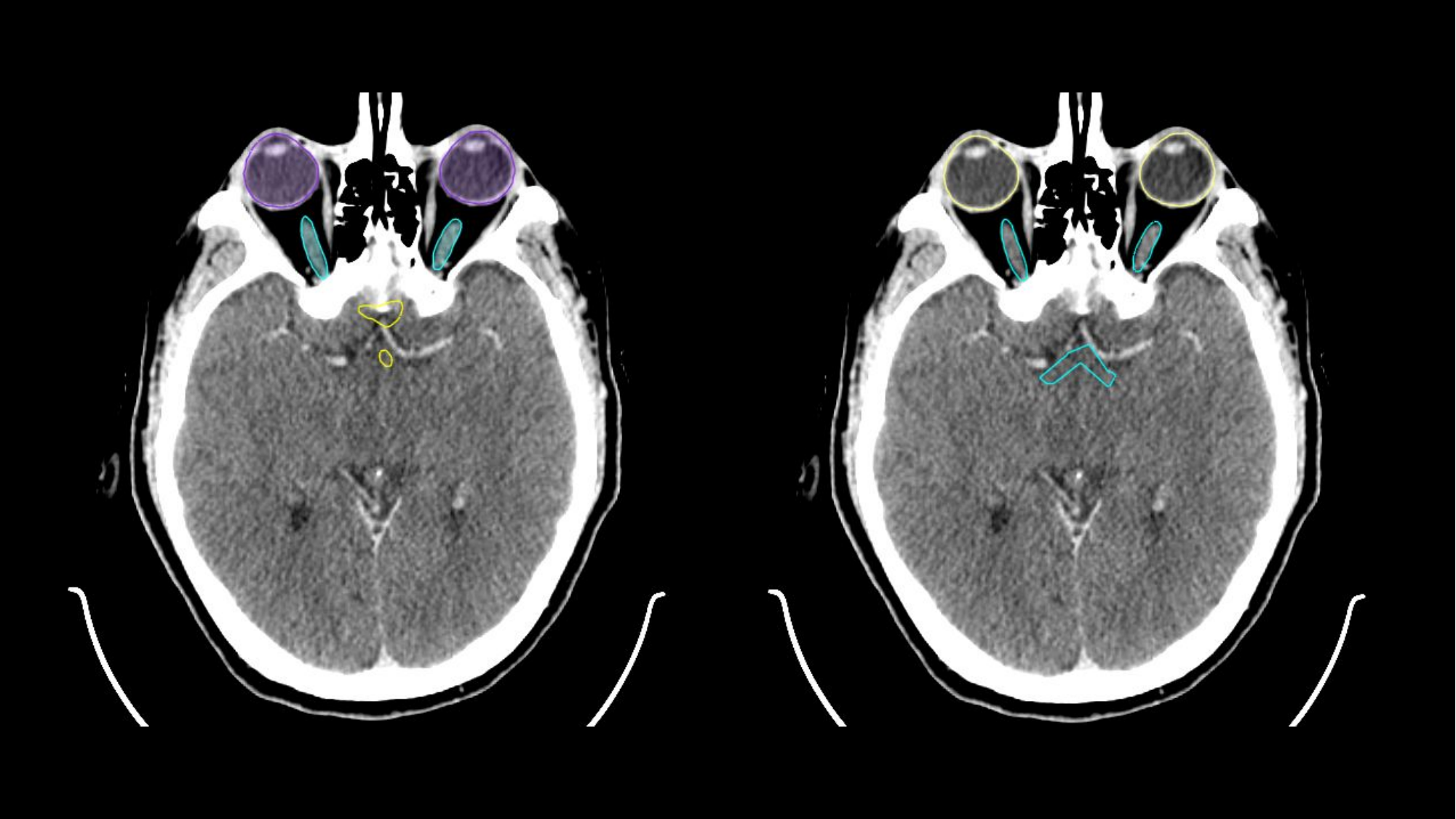

## Slide 10
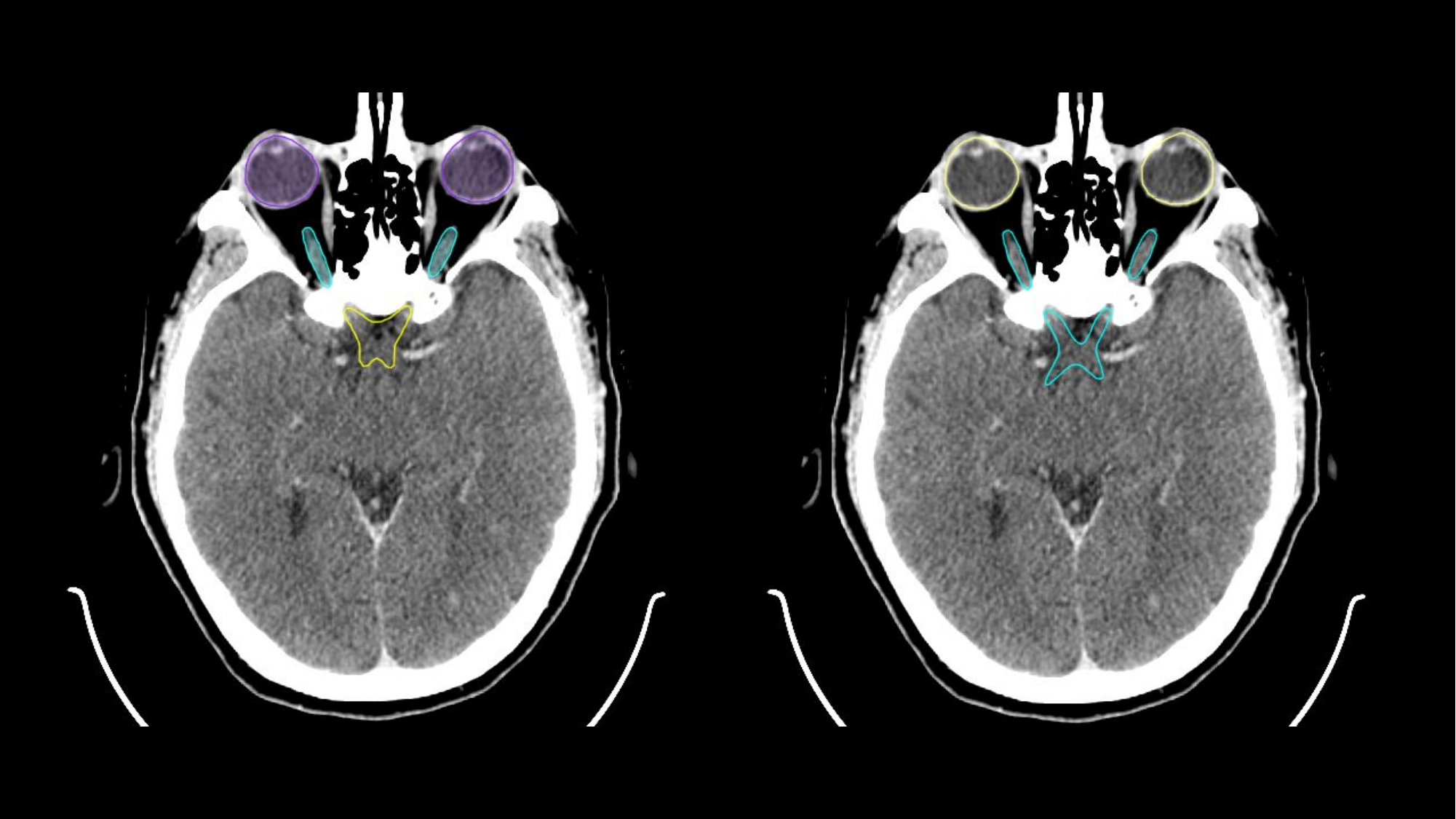

## Slide 11
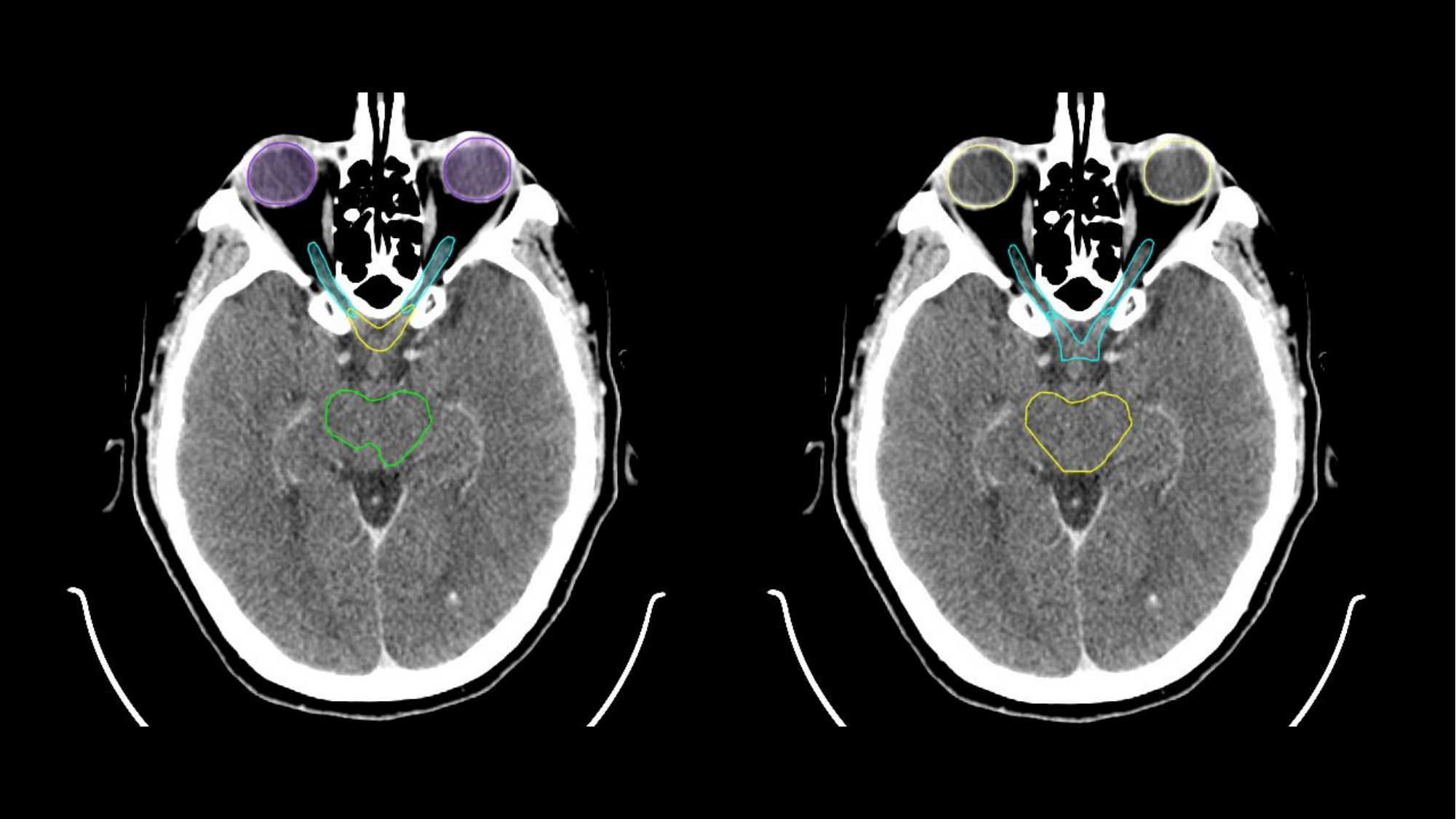

## Slide 12
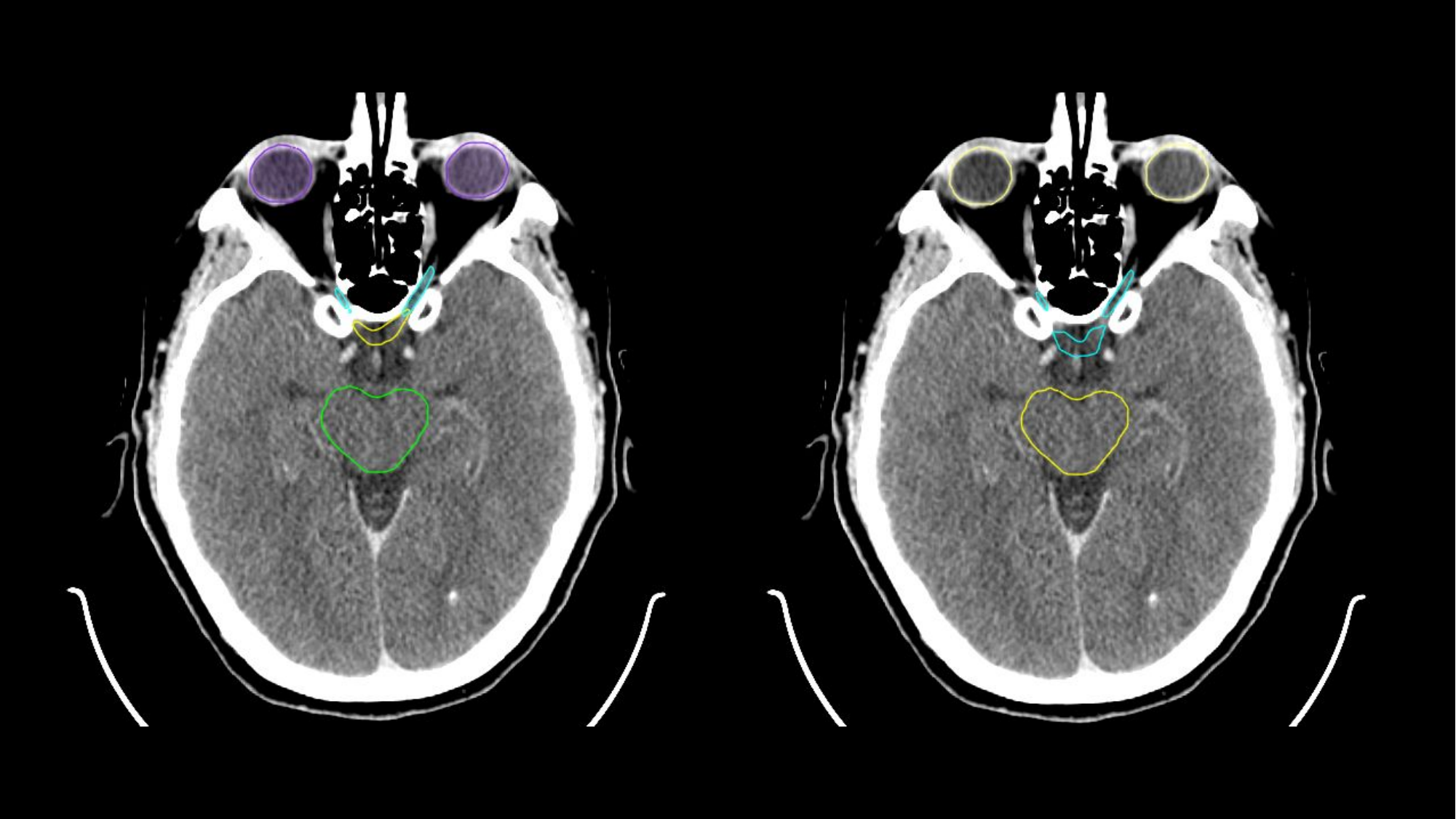

## Slide 13
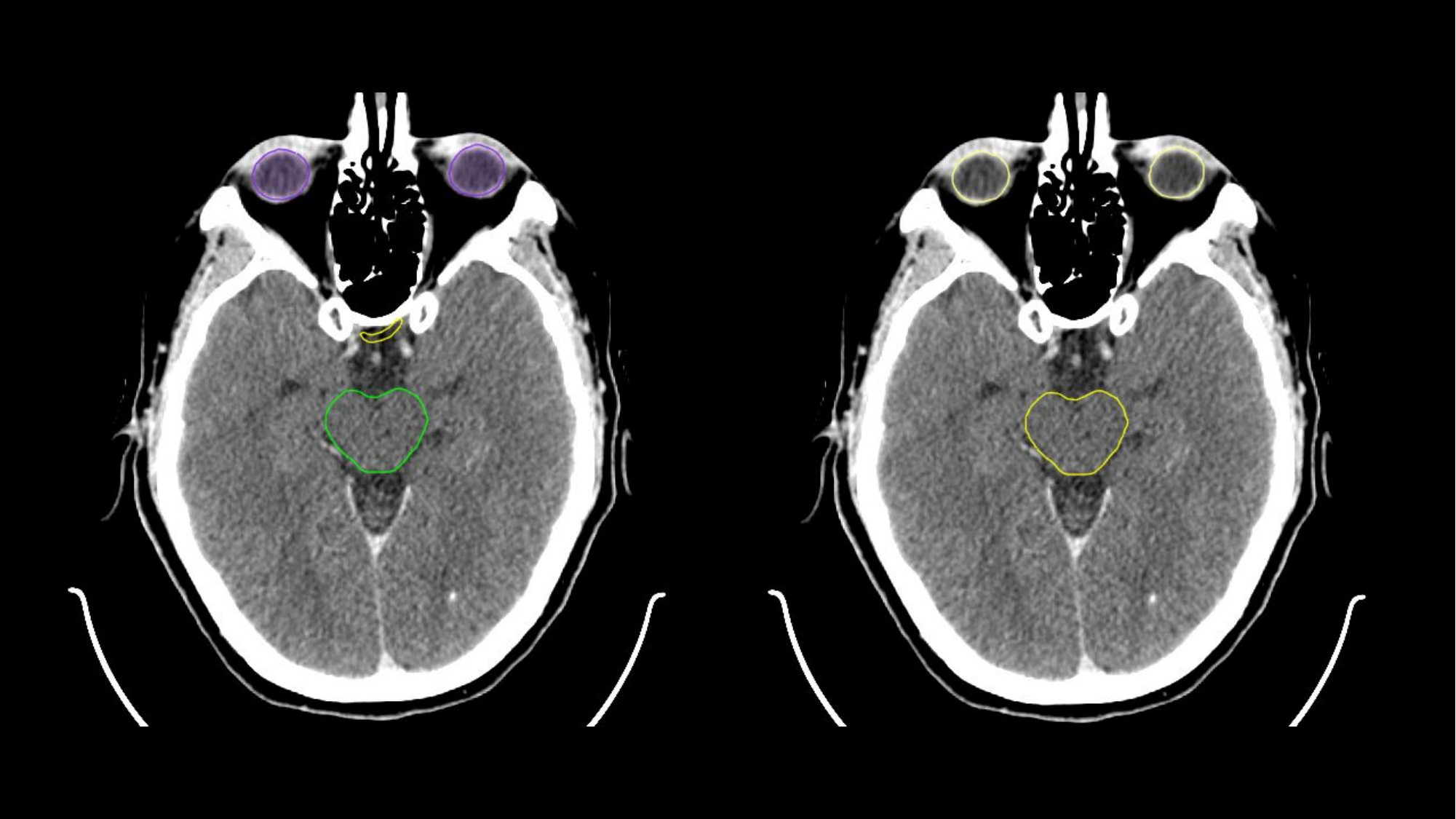

## Slide 14
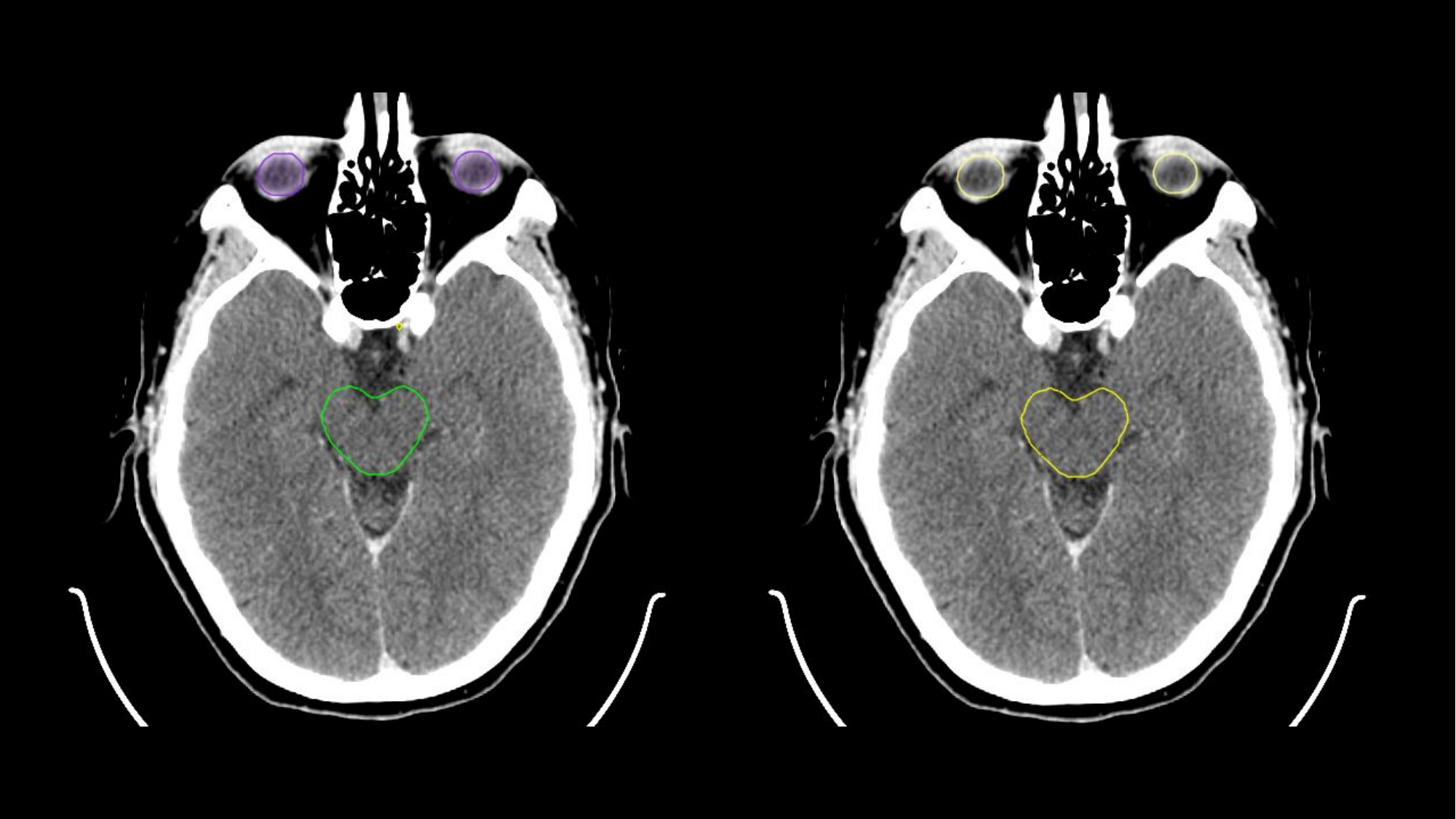

## Slide 15
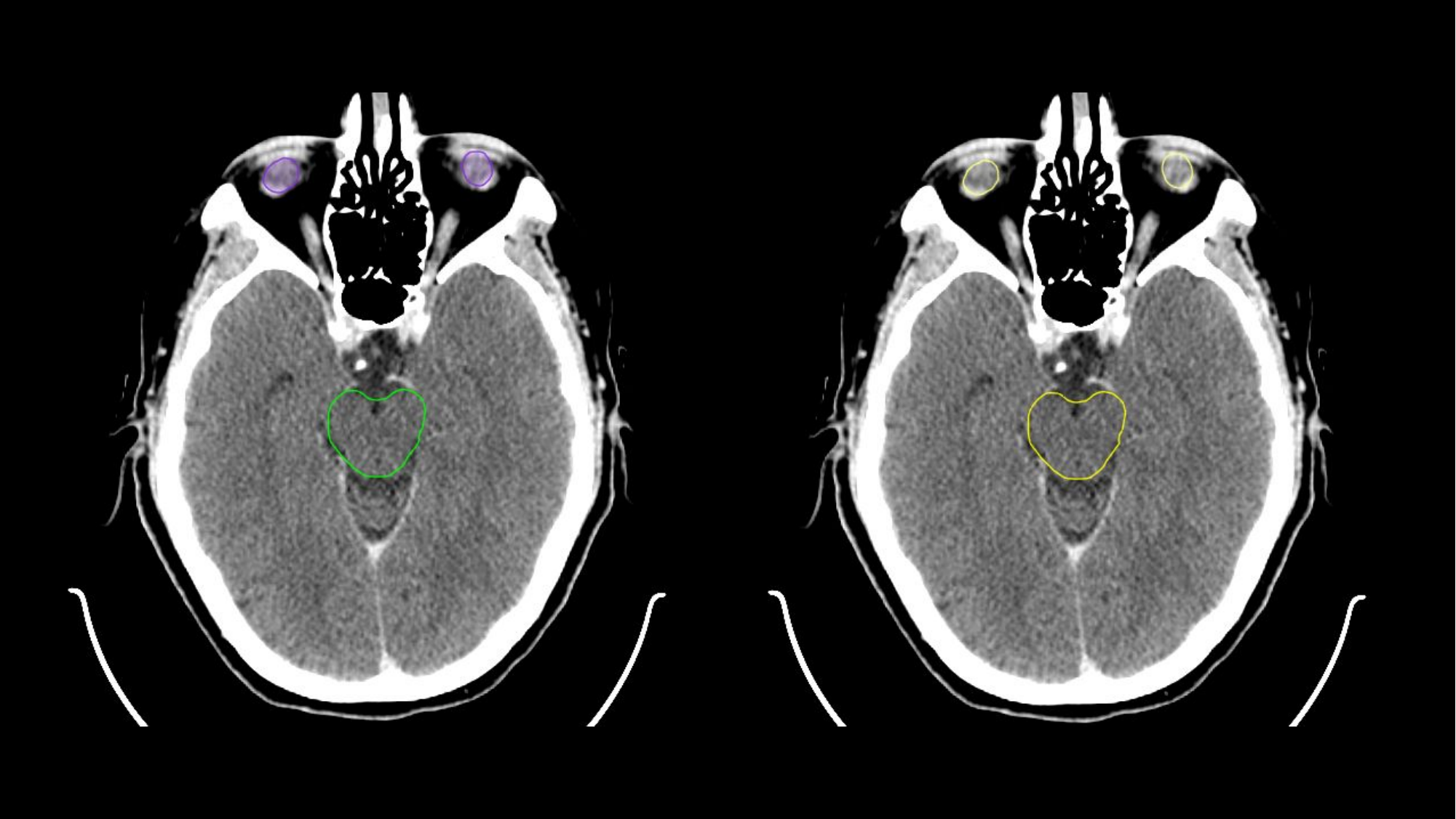

## Slide 16
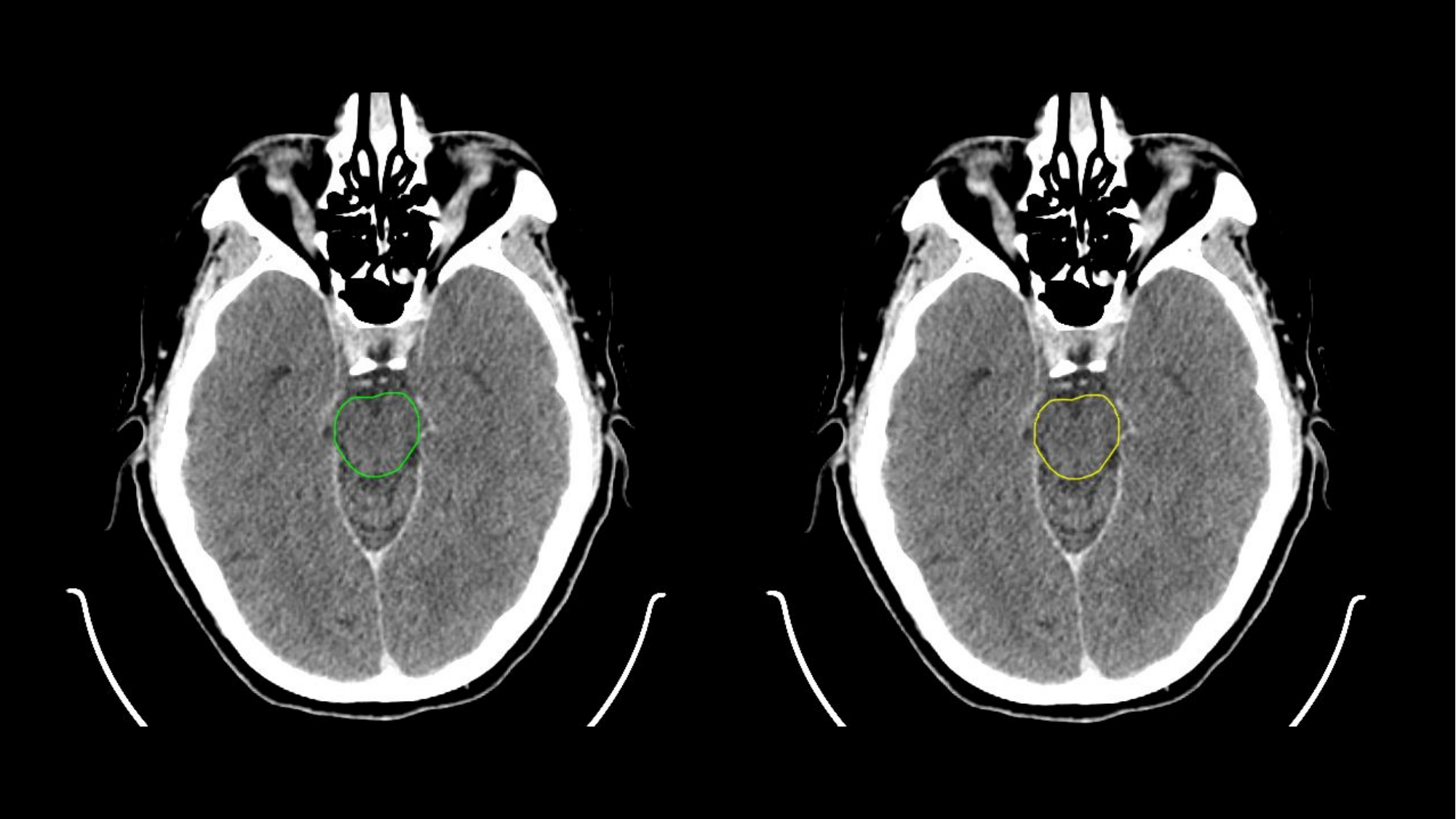

## Slide 17
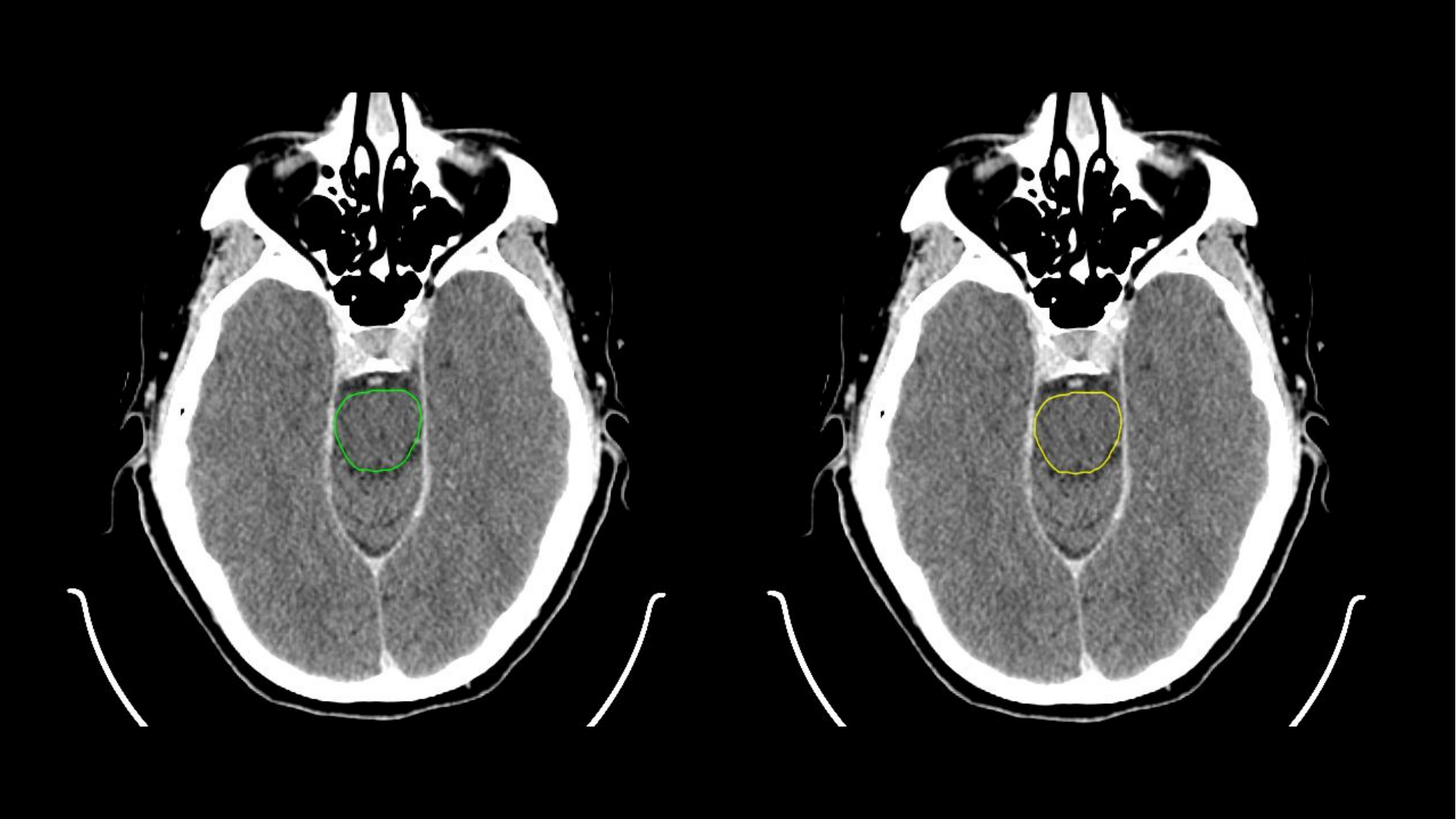

## Slide 18
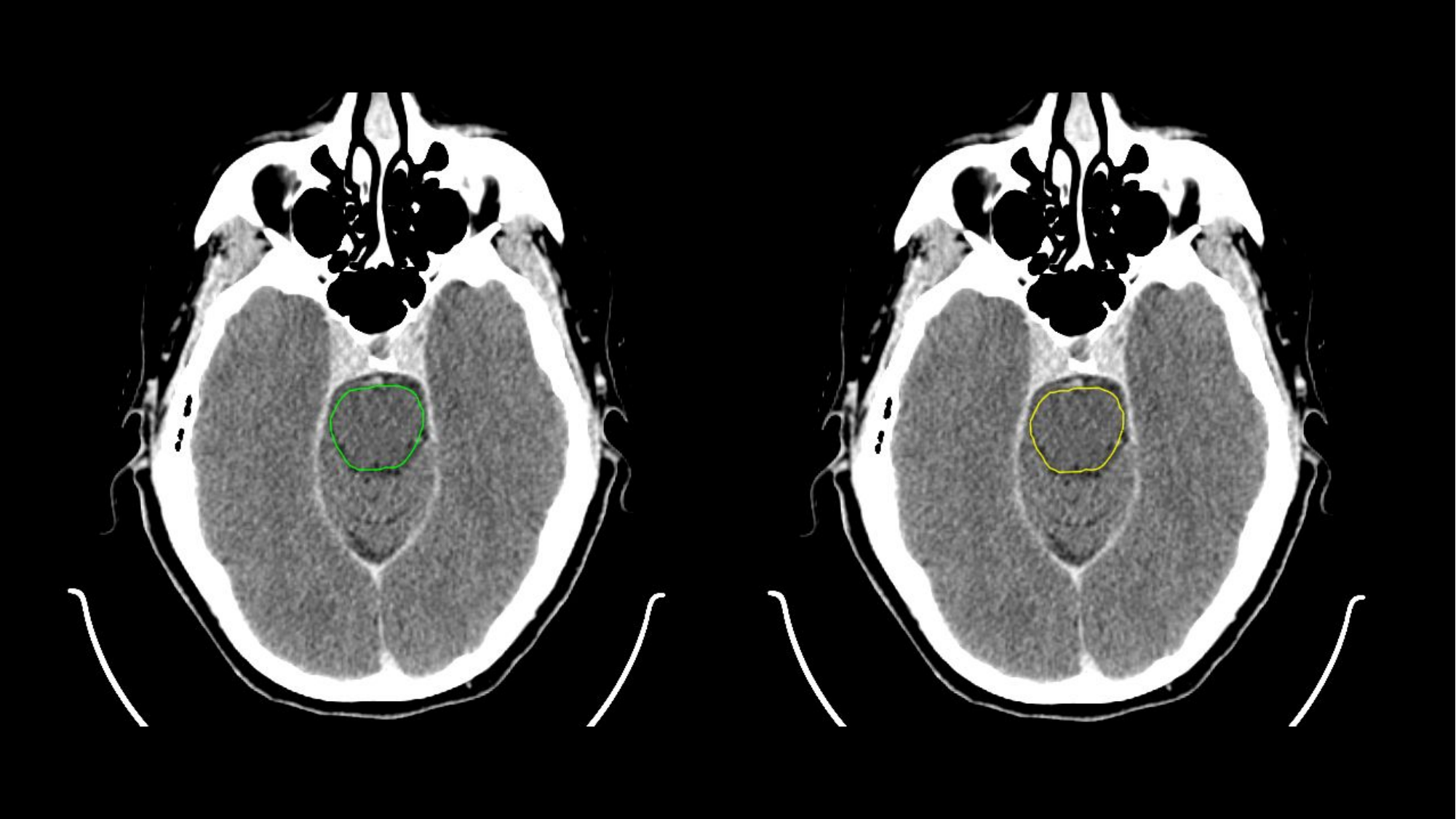

## Slide 19
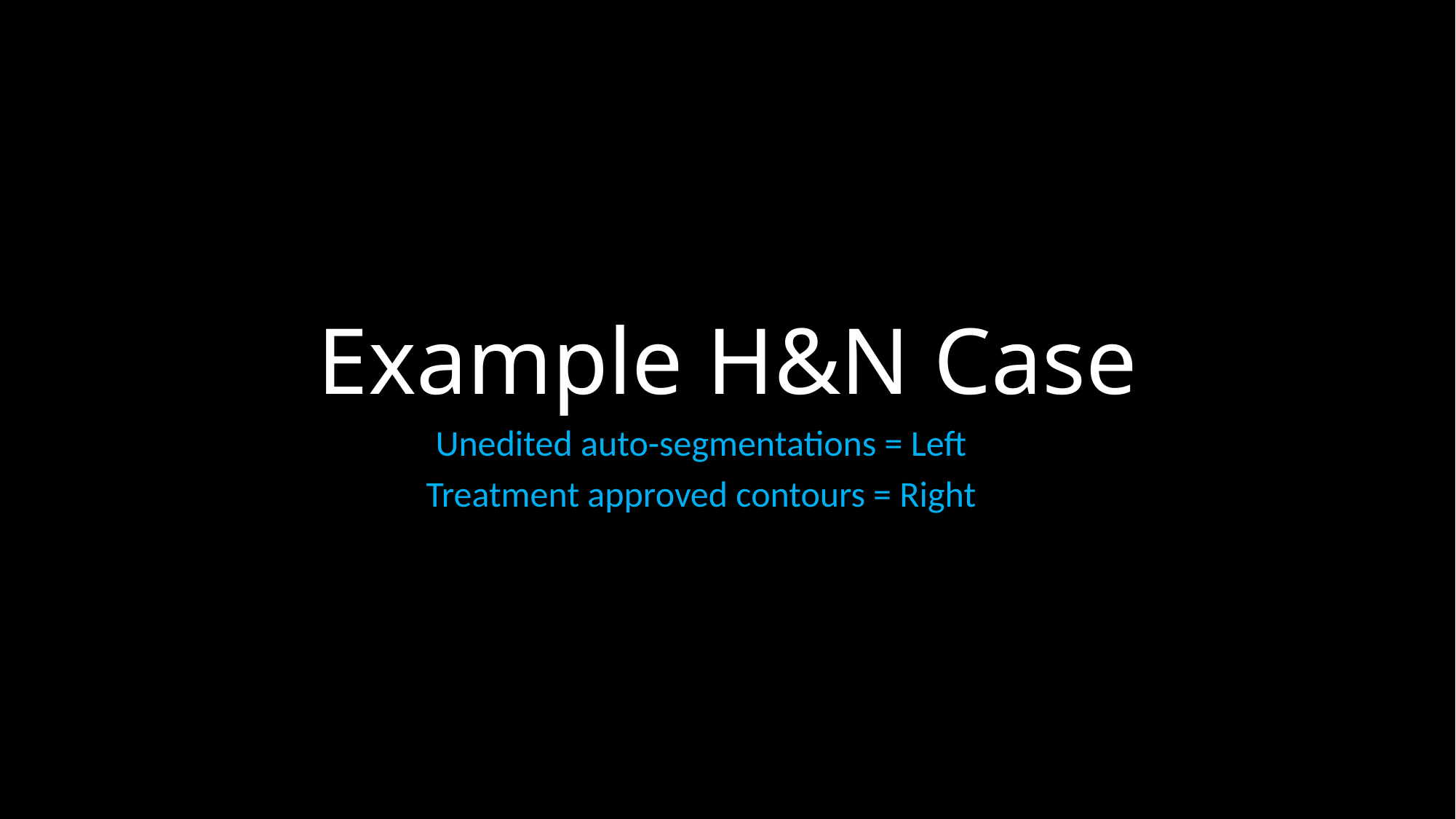

# Example H&N Case
Unedited auto-segmentations = Left
Treatment approved contours = Right

## Slide 20
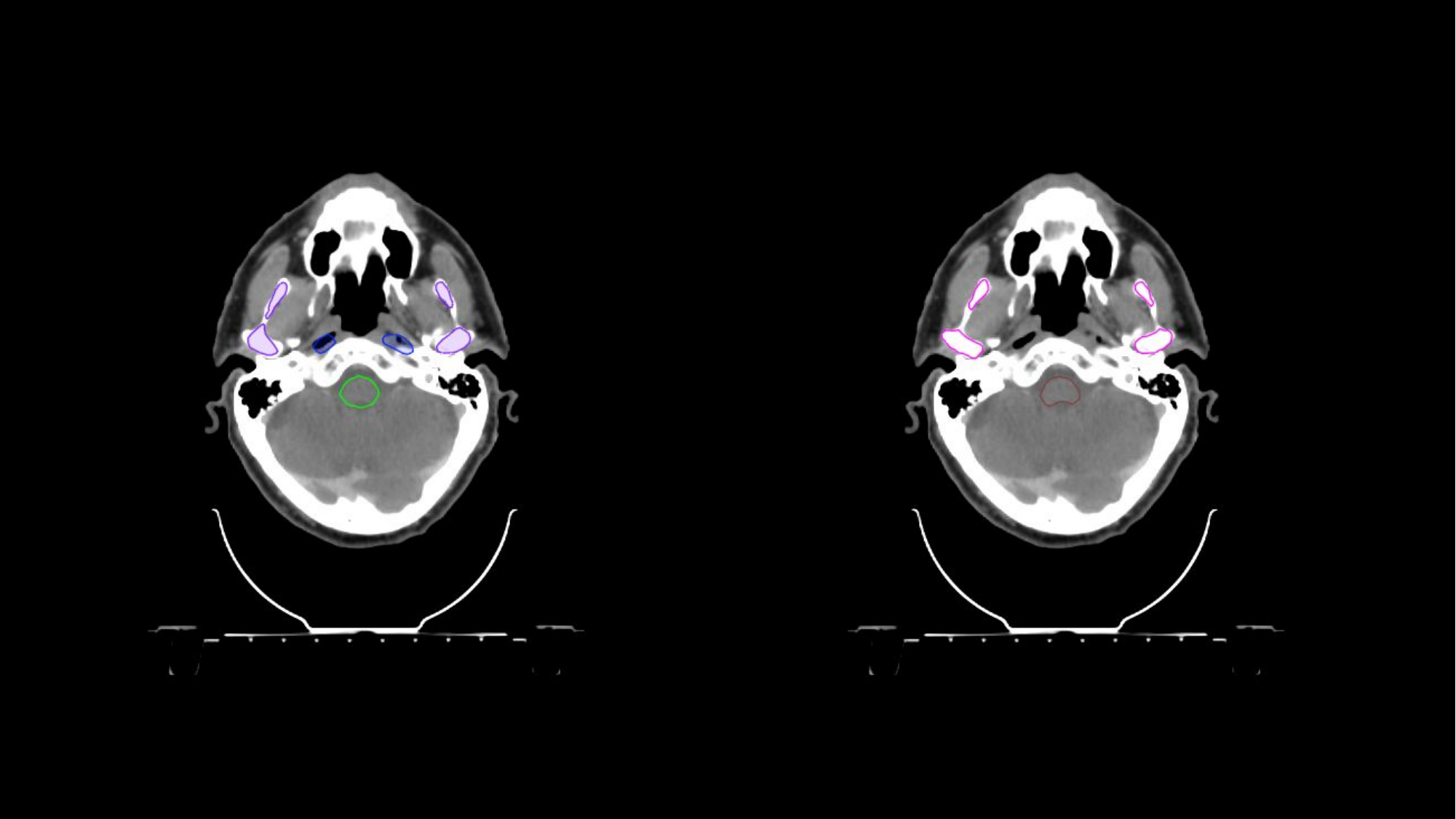

## Slide 21
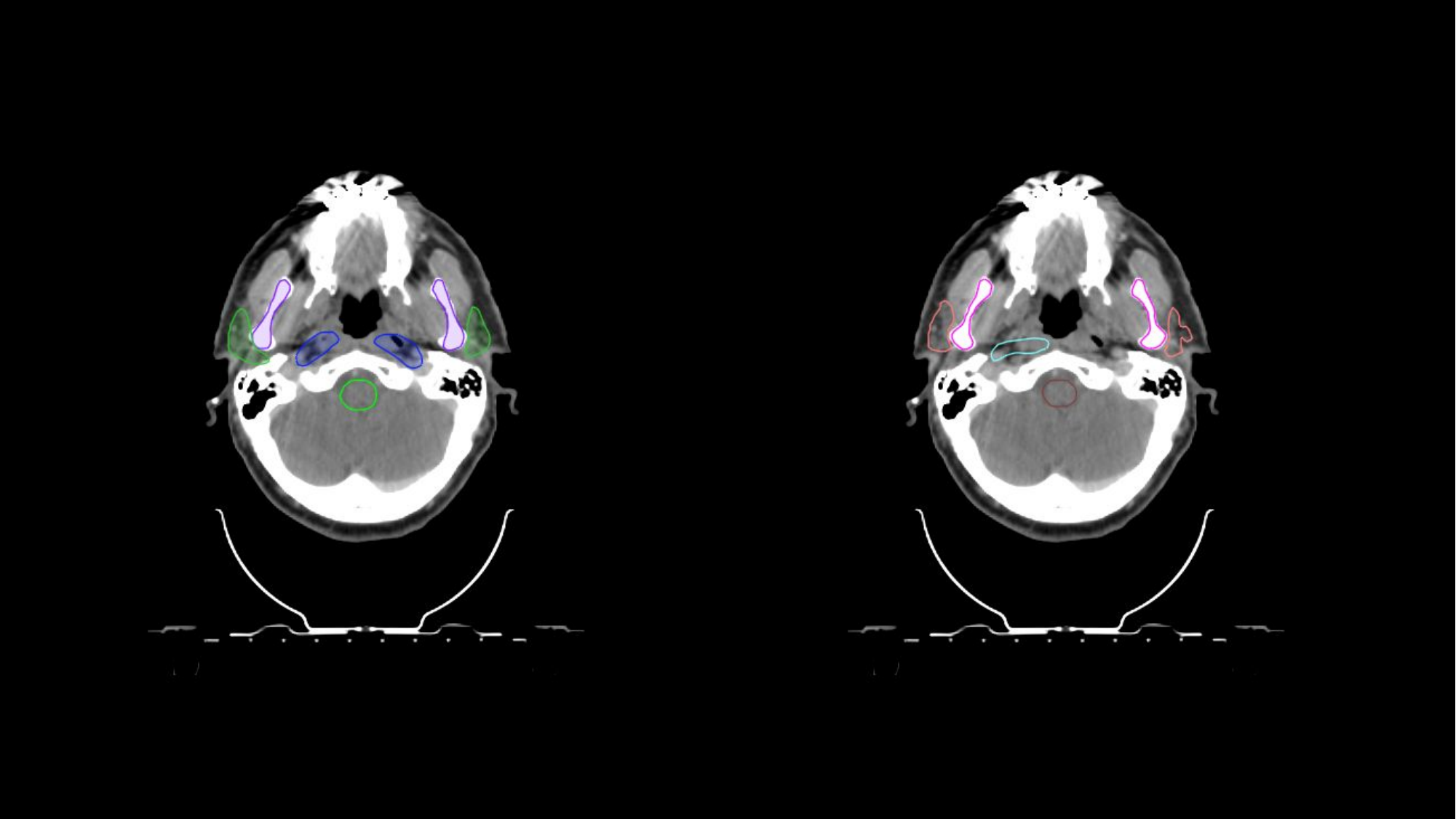

## Slide 22
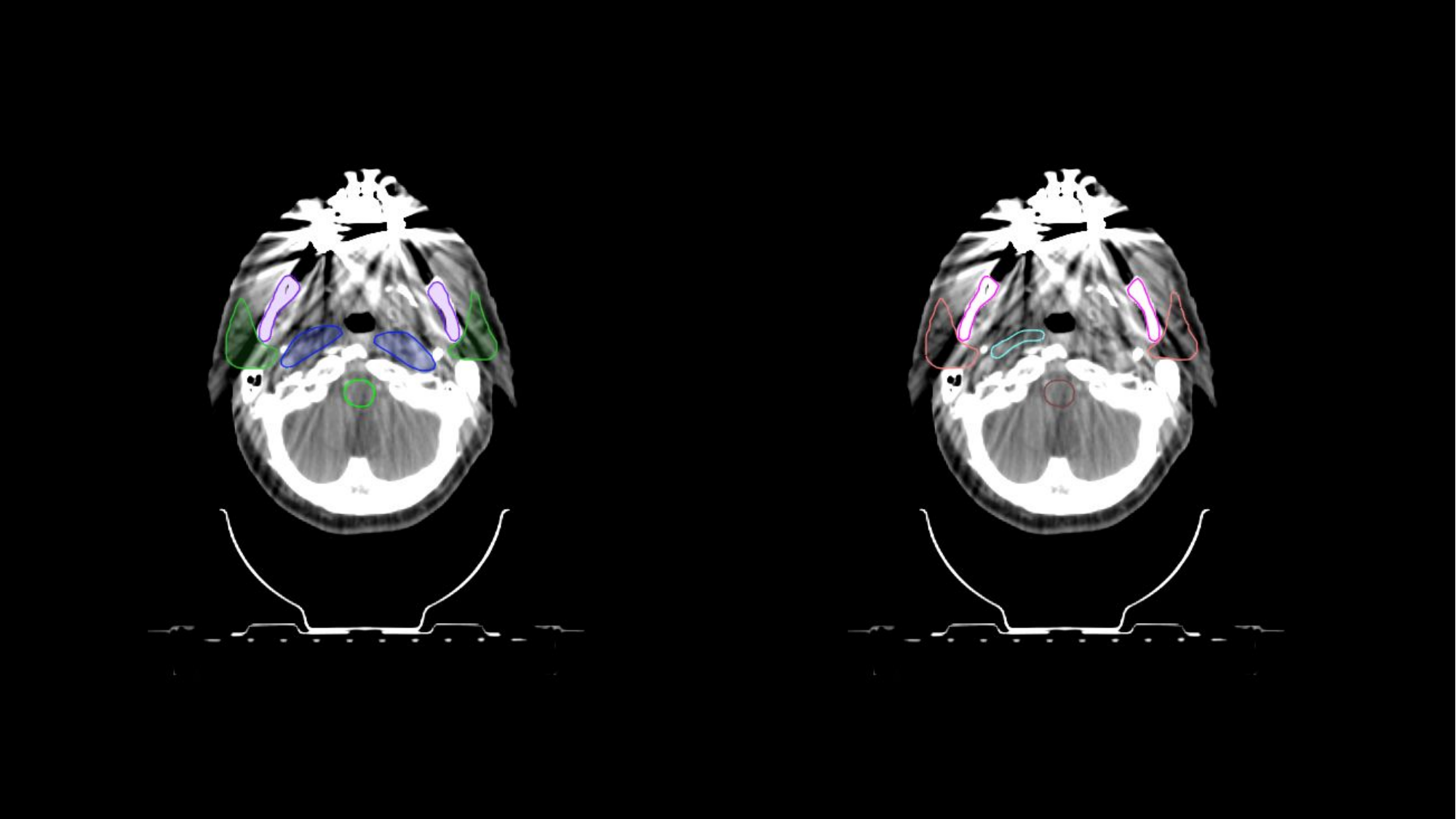

## Slide 23
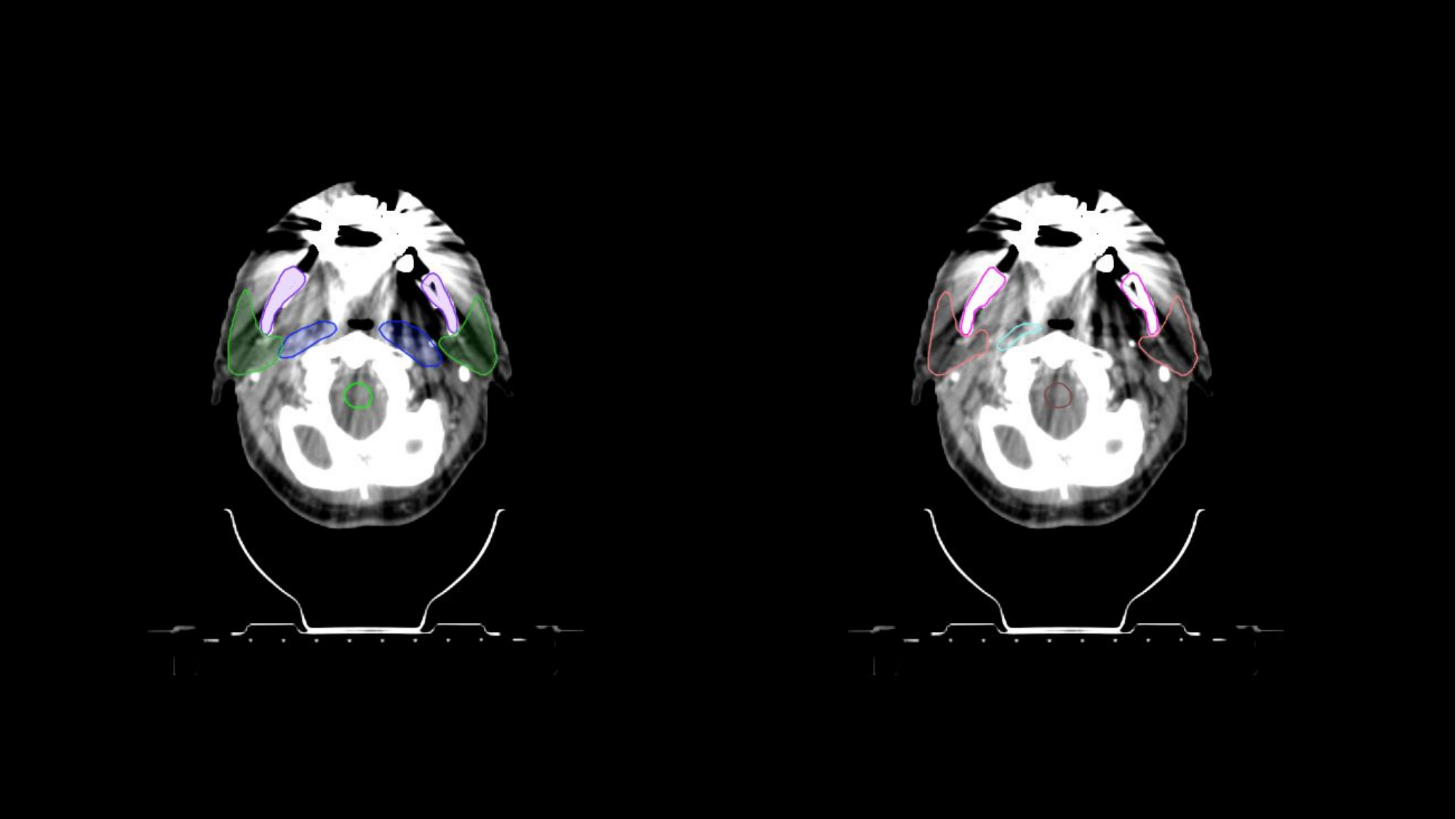

## Slide 24
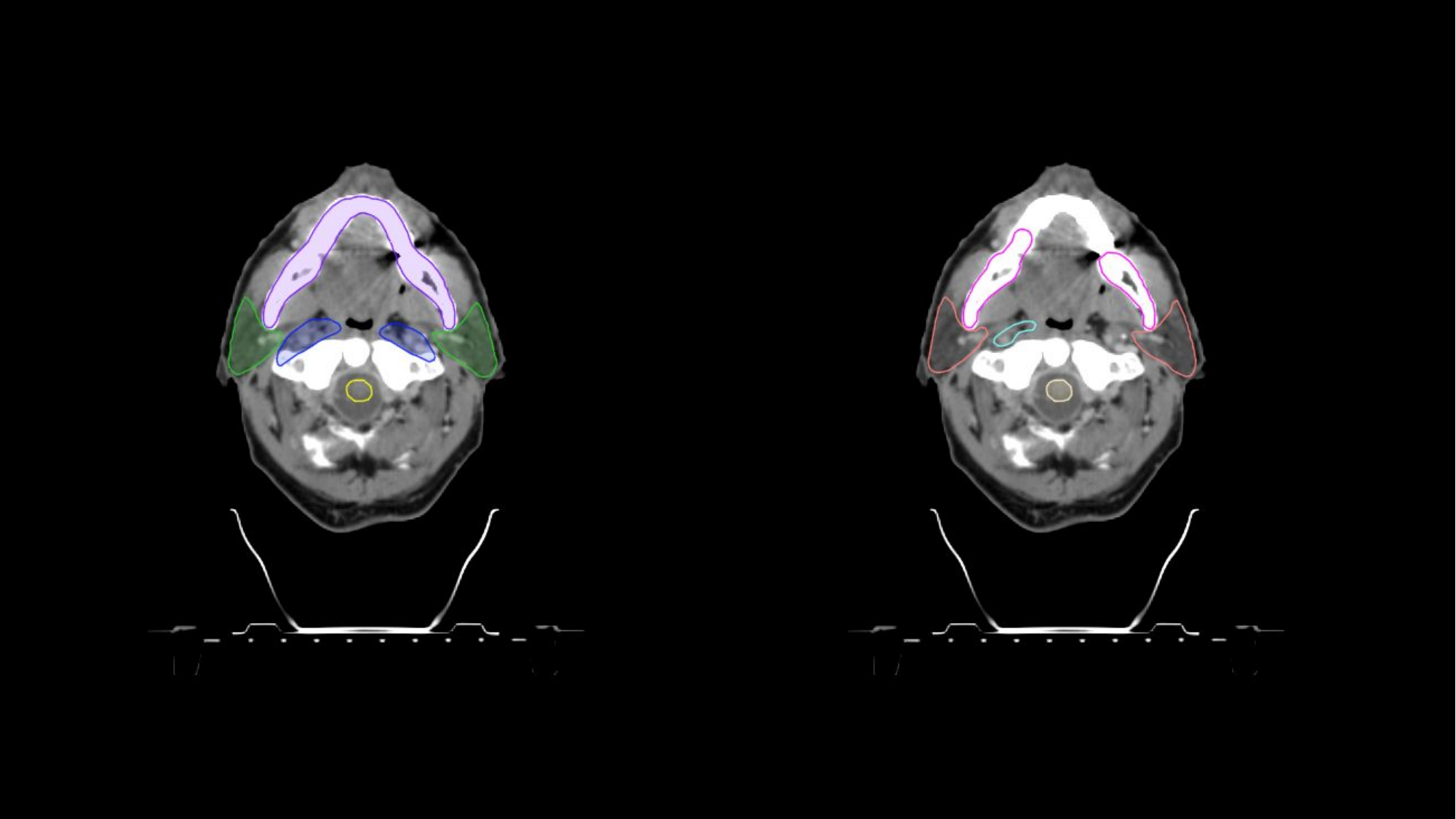

## Slide 25
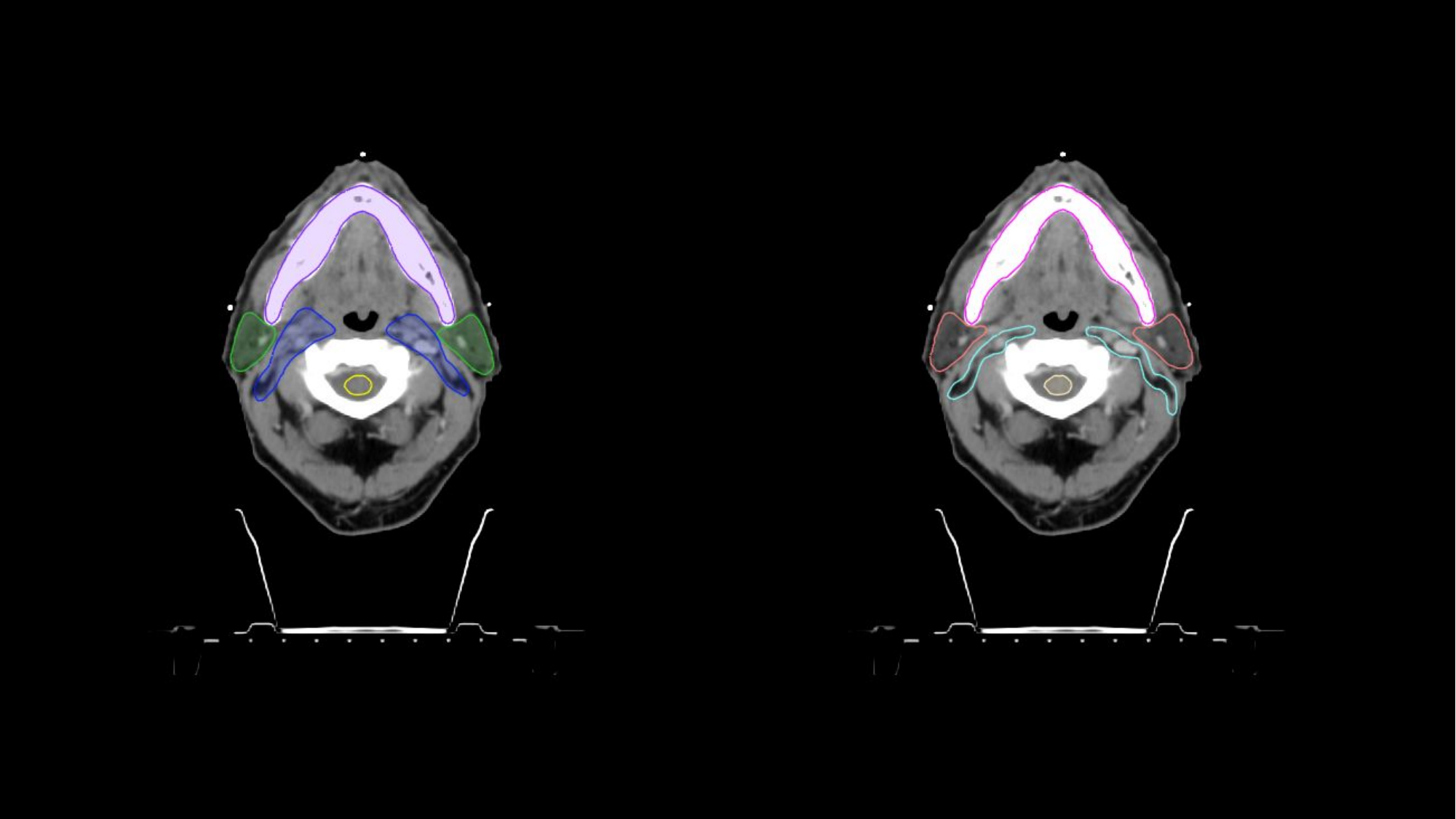

## Slide 26
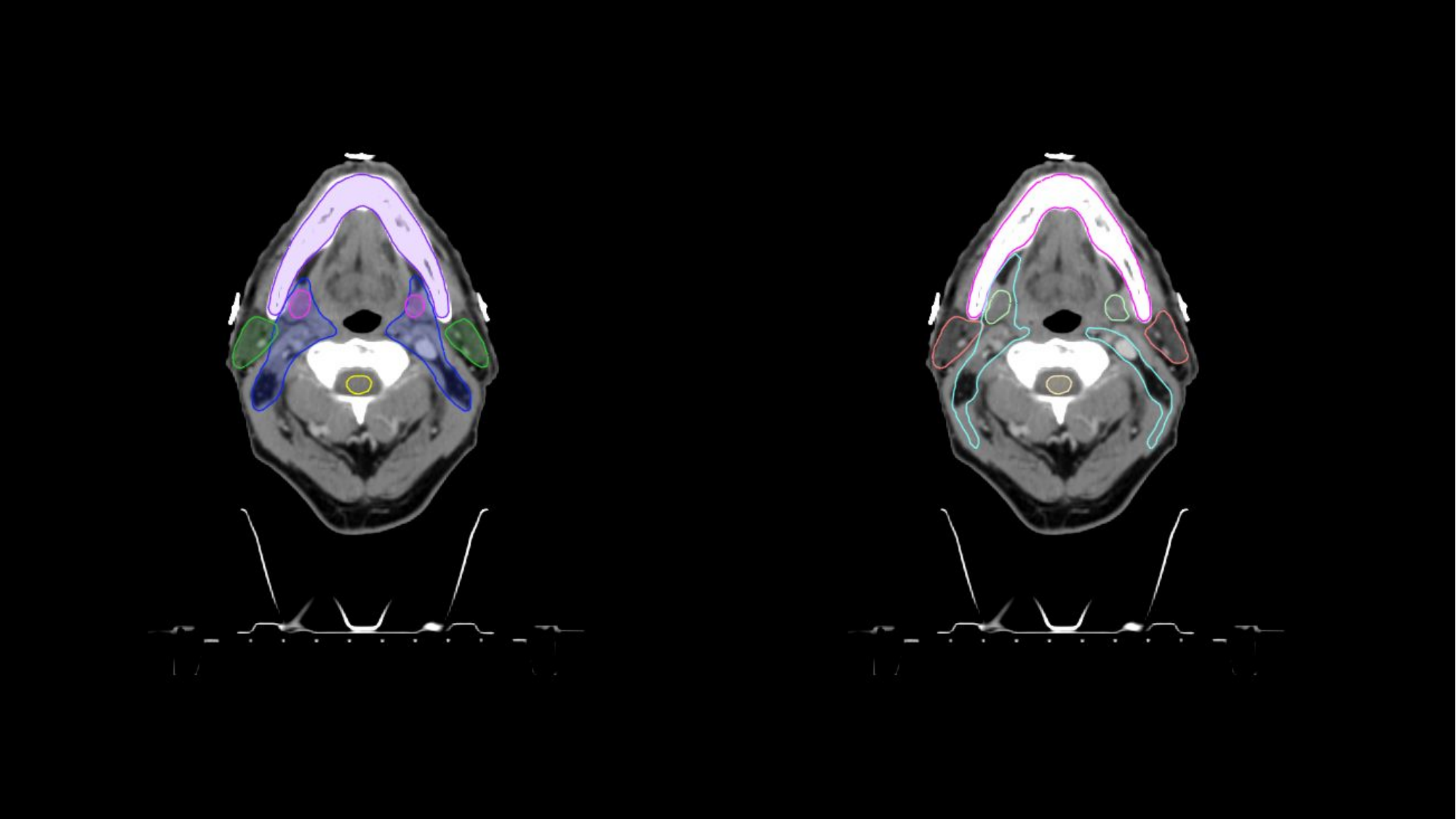

## Slide 27
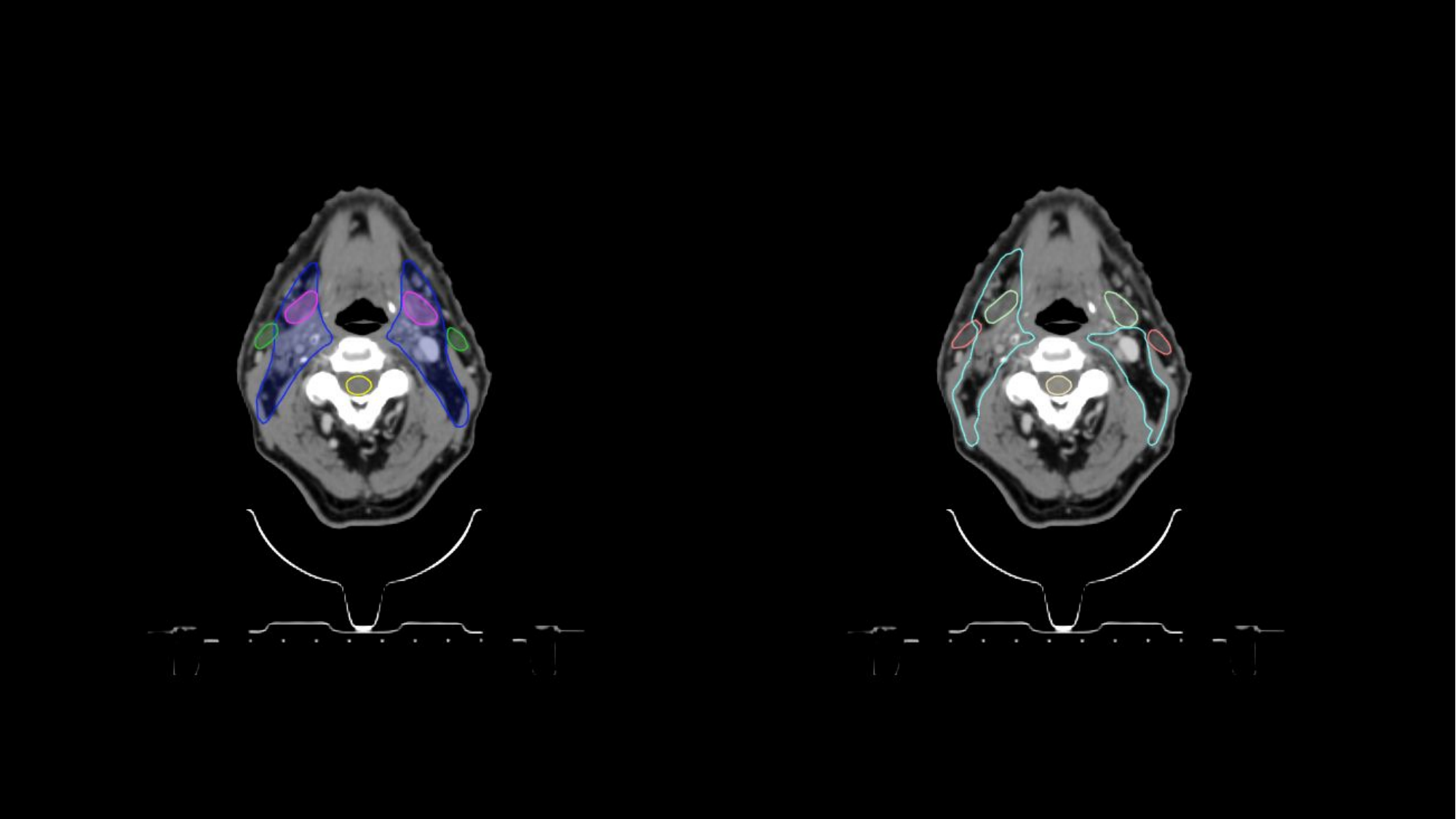

## Slide 28
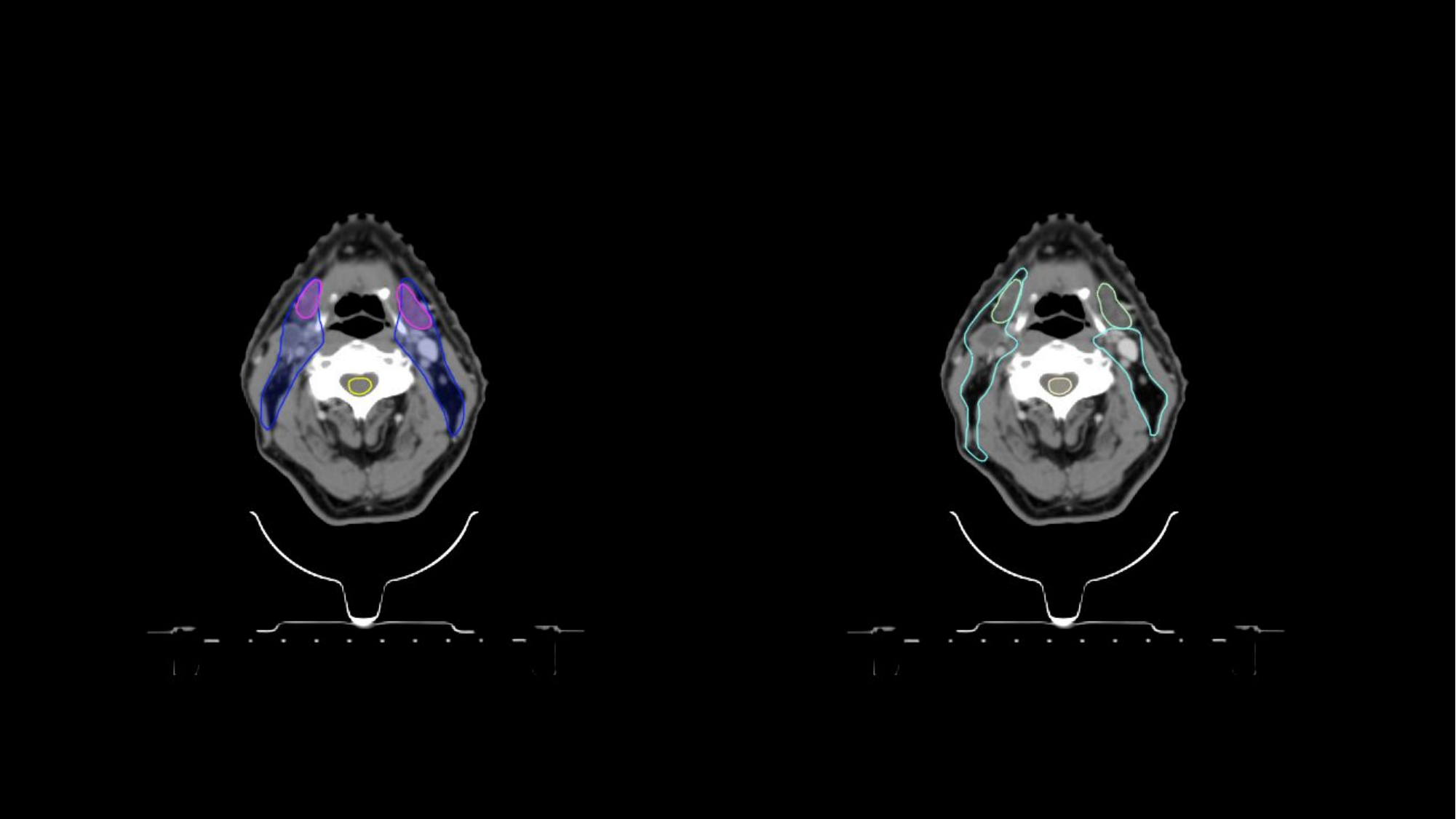

## Slide 29
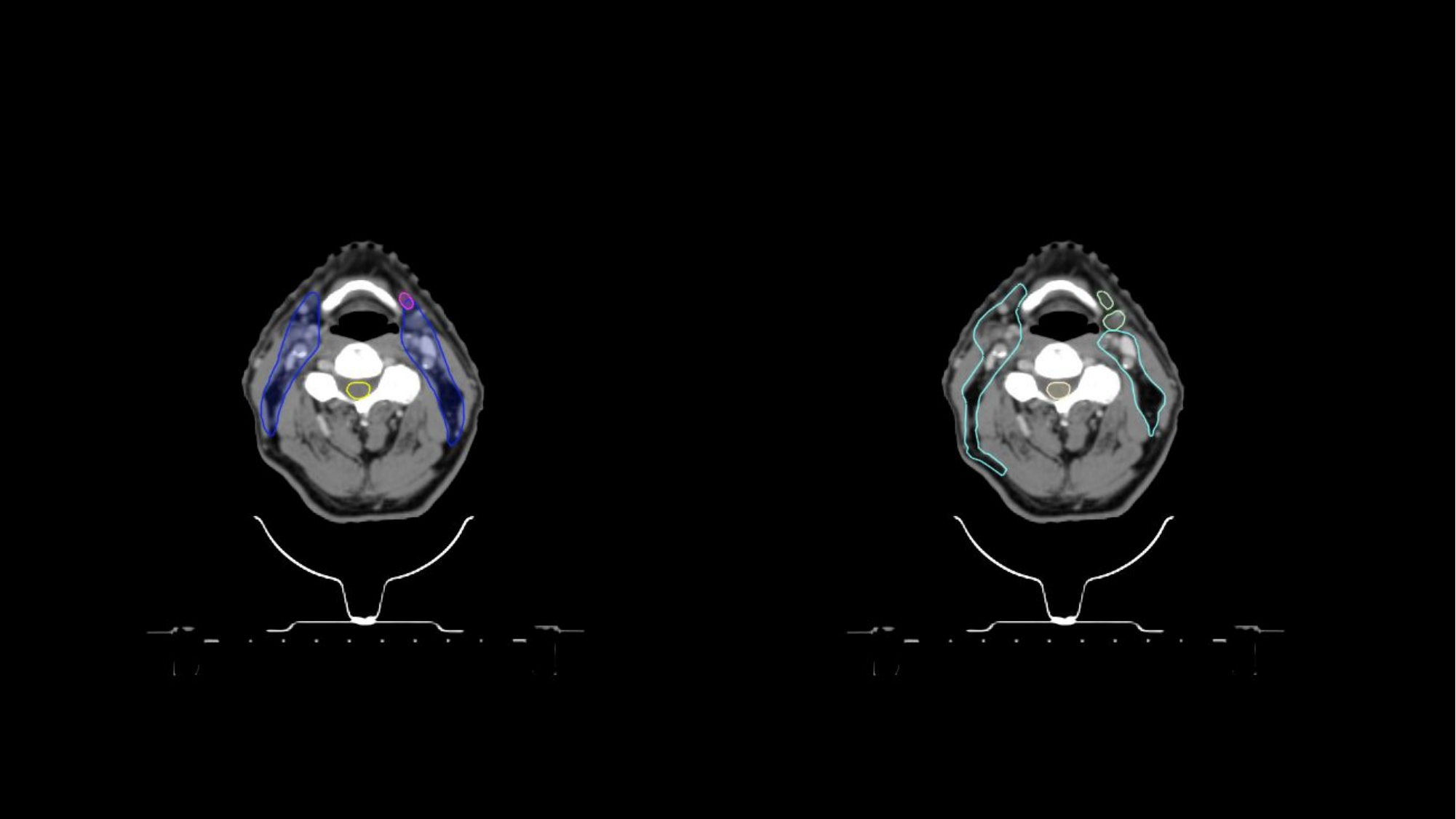

## Slide 30
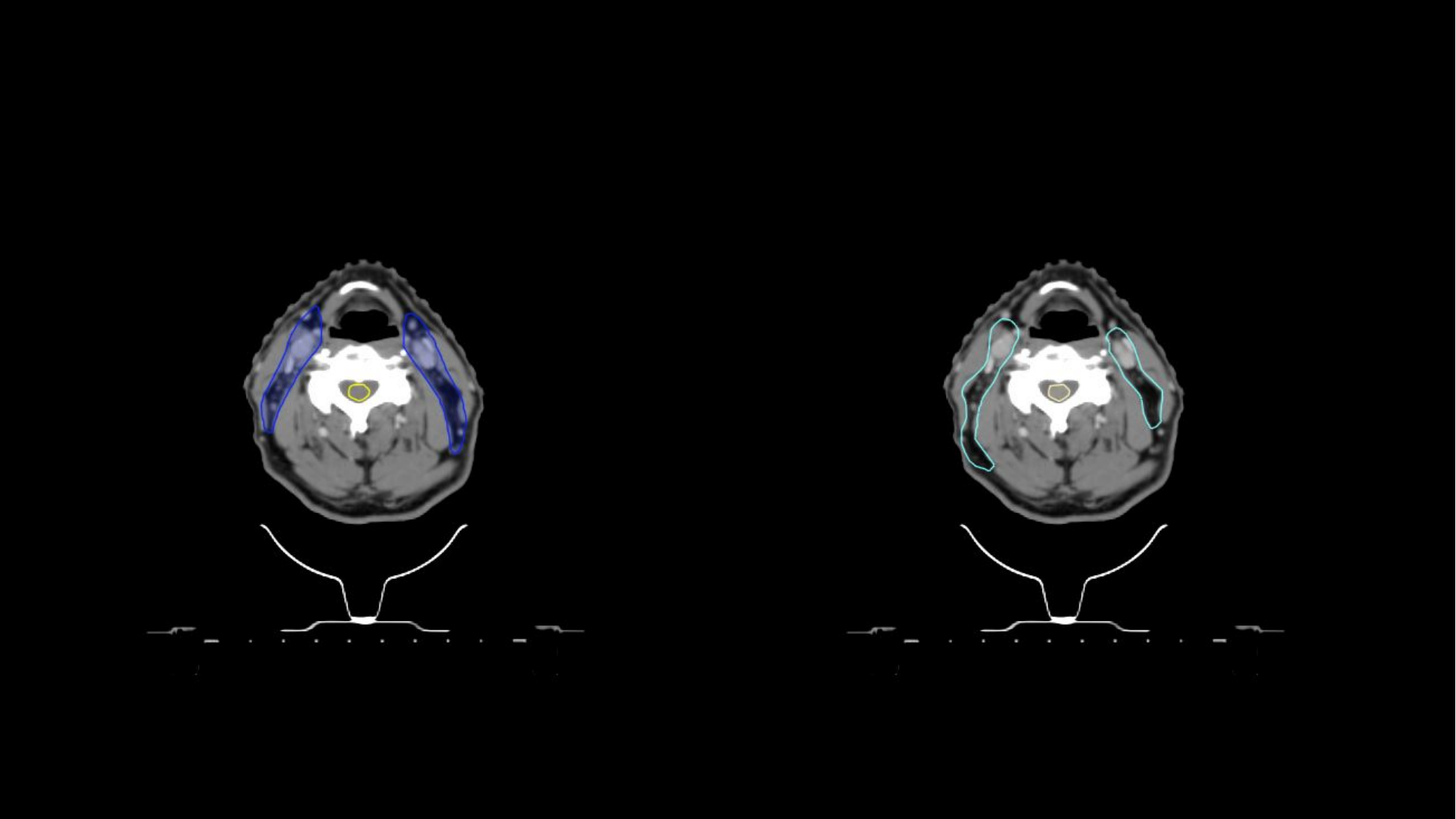

## Slide 31
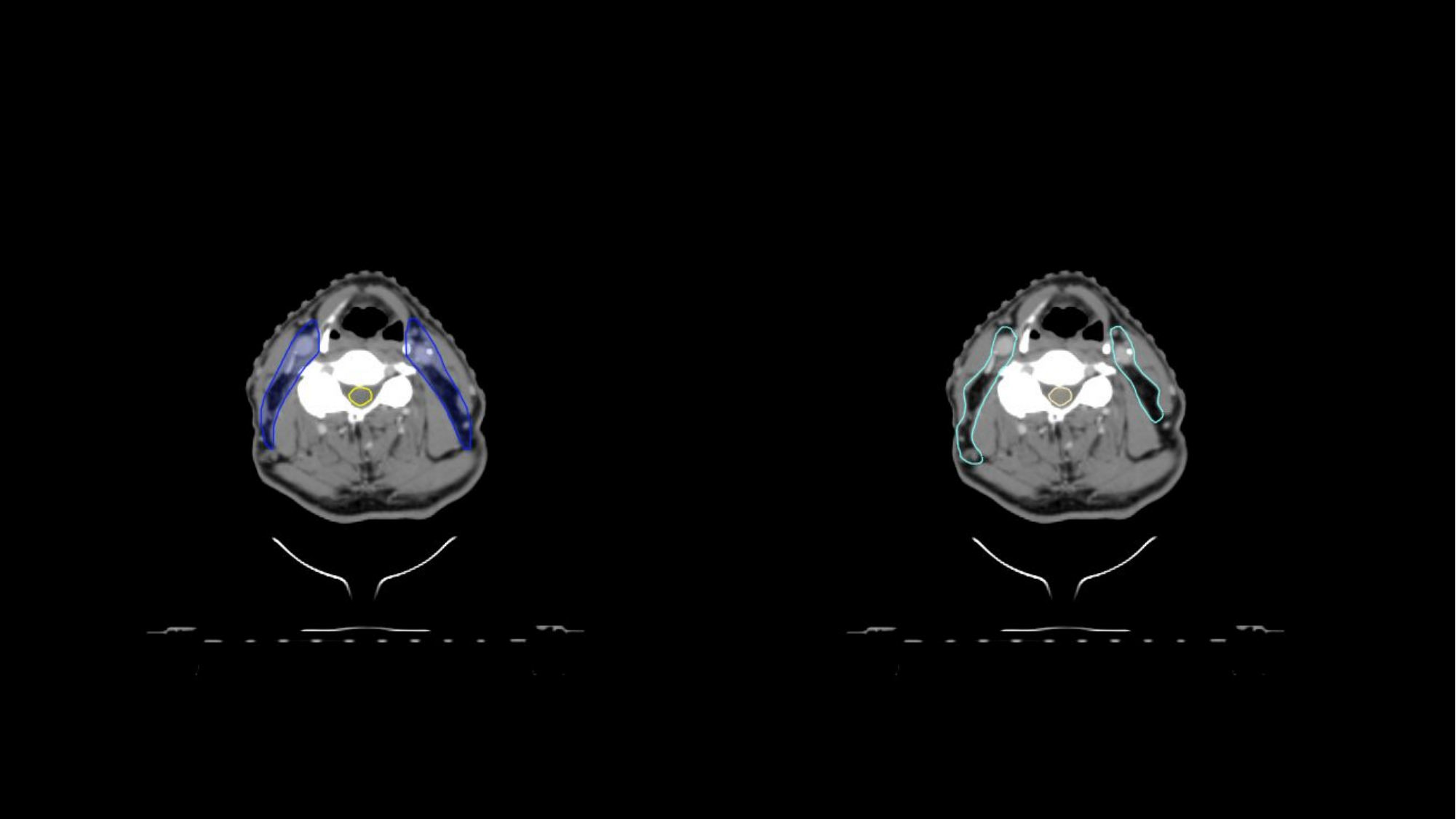

## Slide 32
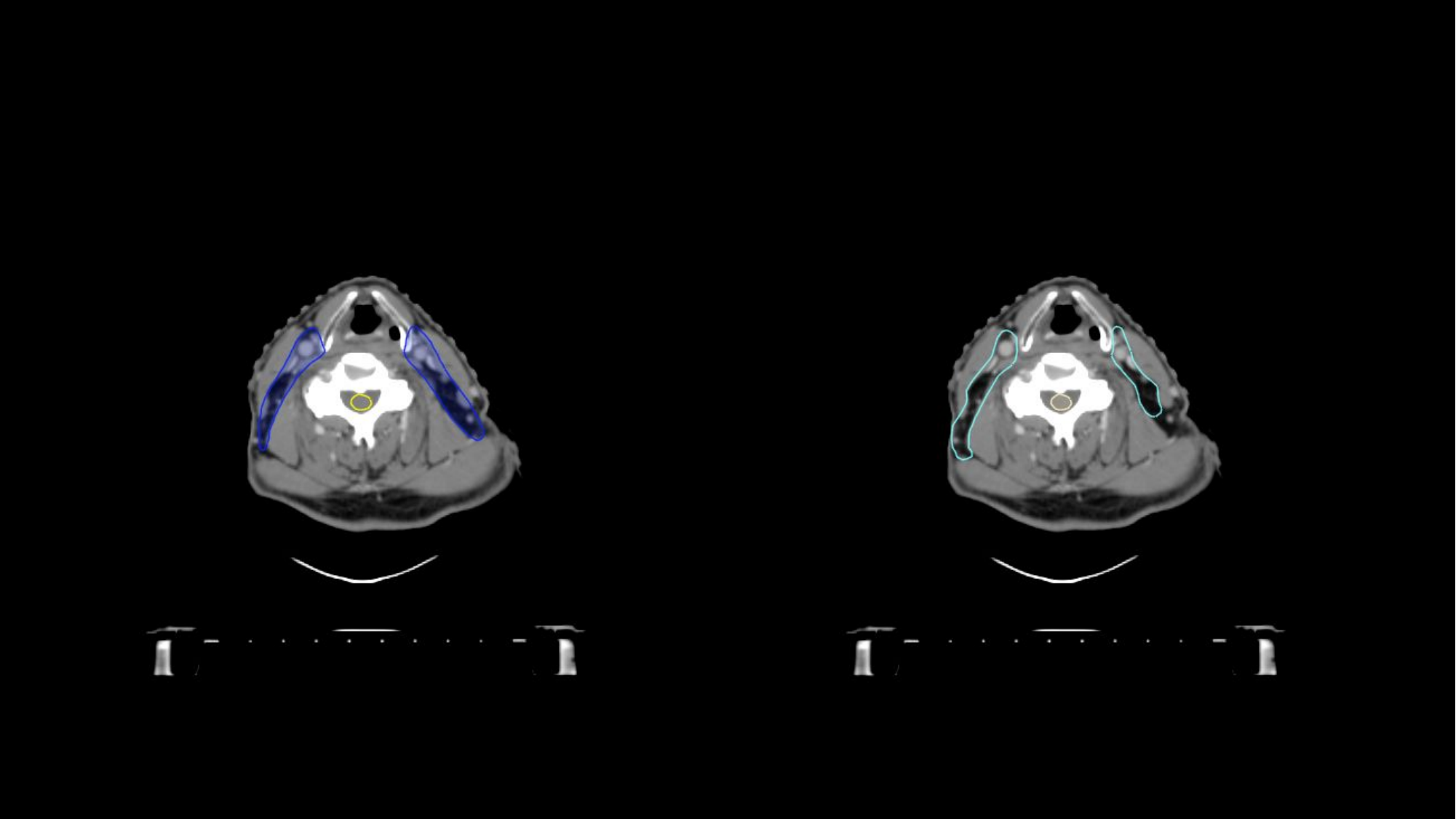

## Slide 33
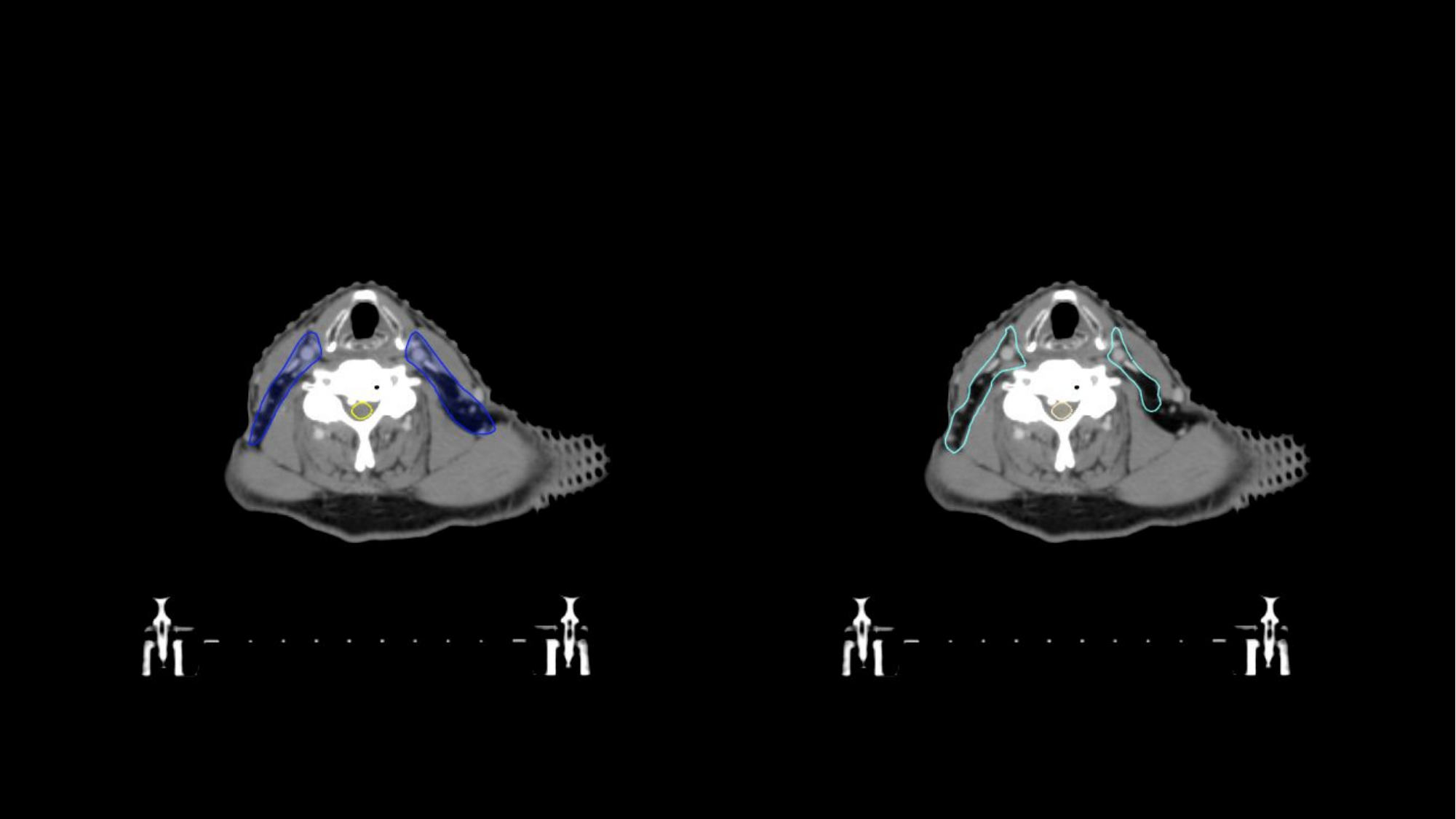

## Slide 34
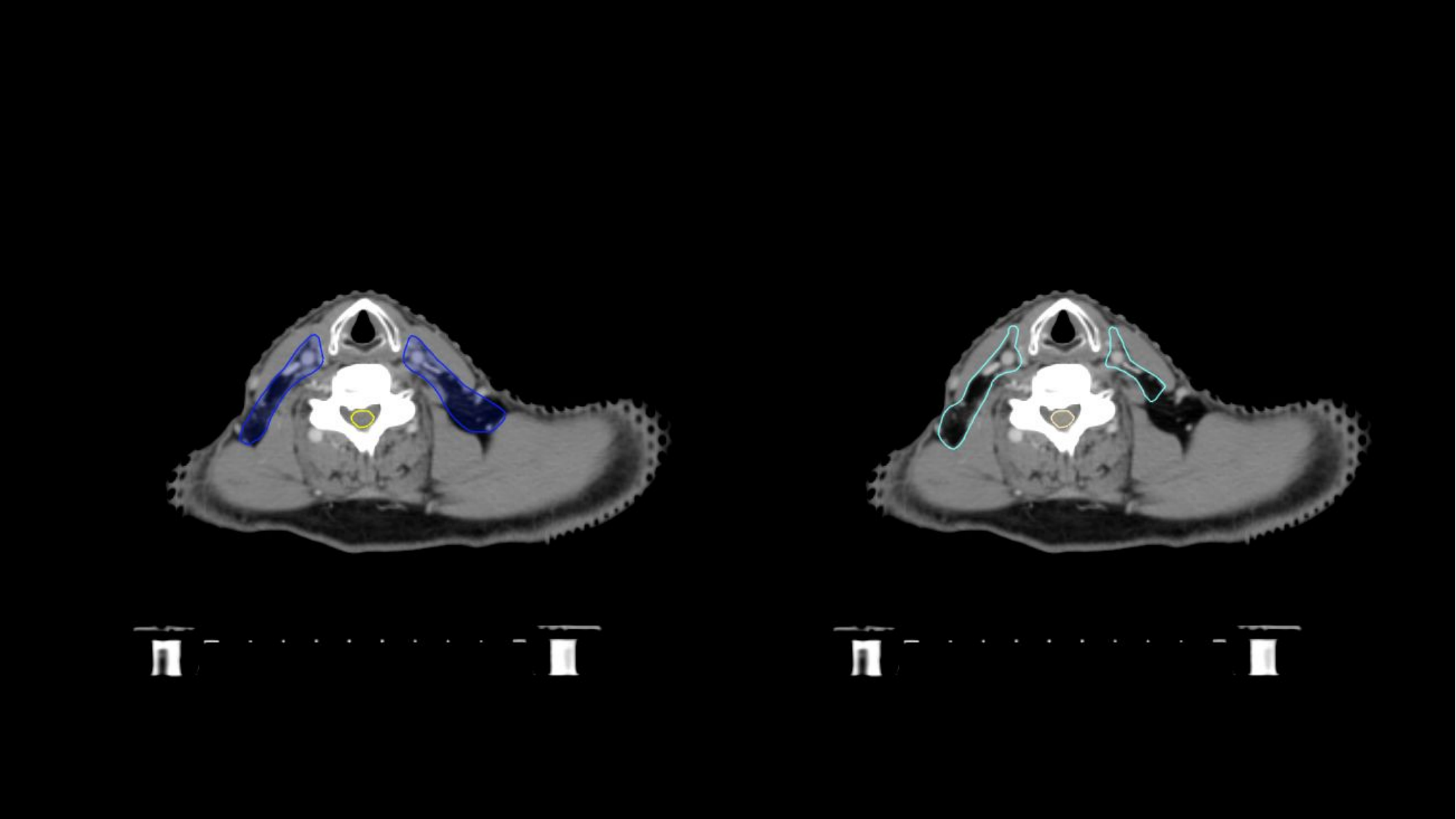

## Slide 35
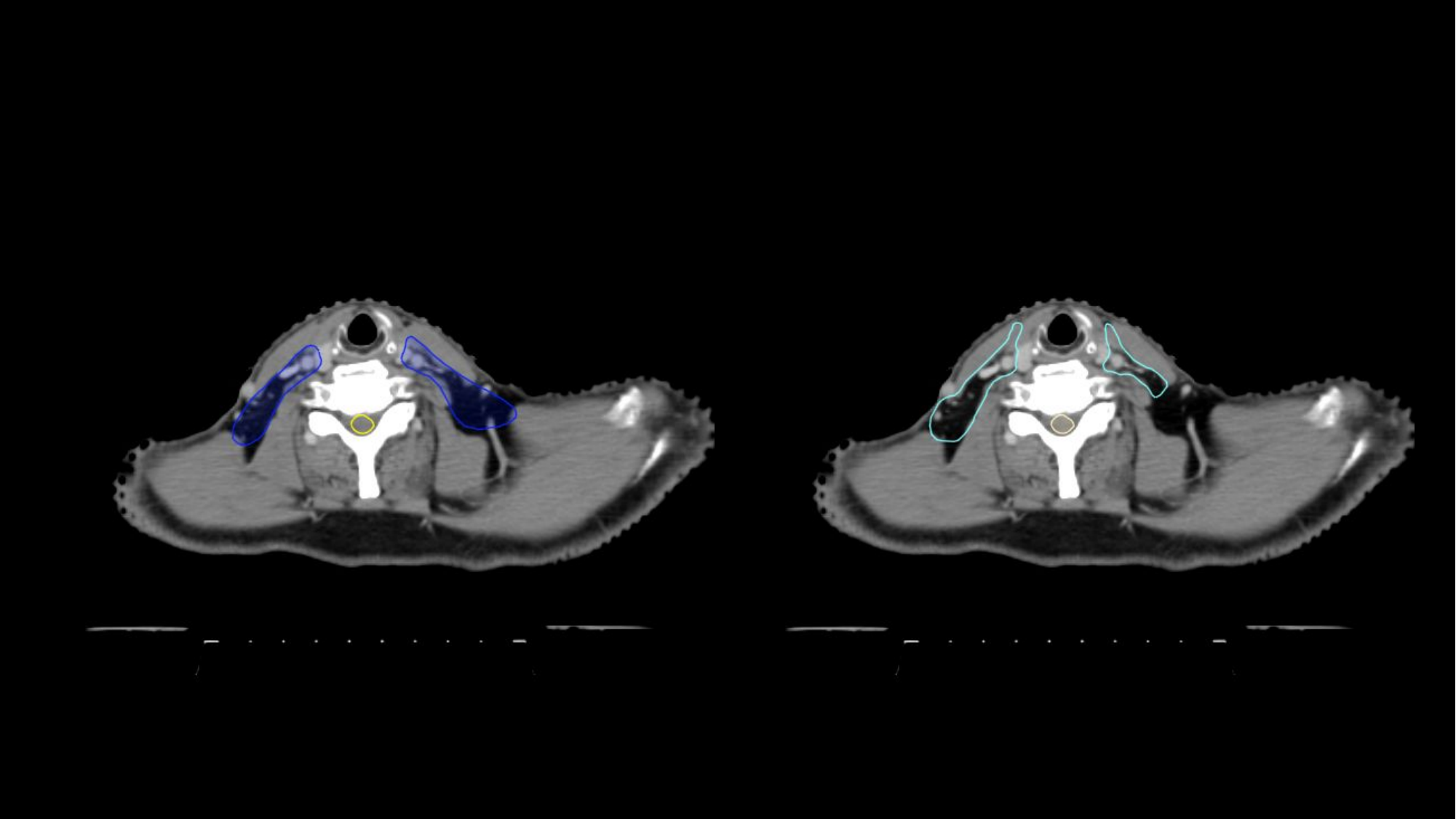

## Slide 36
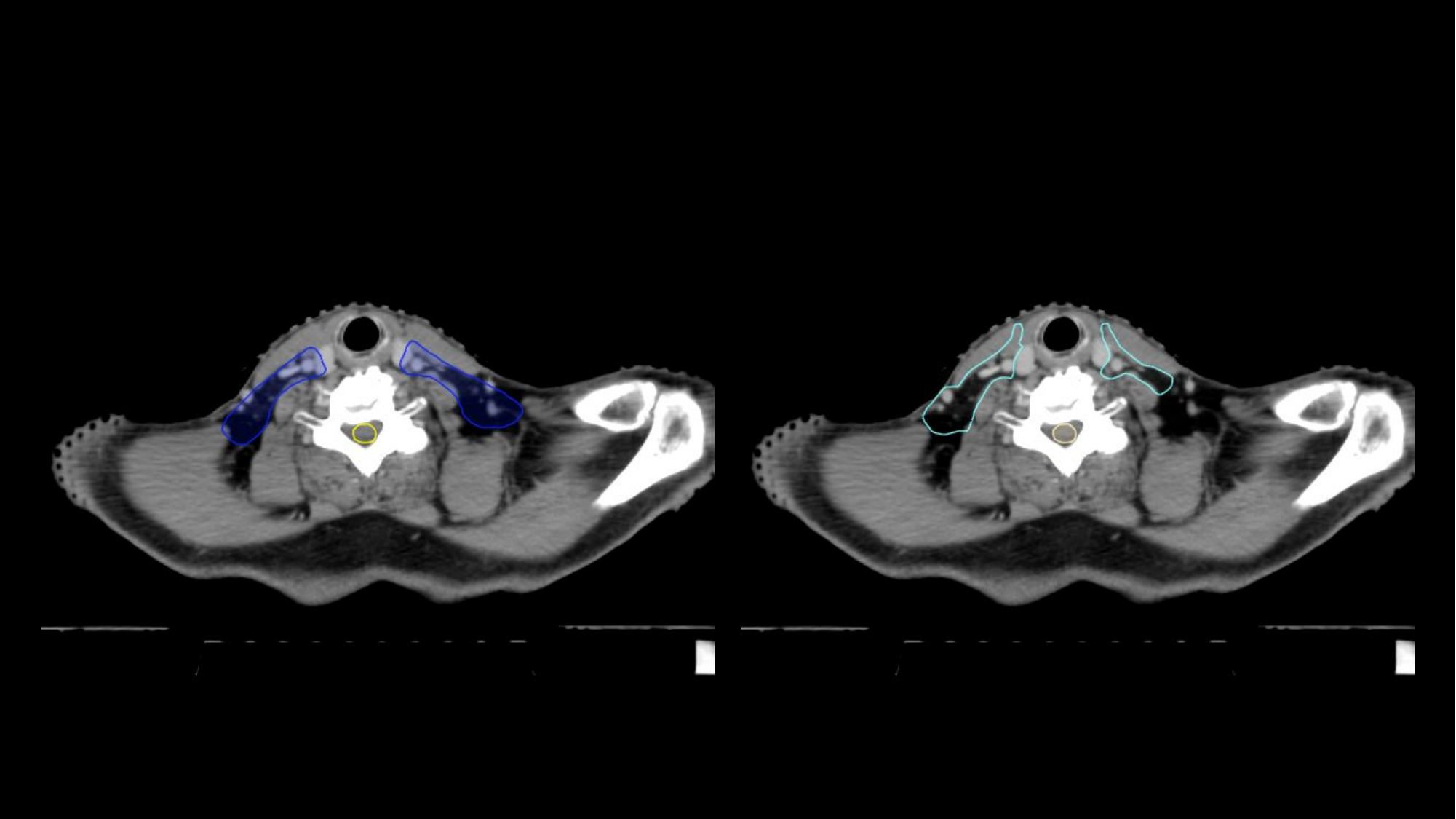

## Slide 37
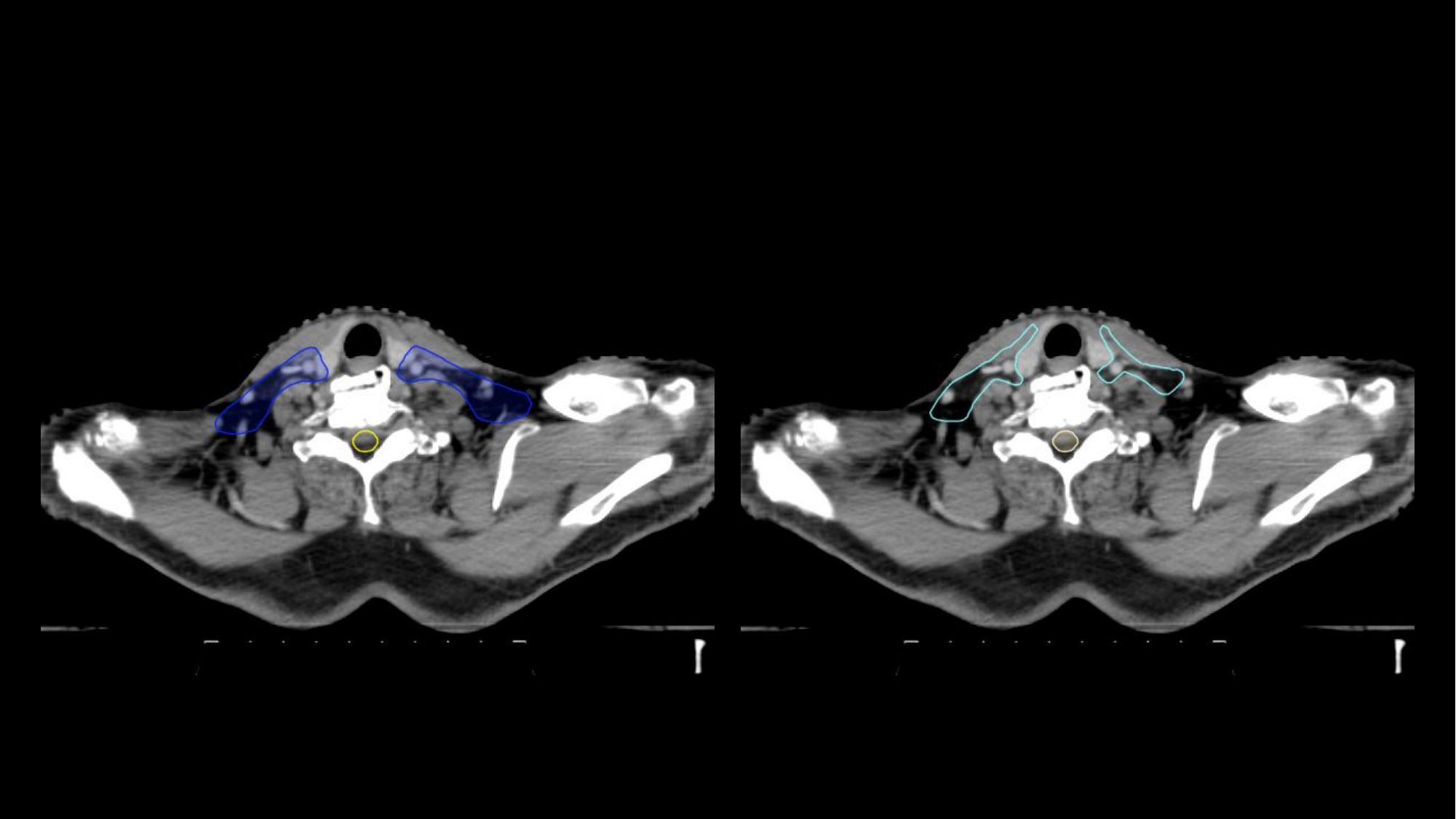

## Slide 38
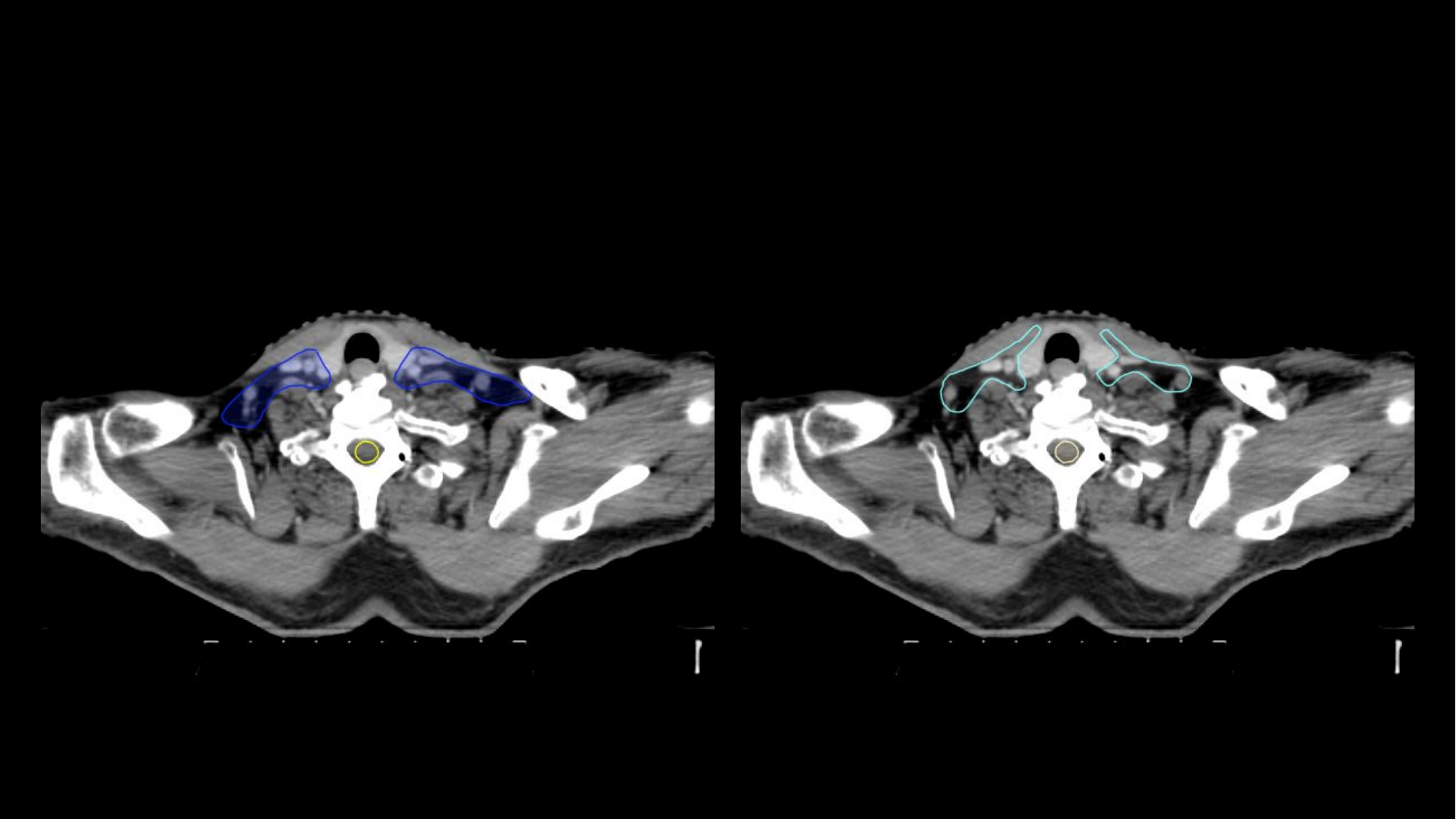

## Slide 39
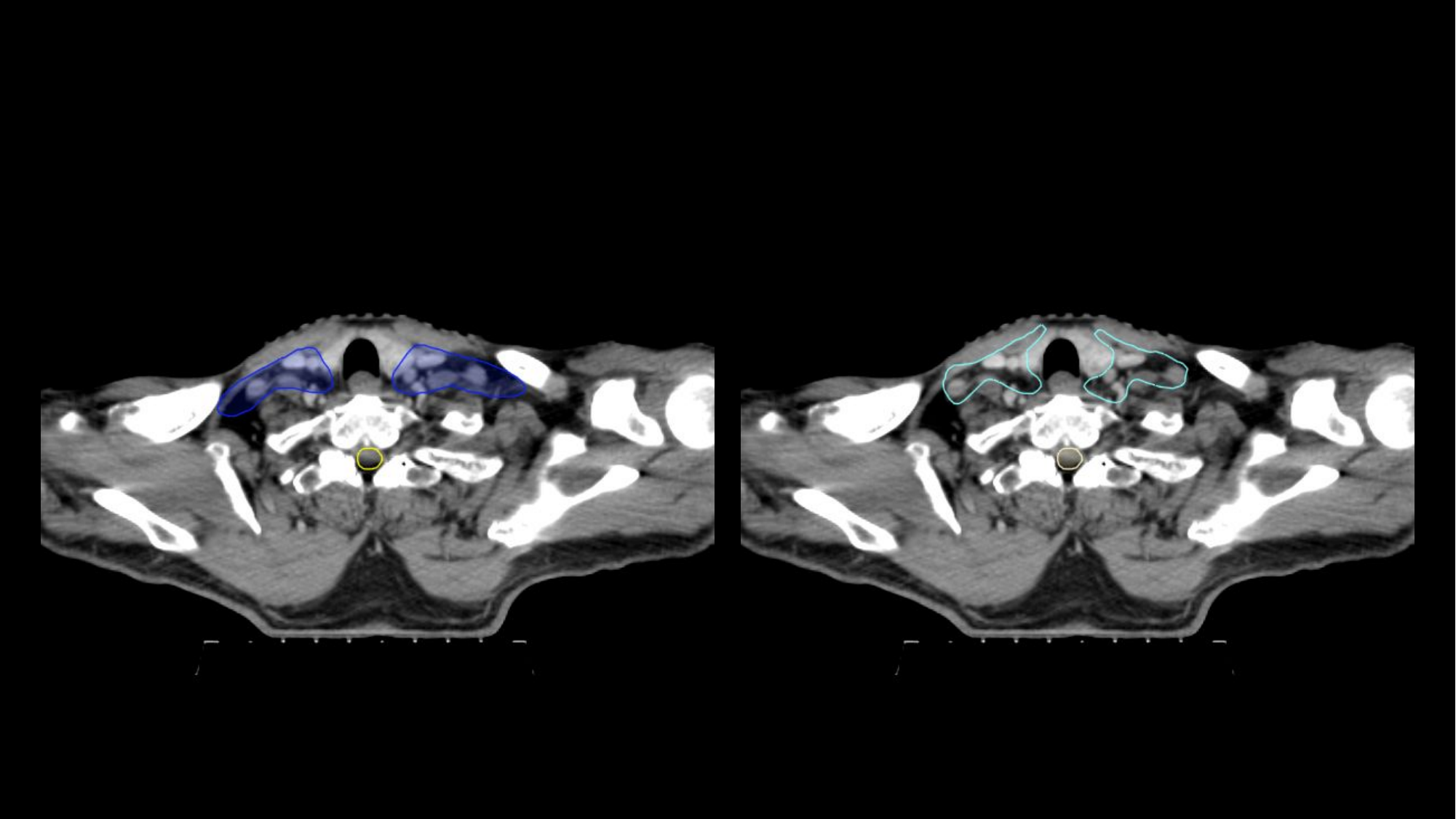

## Slide 40
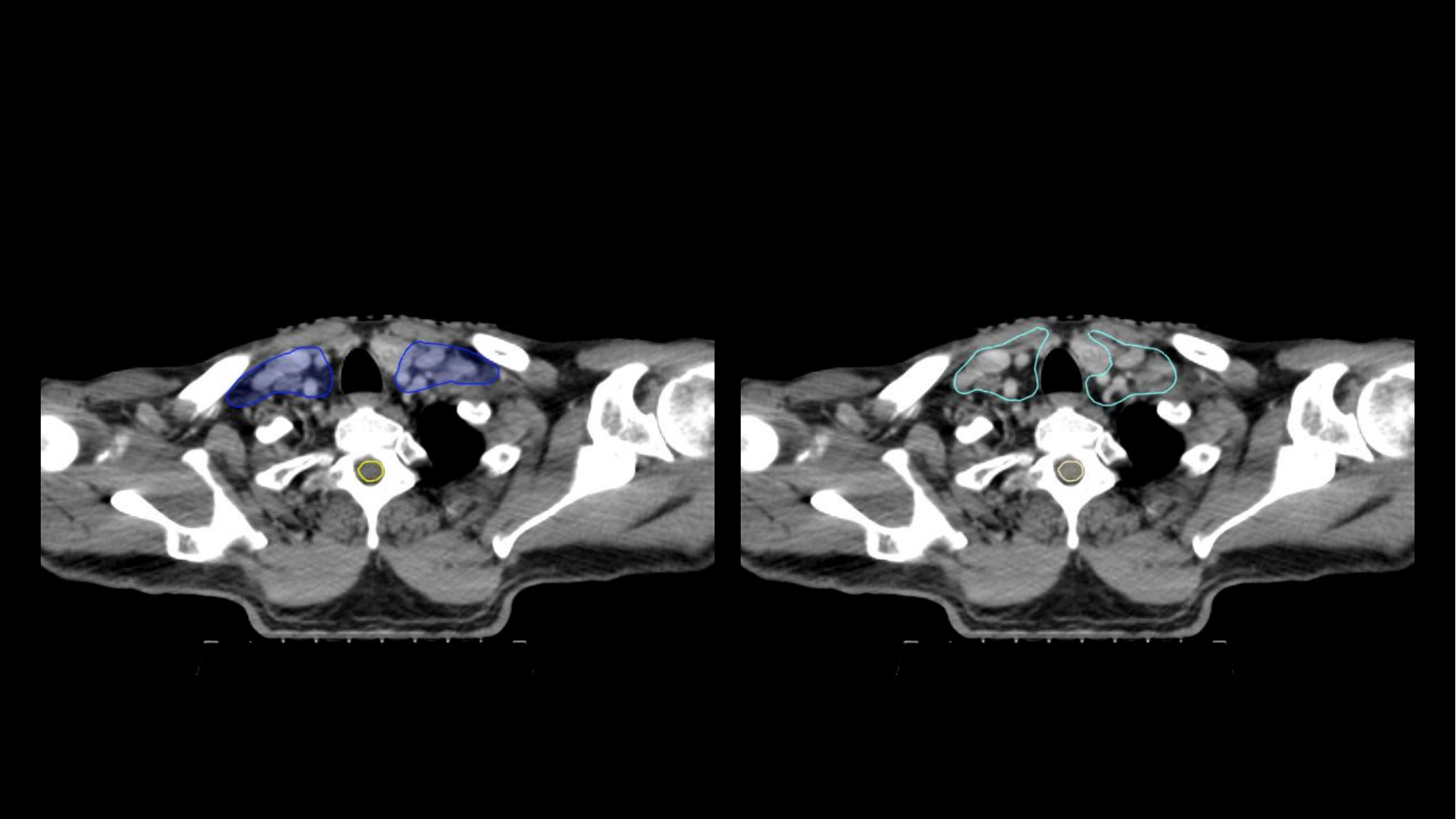

## Slide 41
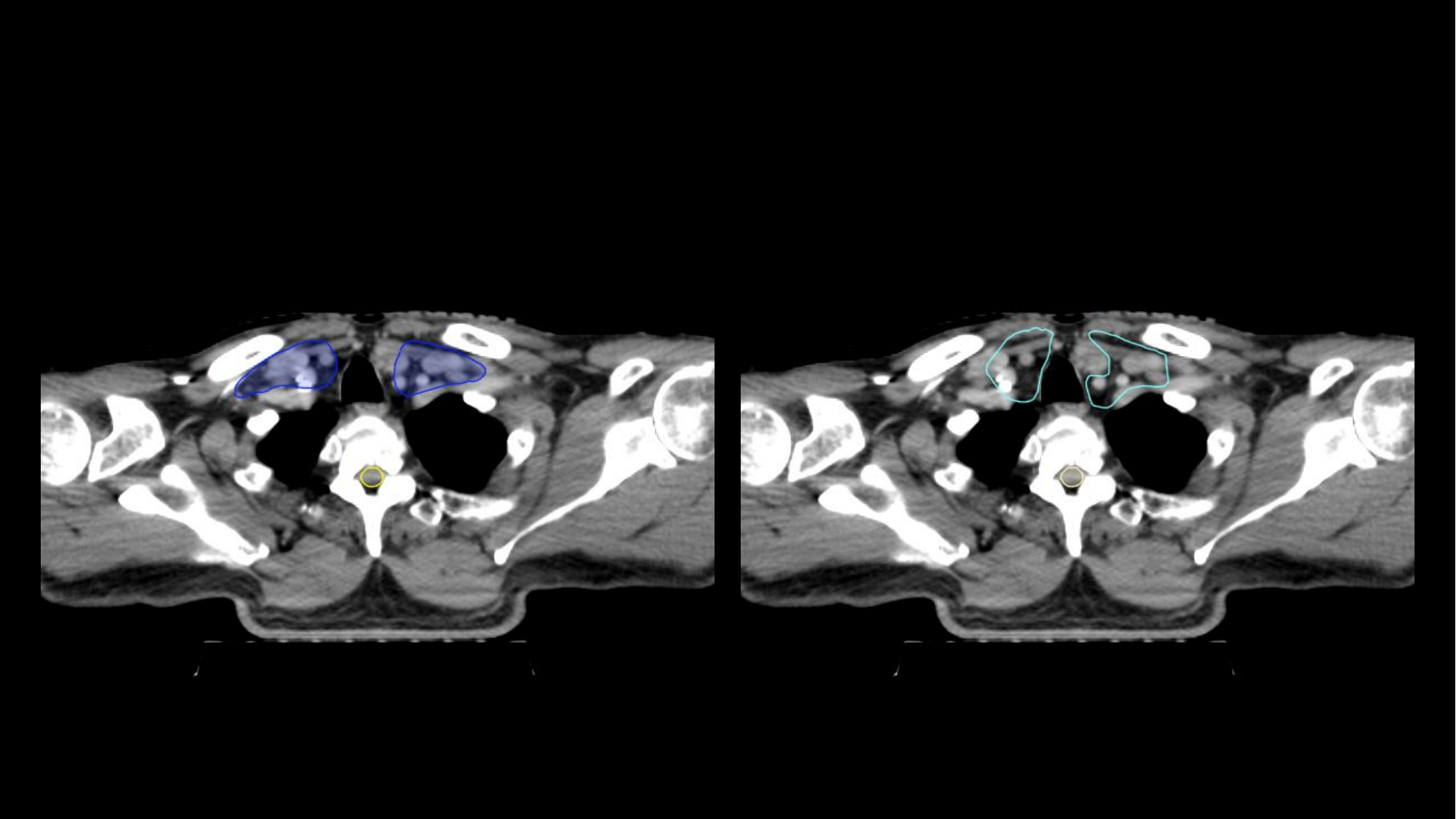

## Slide 42
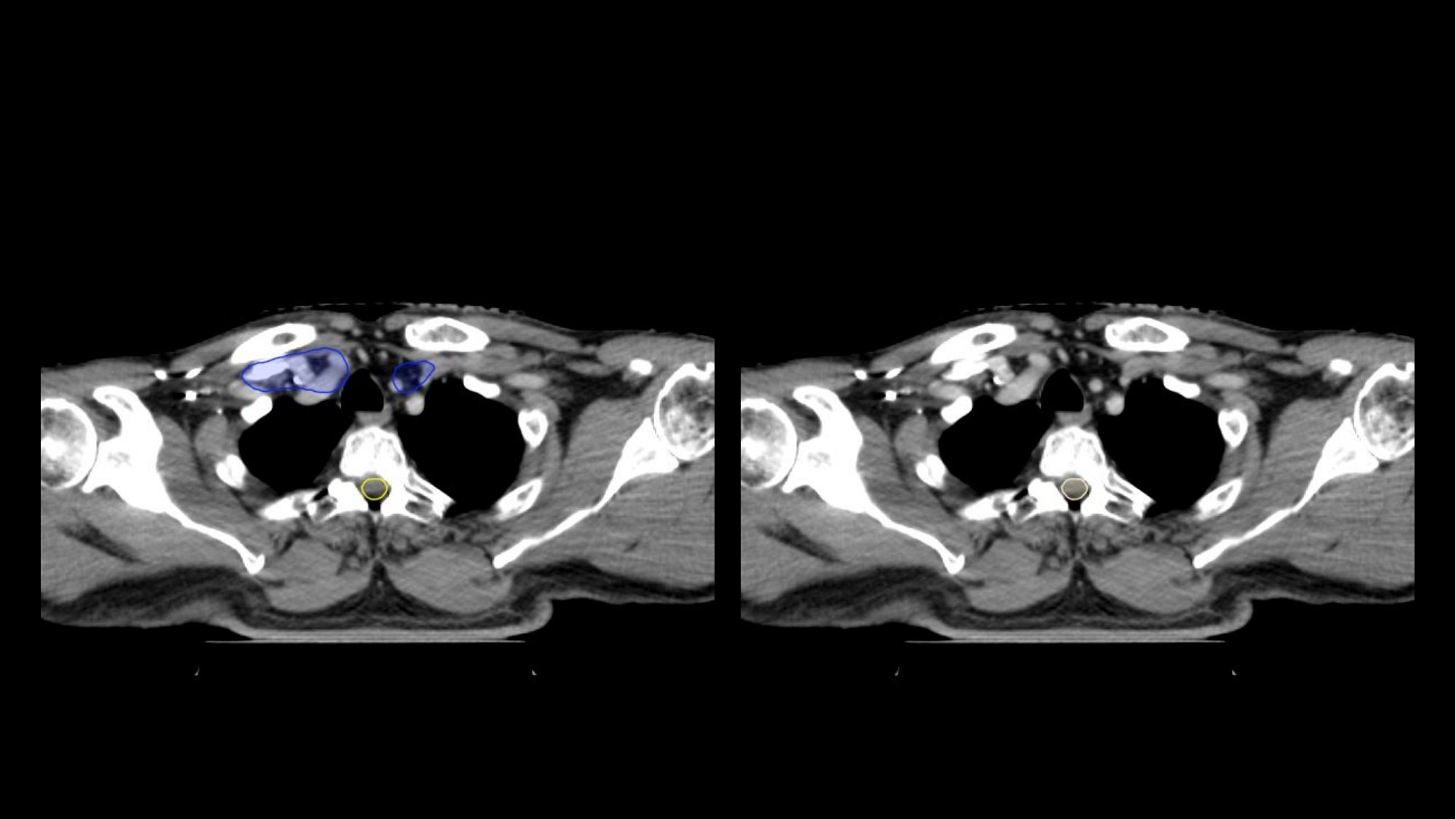

## Slide 43
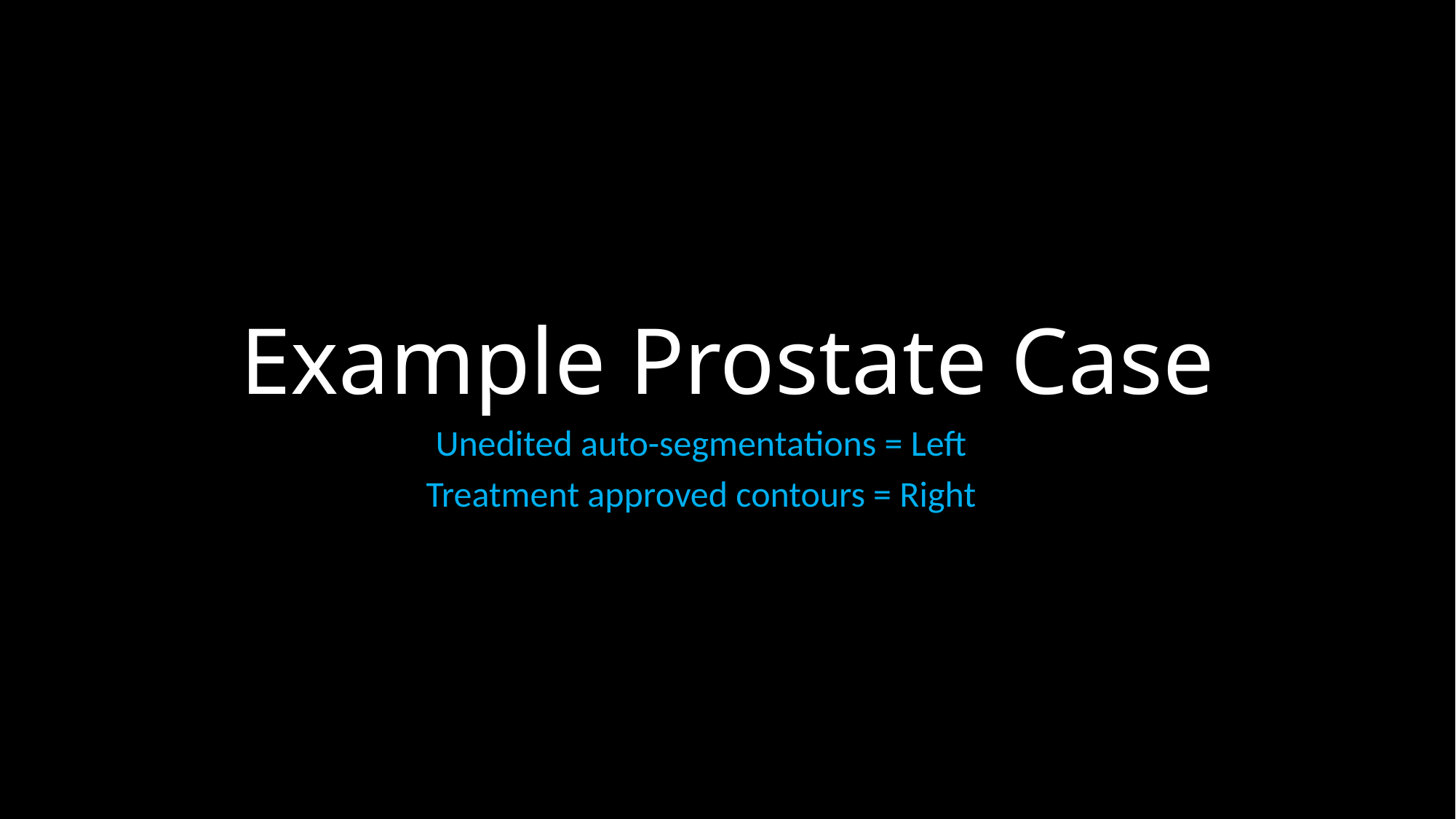

# Example Prostate Case
Unedited auto-segmentations = Left
Treatment approved contours = Right

## Slide 44
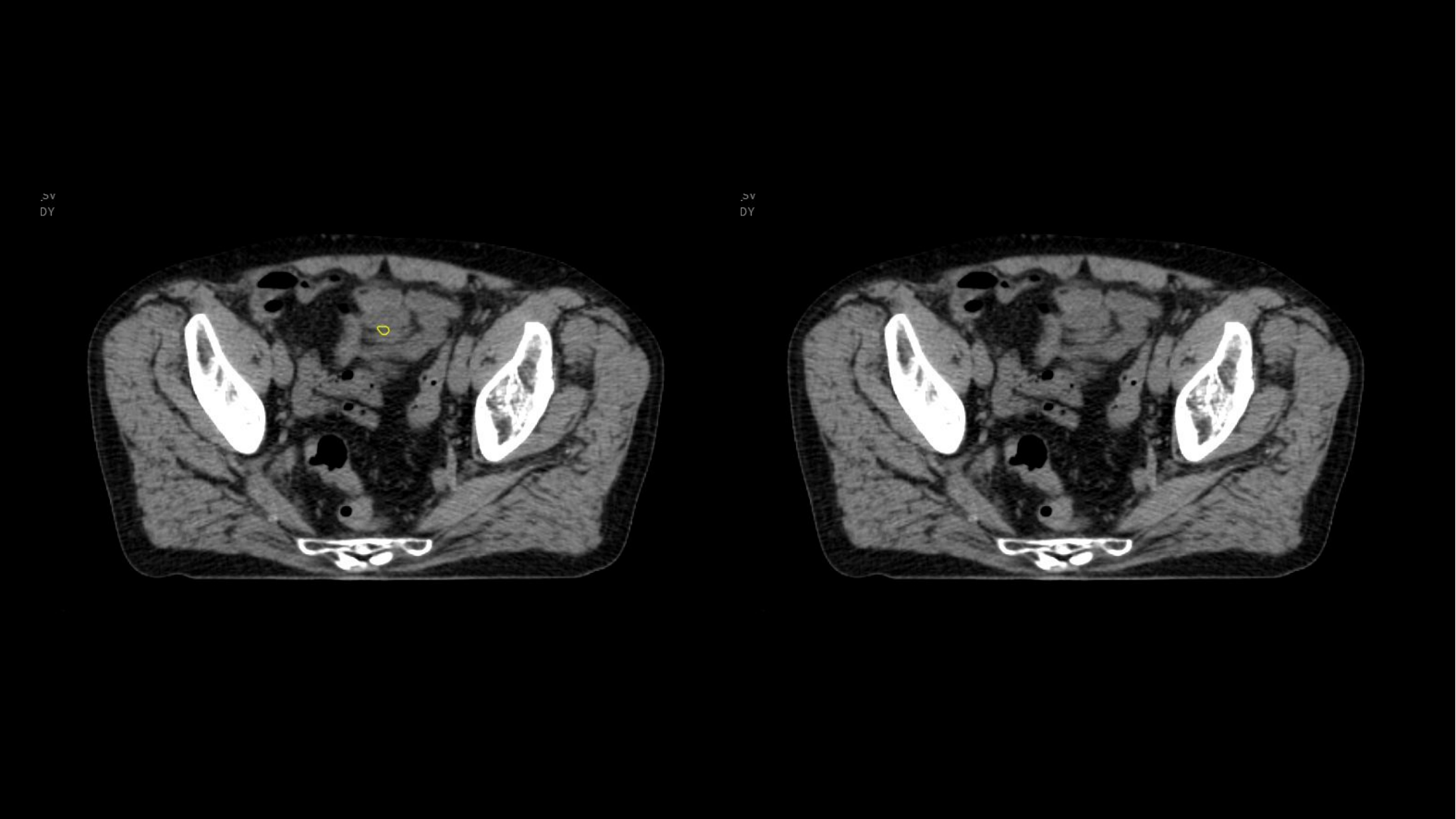

## Slide 45
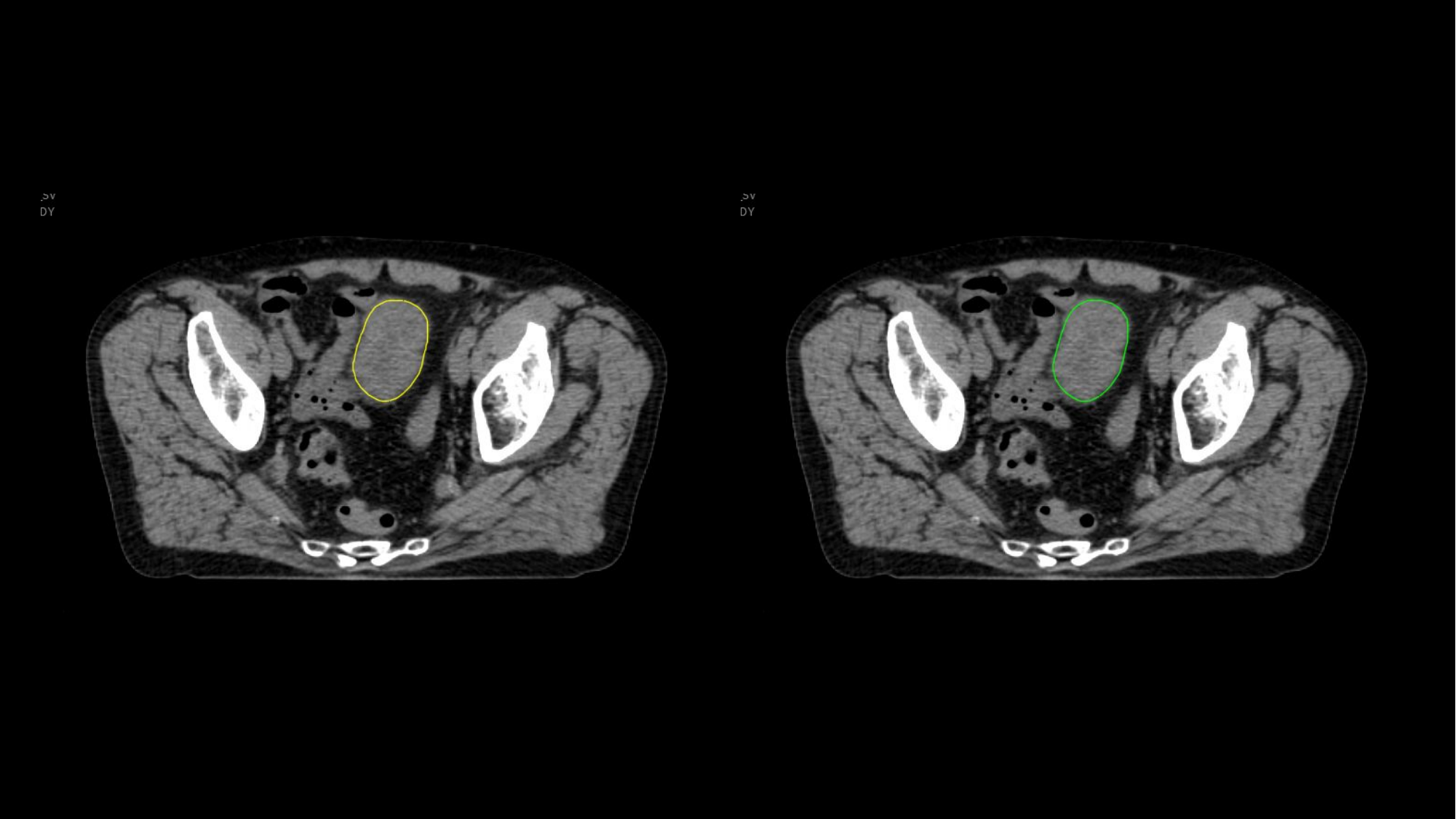

## Slide 46
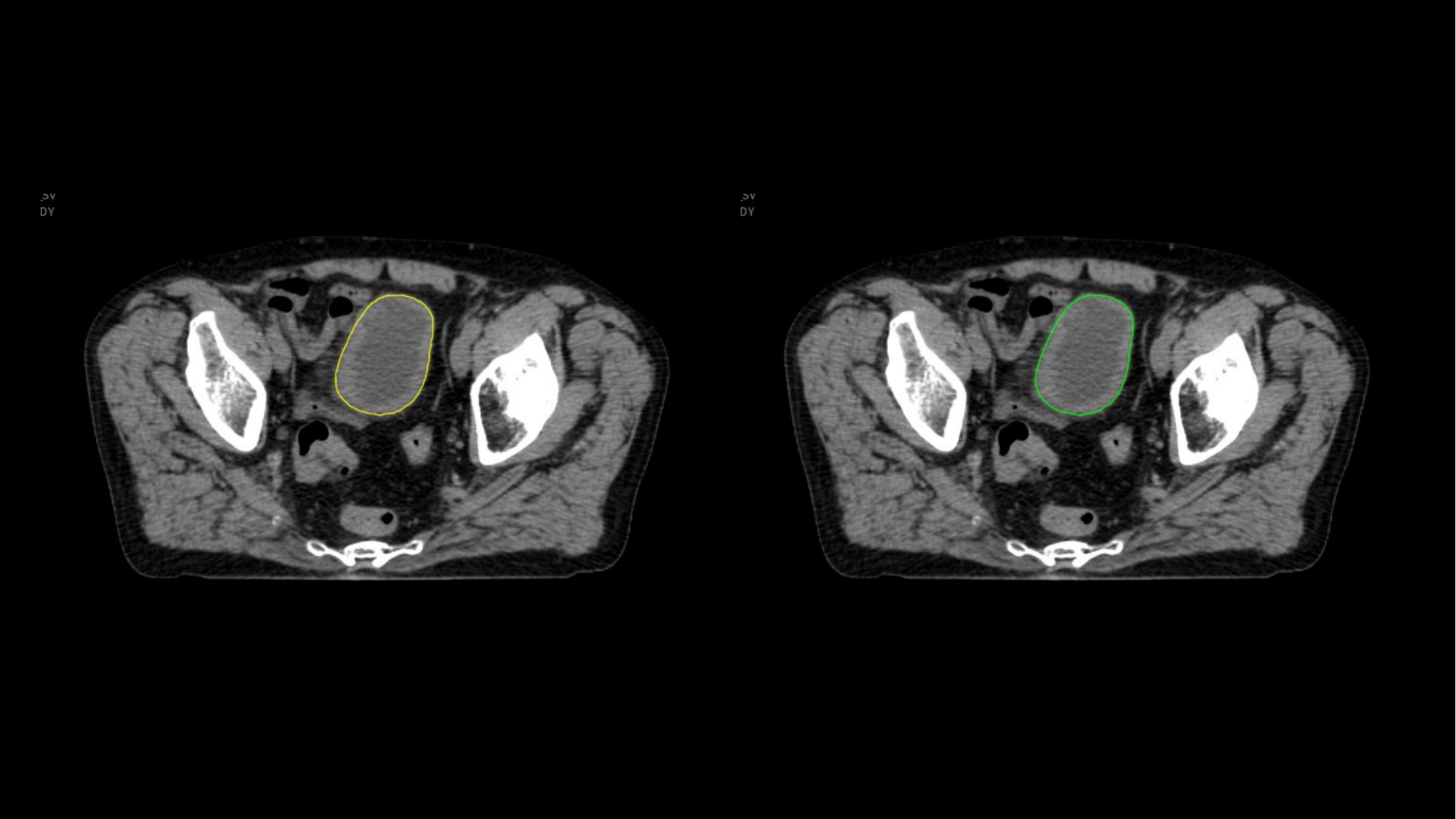

## Slide 47
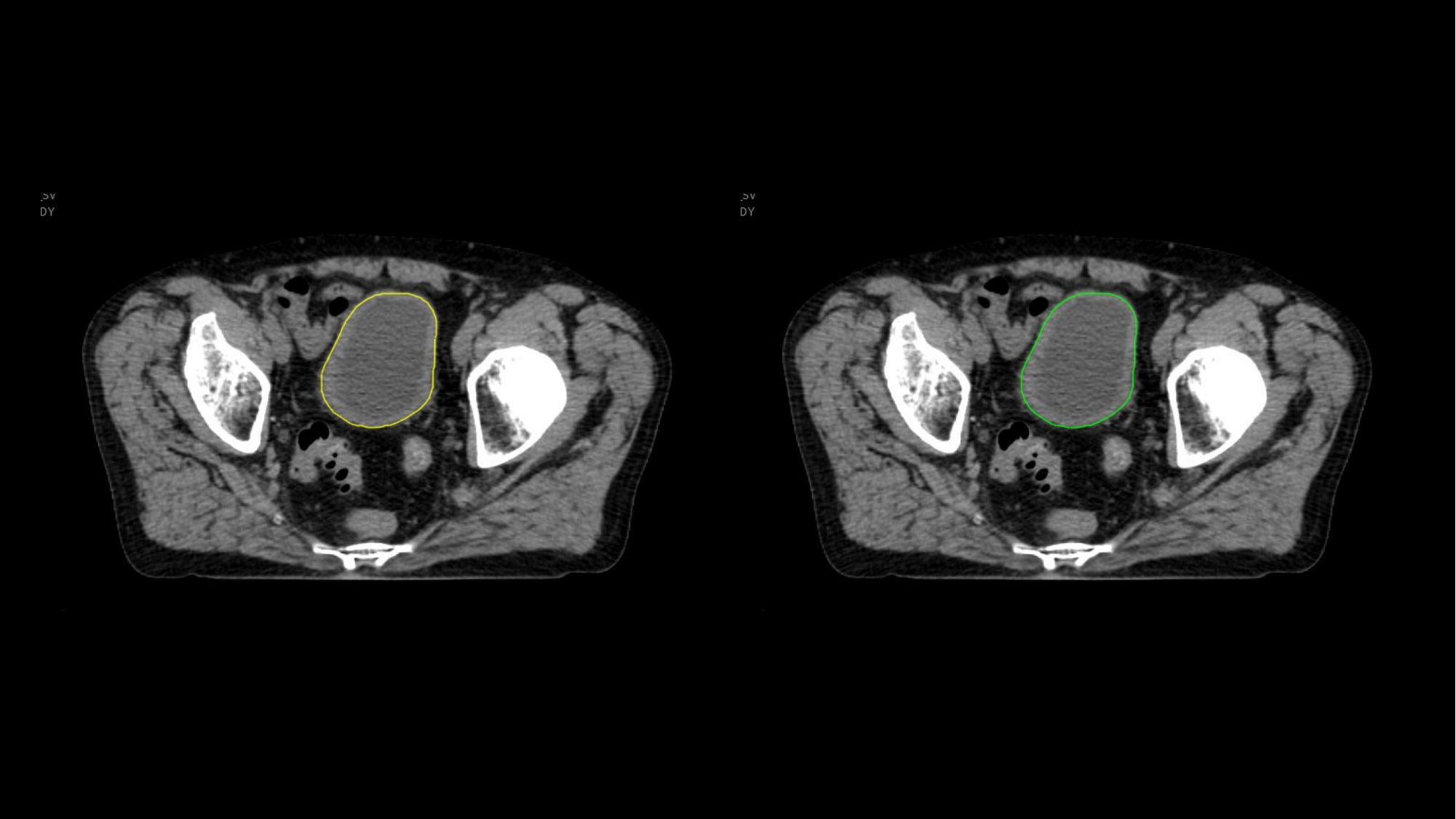

## Slide 48
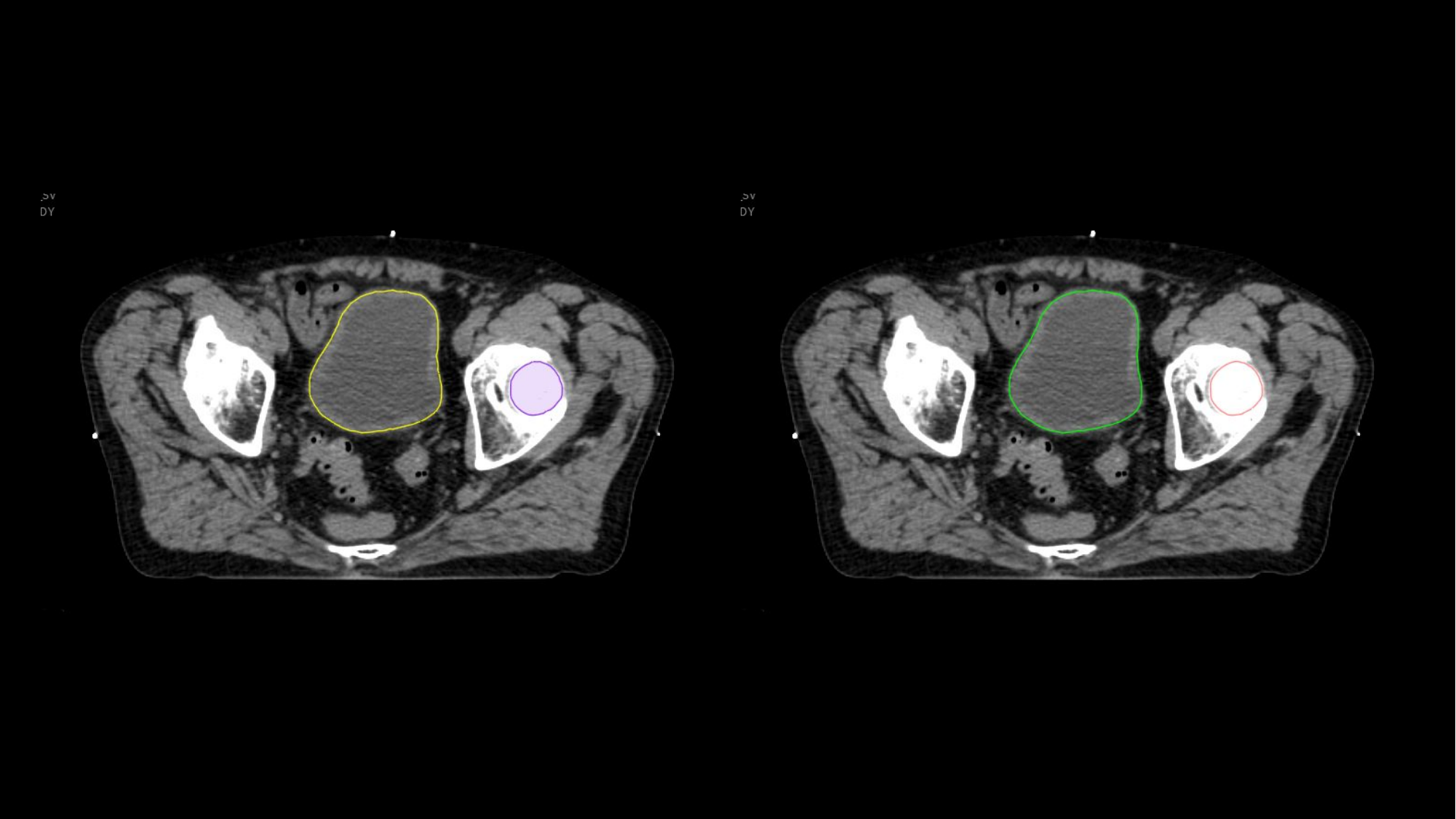

## Slide 49
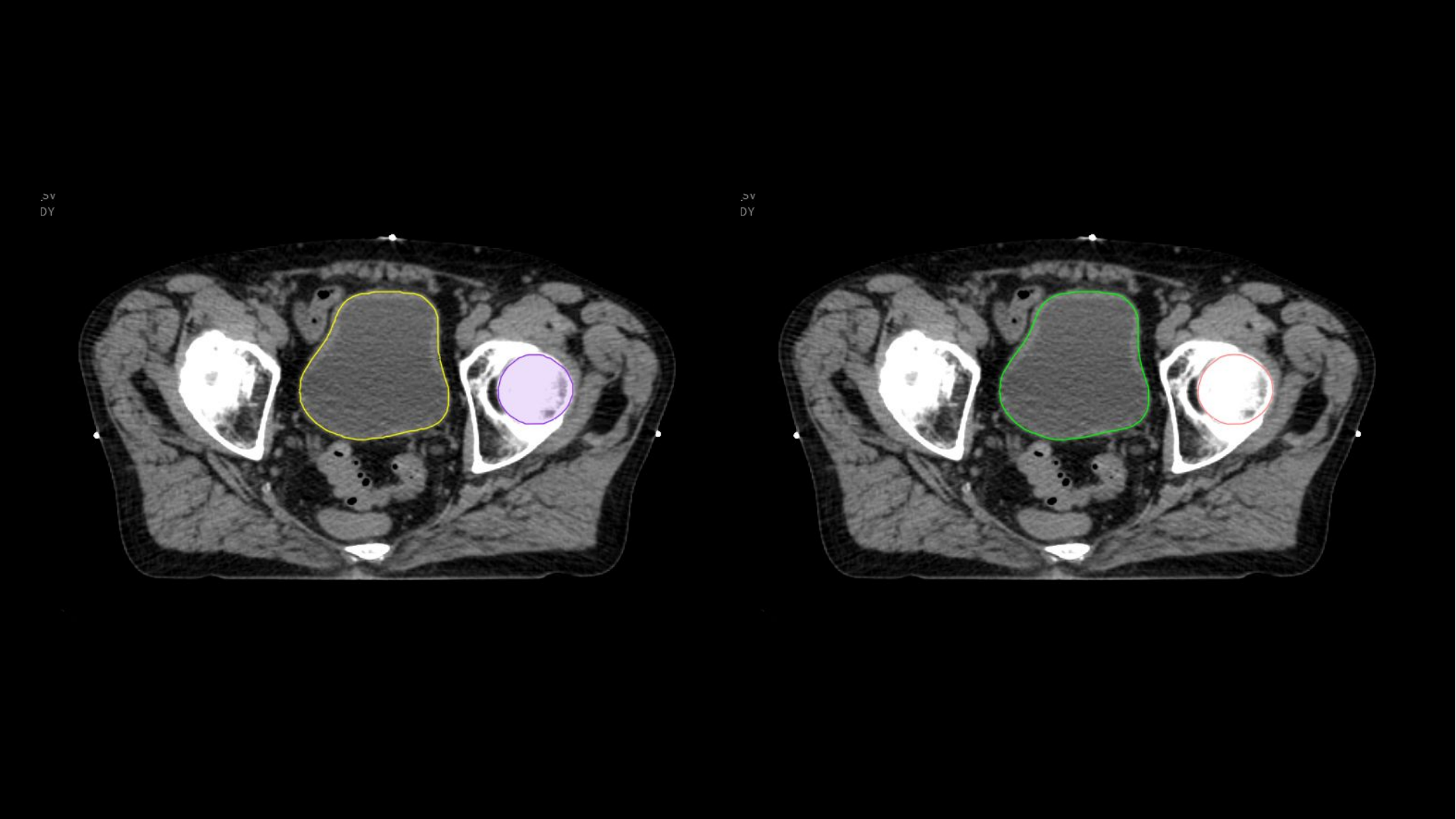

## Slide 50
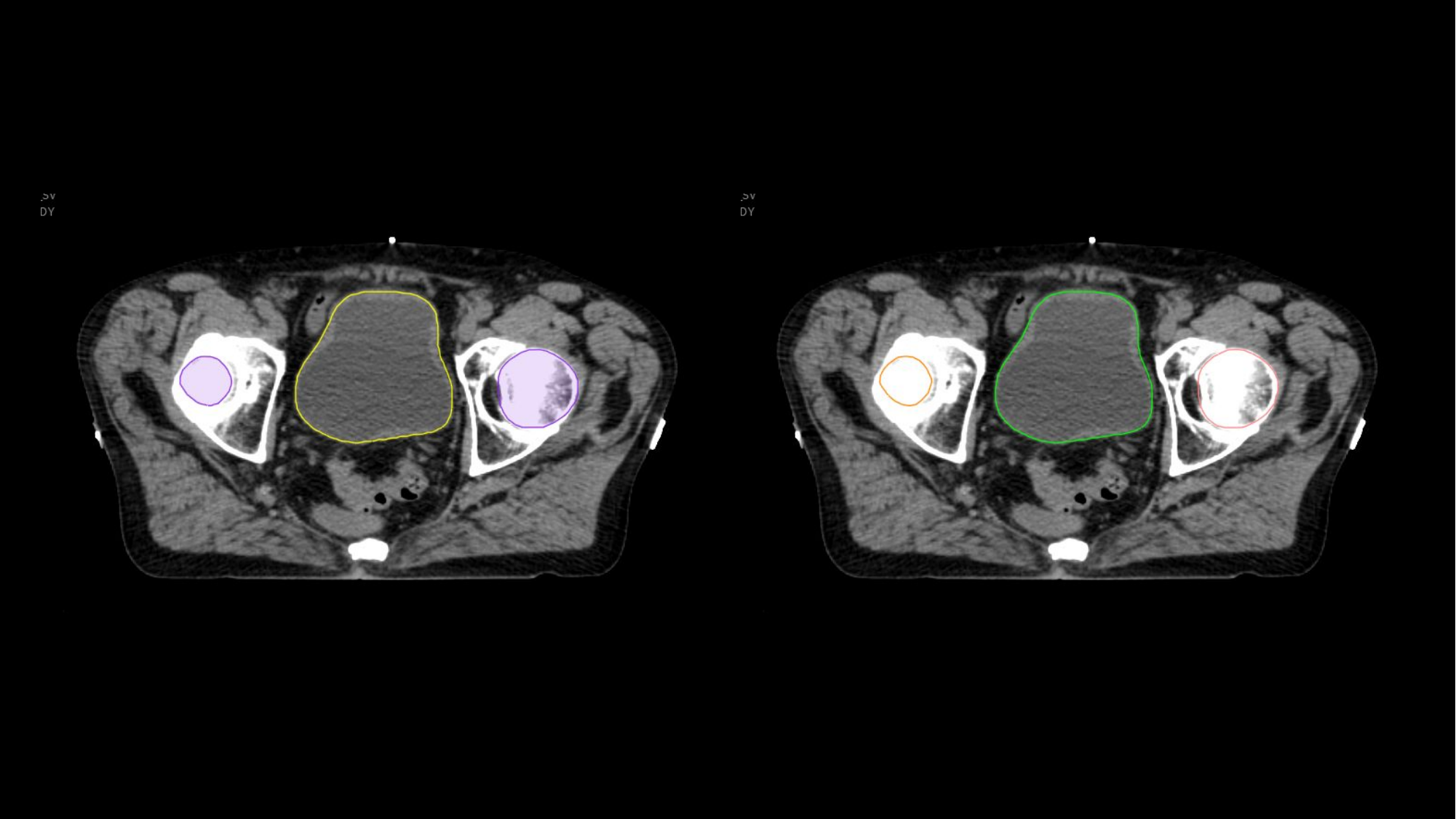

## Slide 51
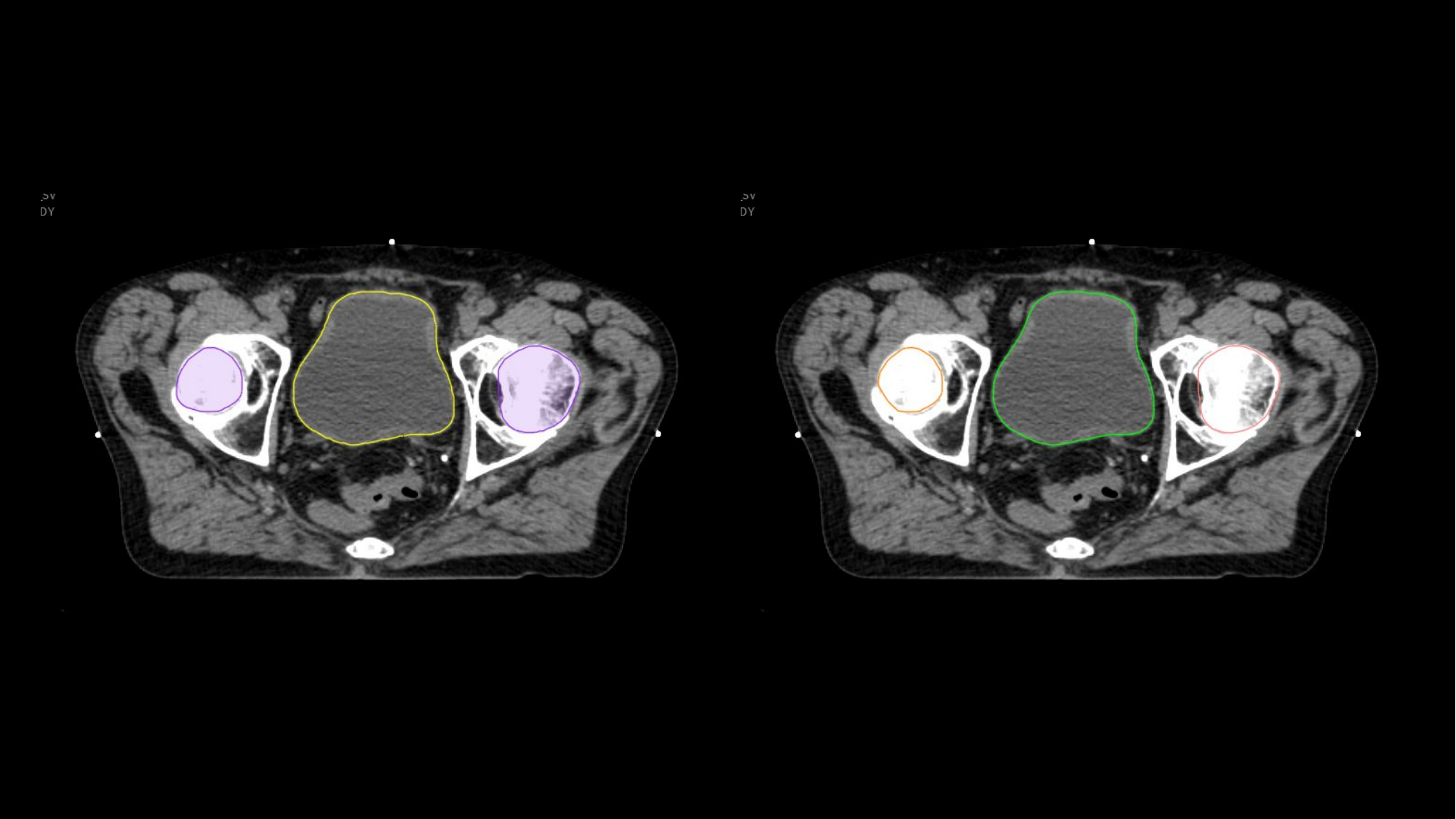

## Slide 52
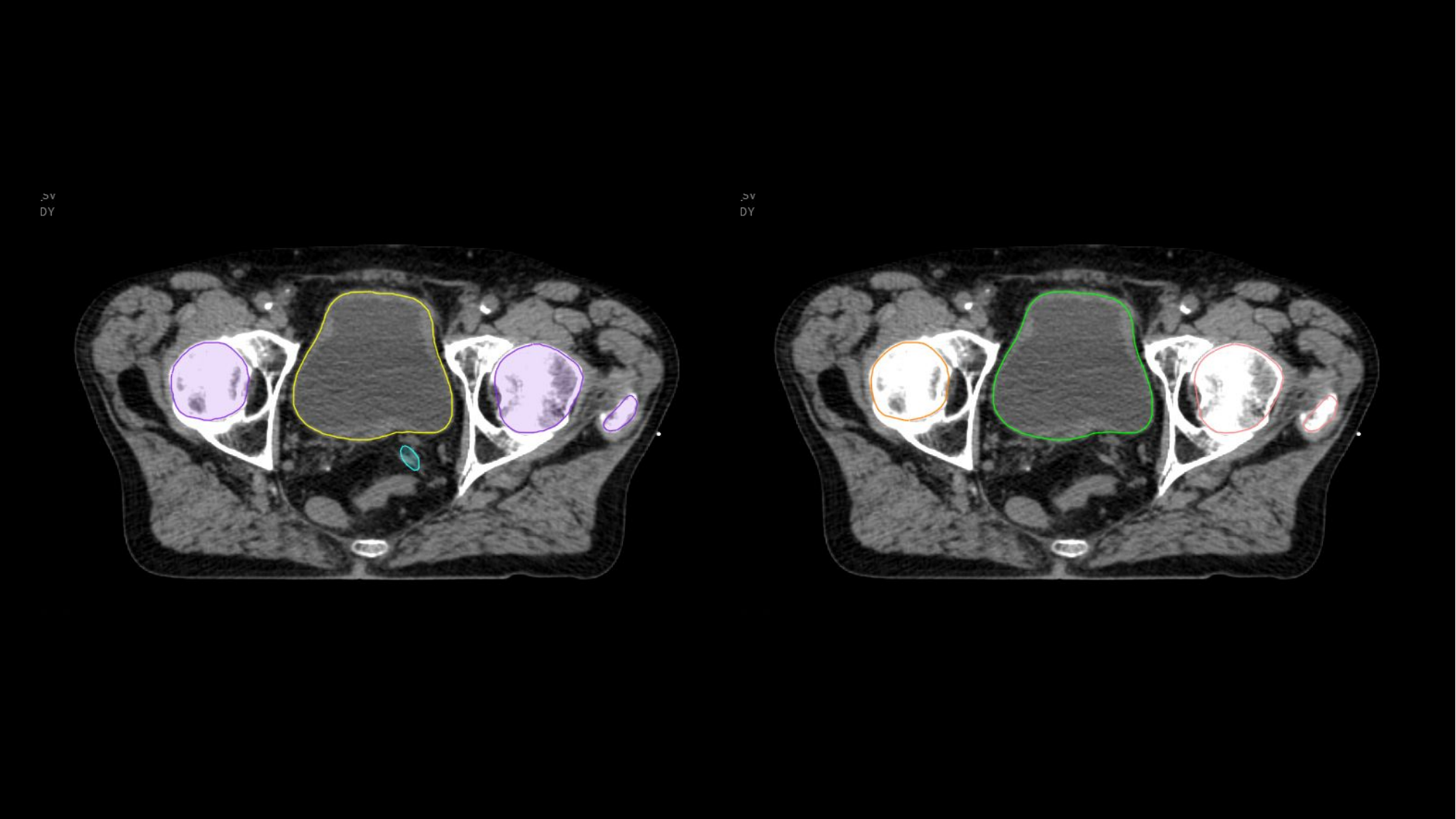

## Slide 53
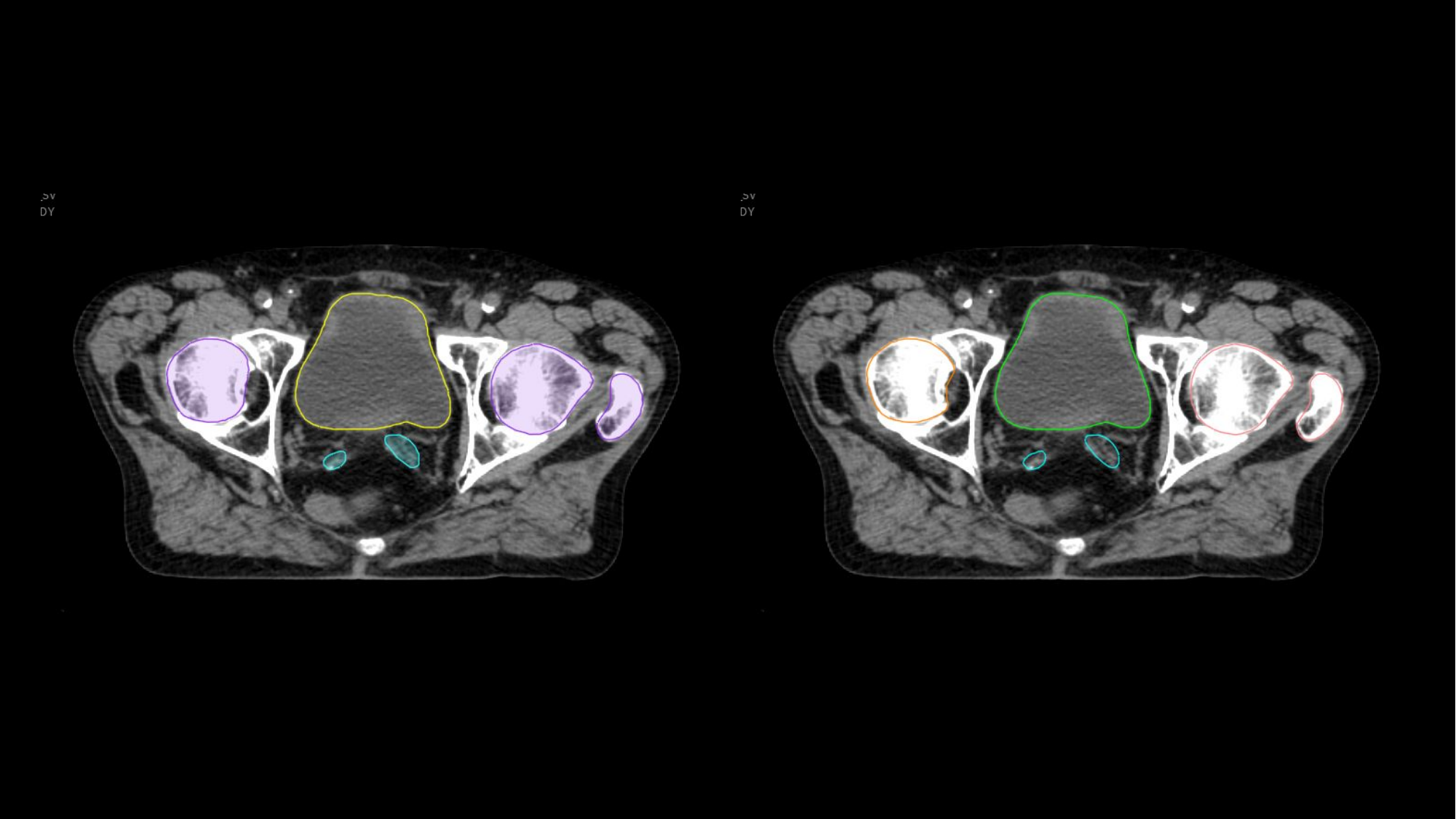

## Slide 54
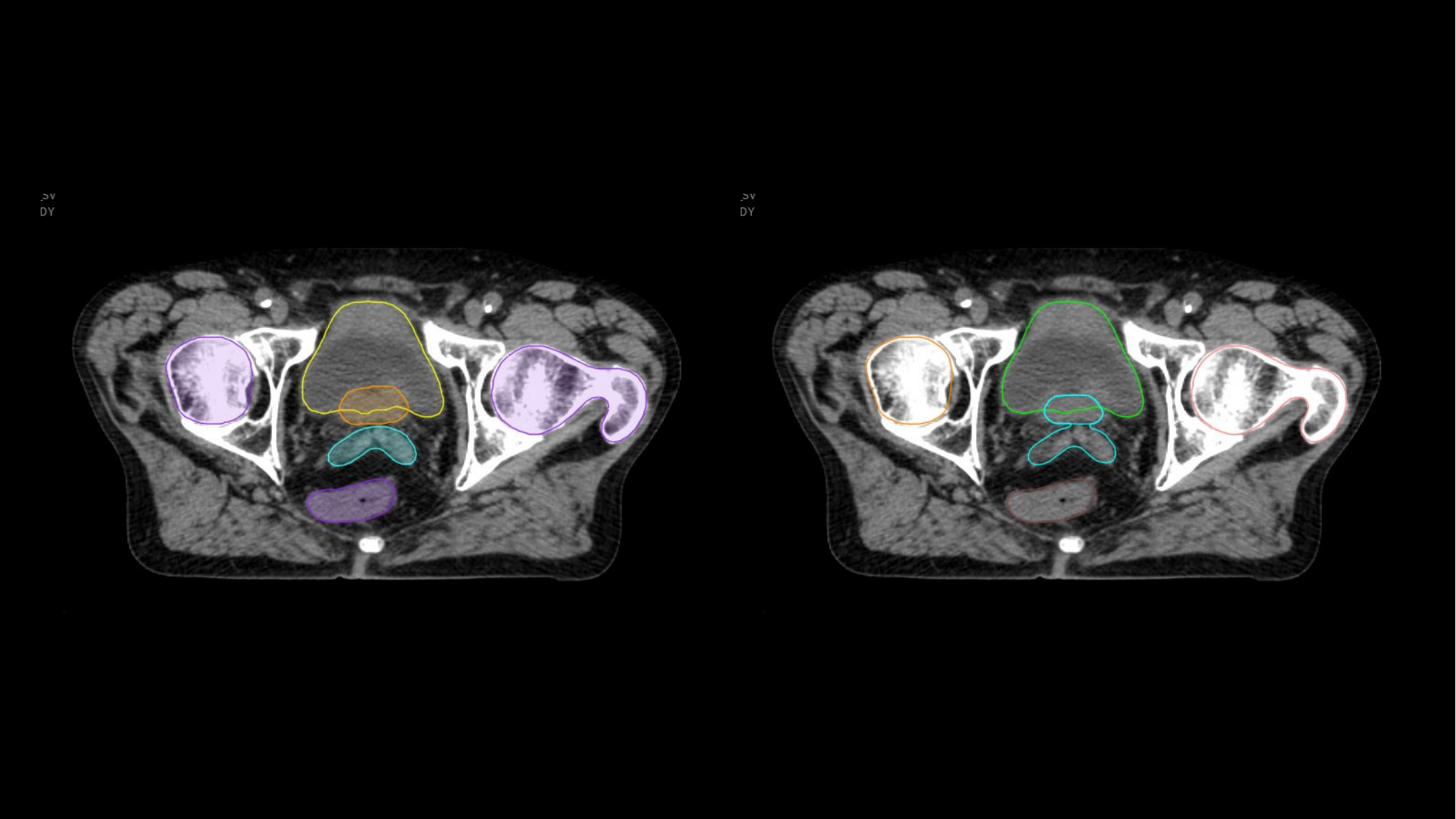

## Slide 55
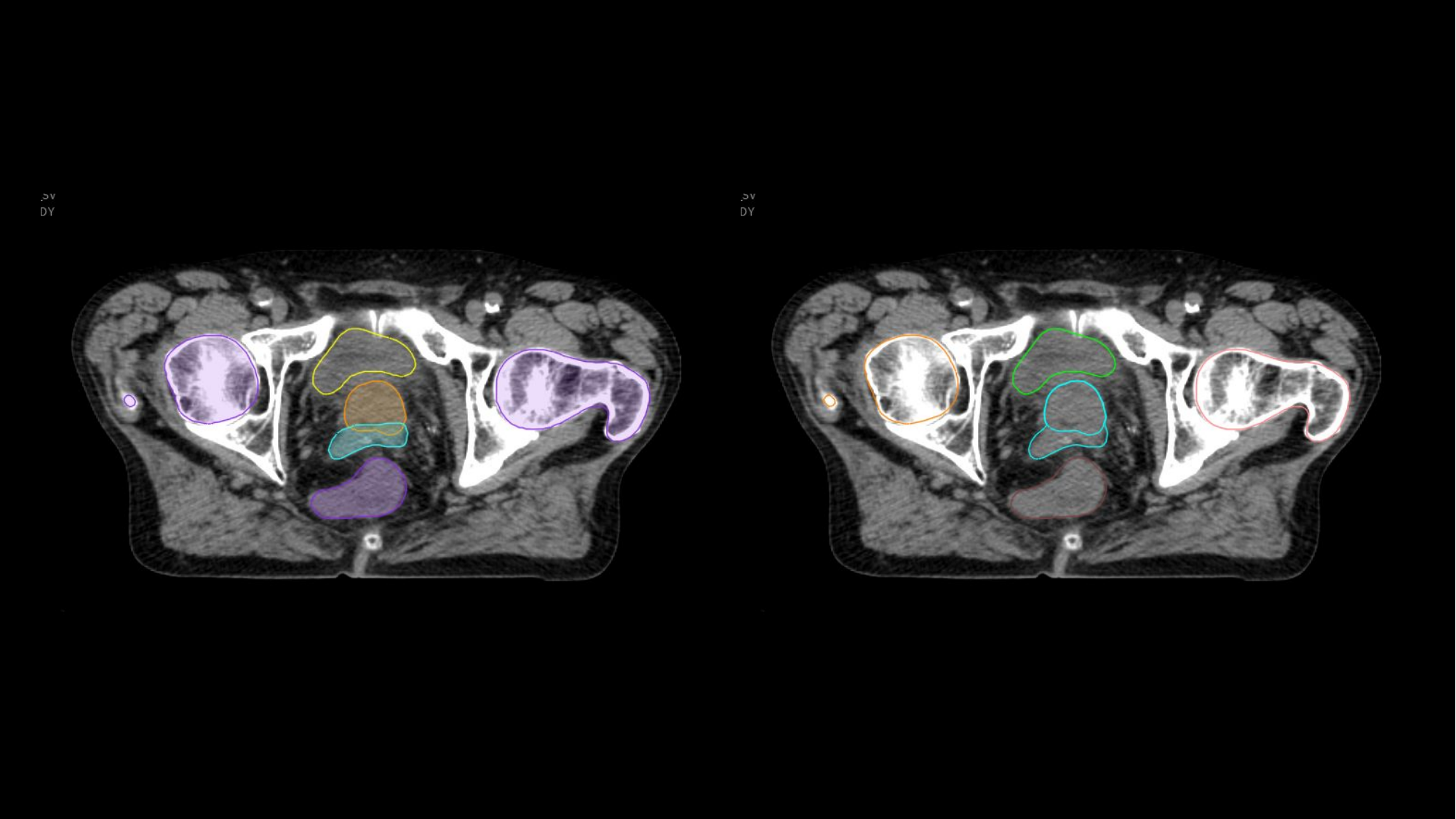

## Slide 56
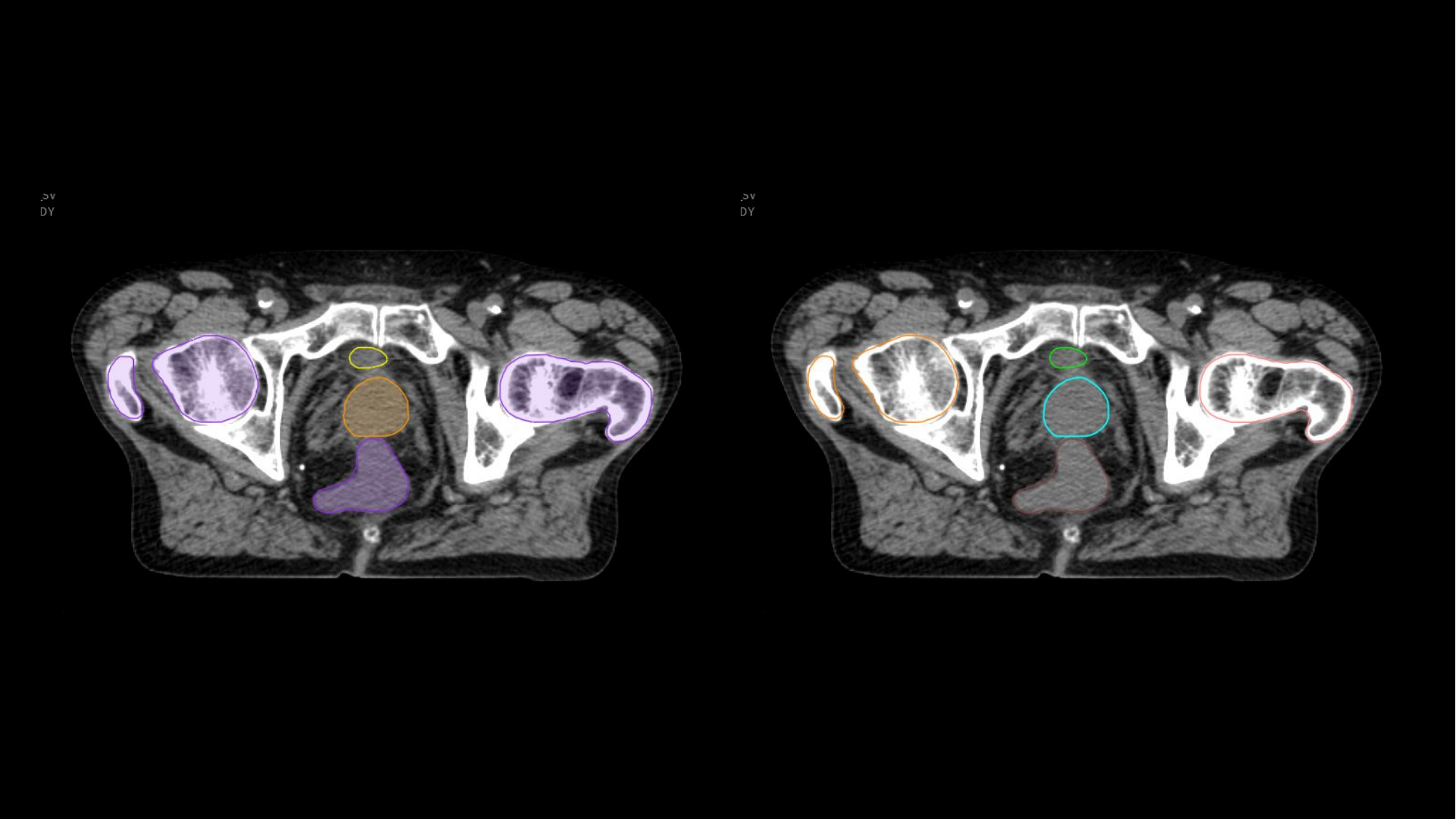

## Slide 57
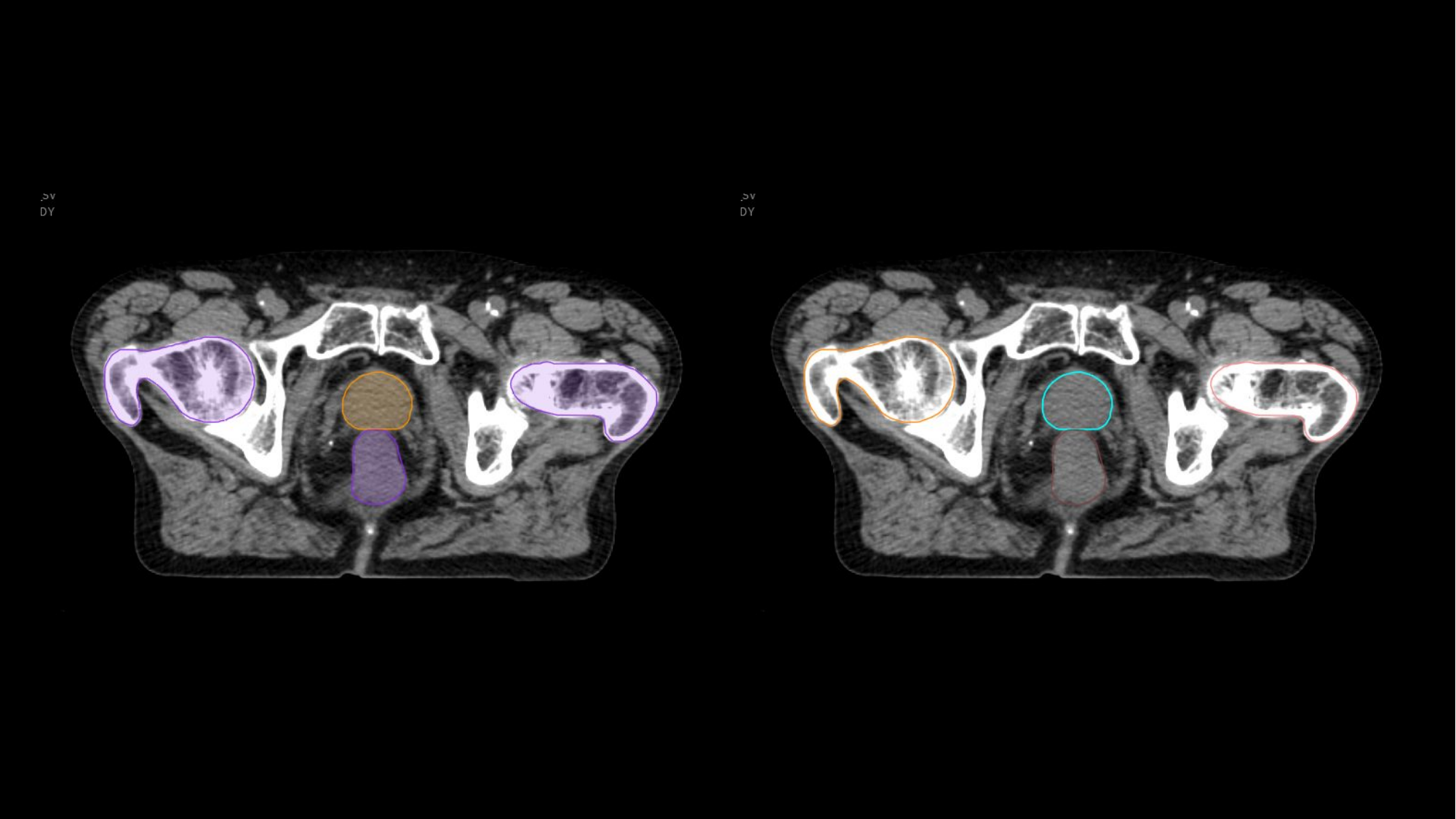

## Slide 58
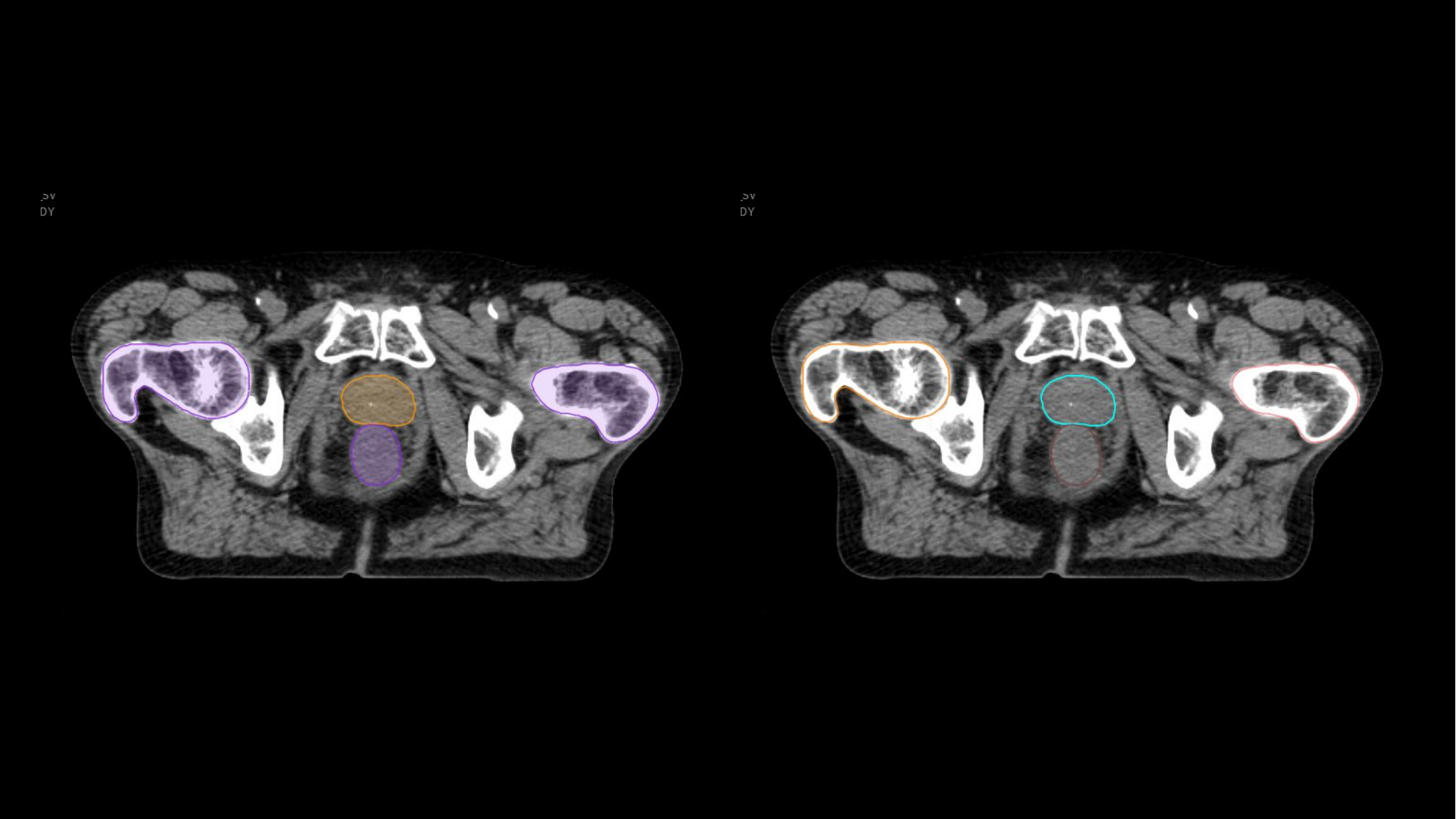

## Slide 59
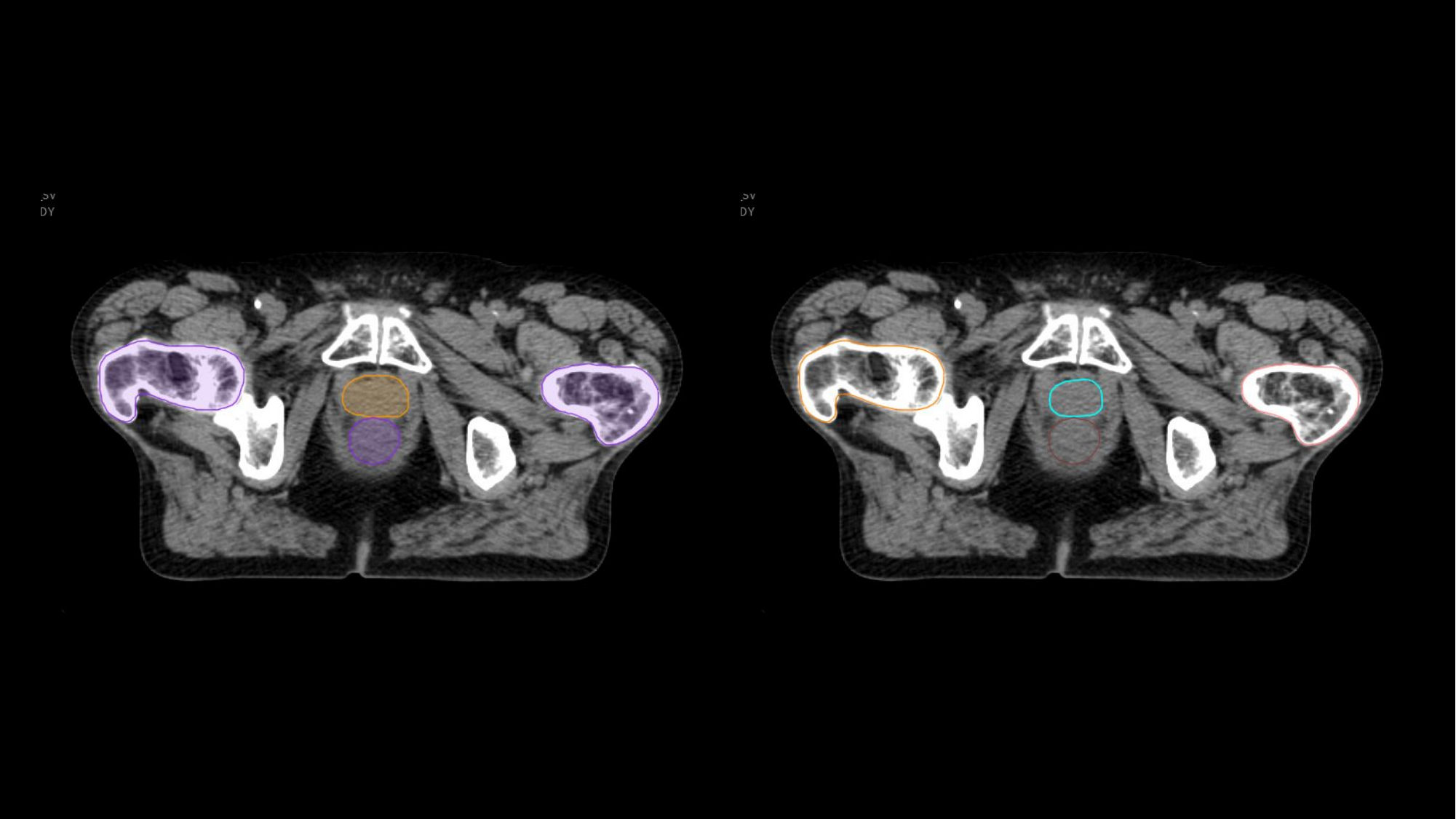

## Slide 60
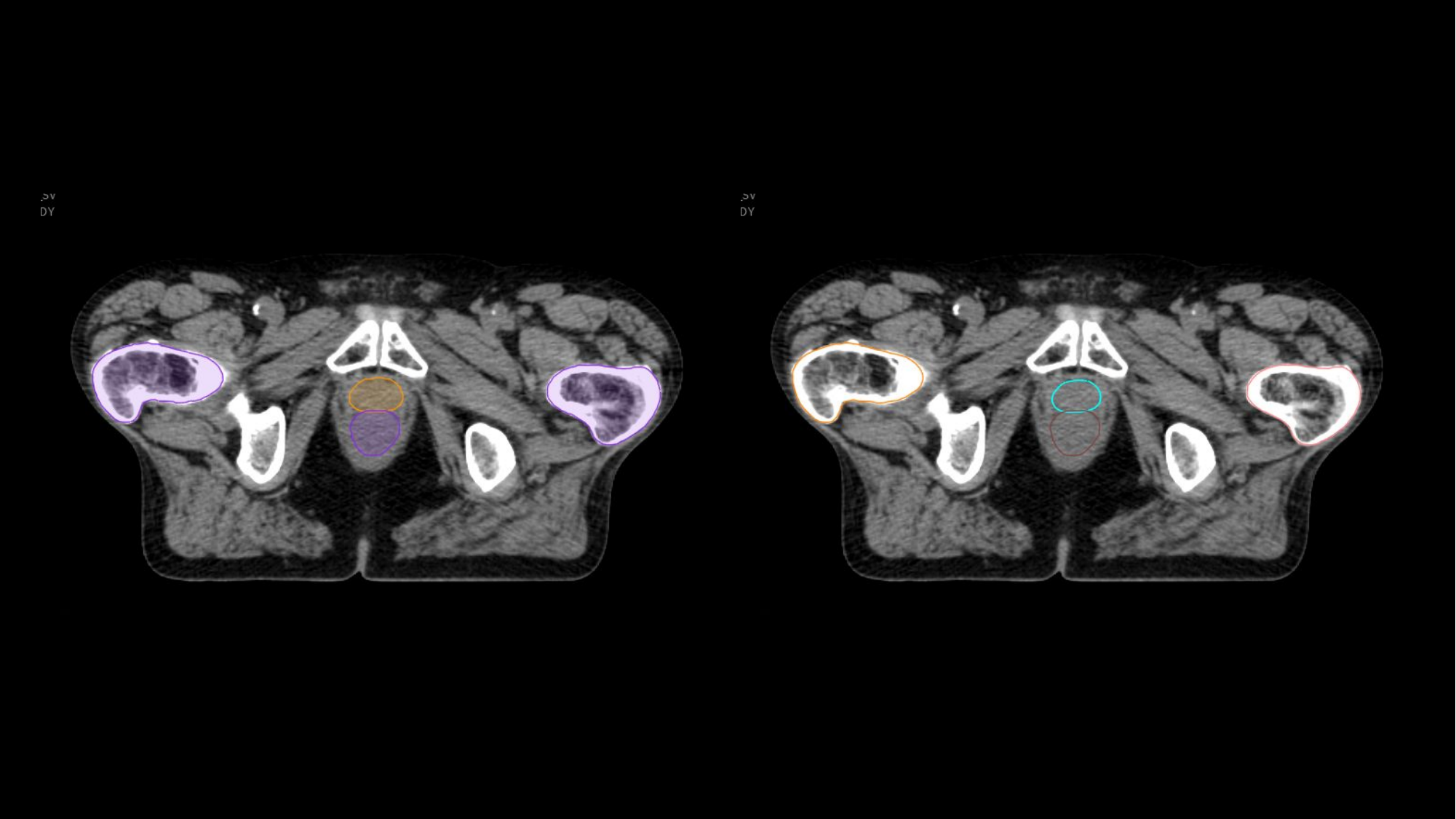

## Slide 61
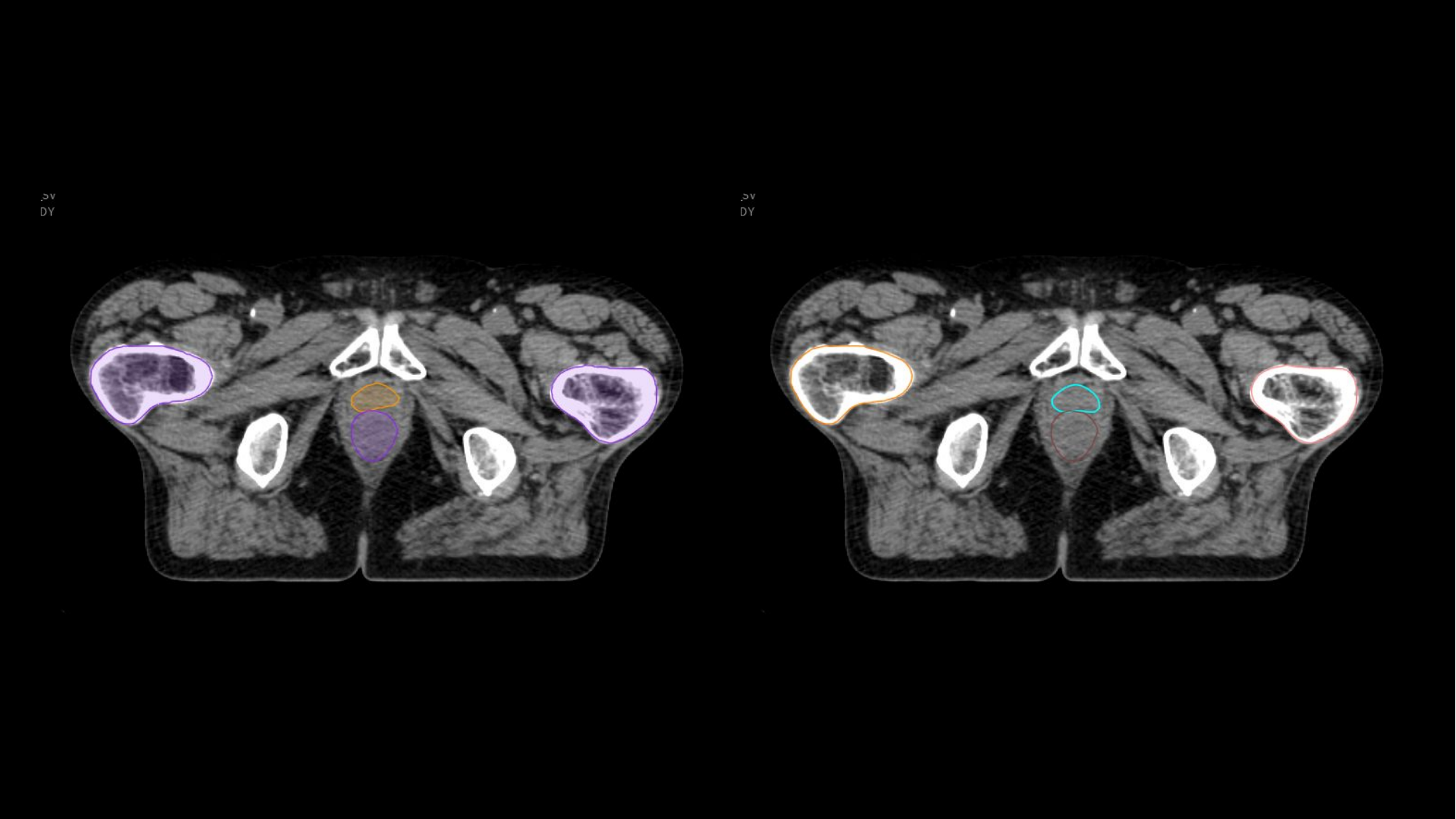

## Slide 62
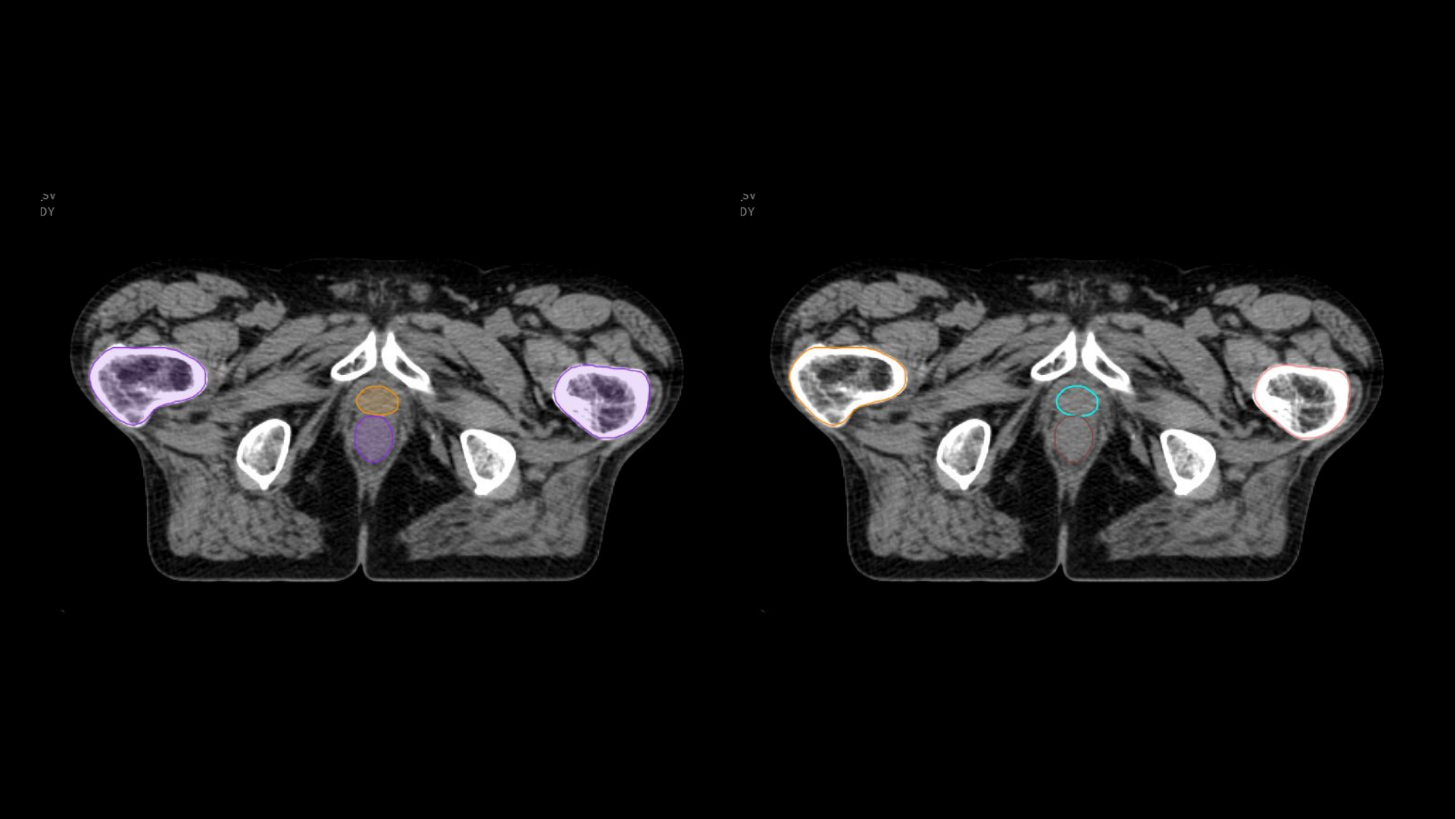

## Slide 63
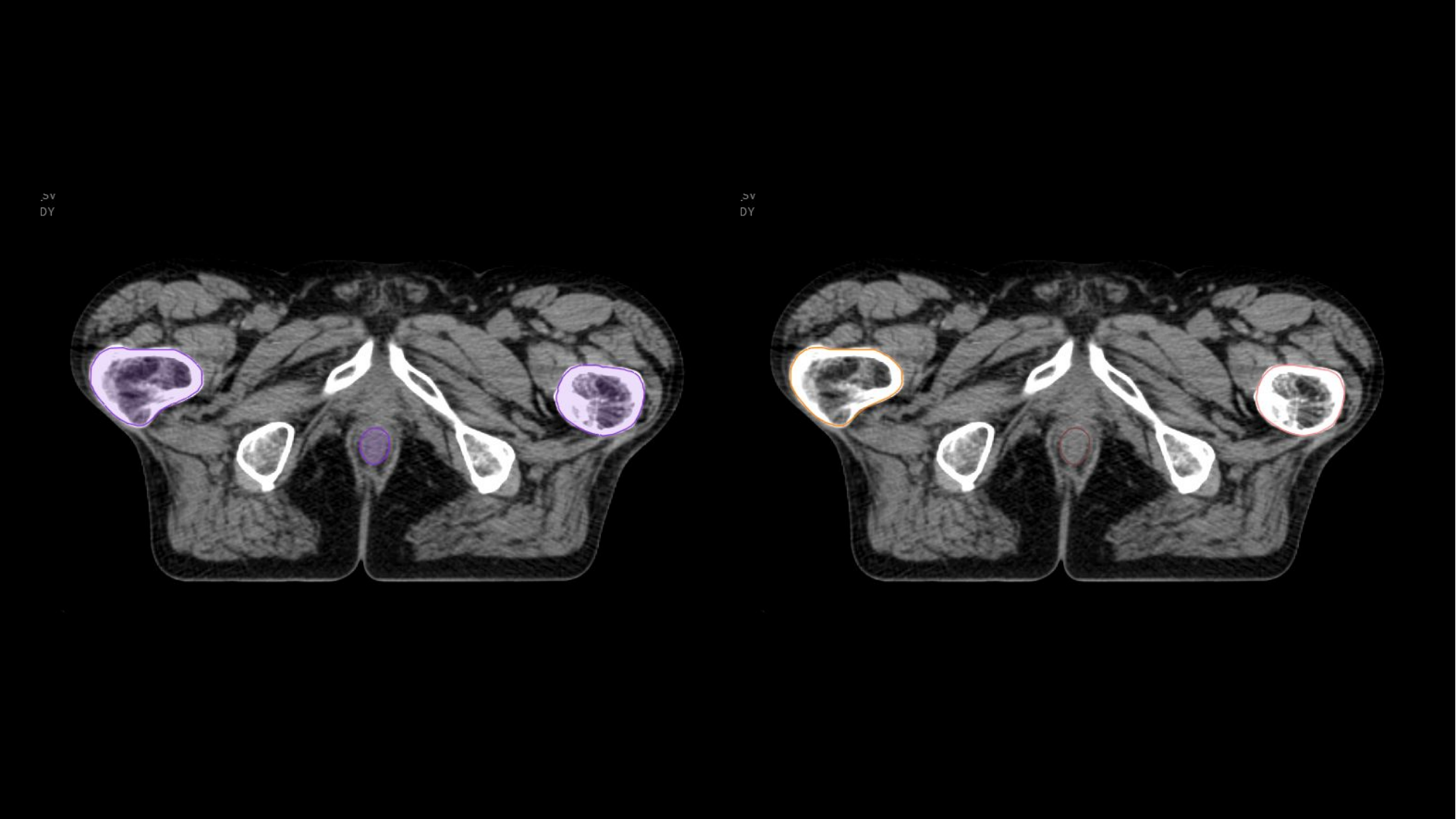

## Slide 64
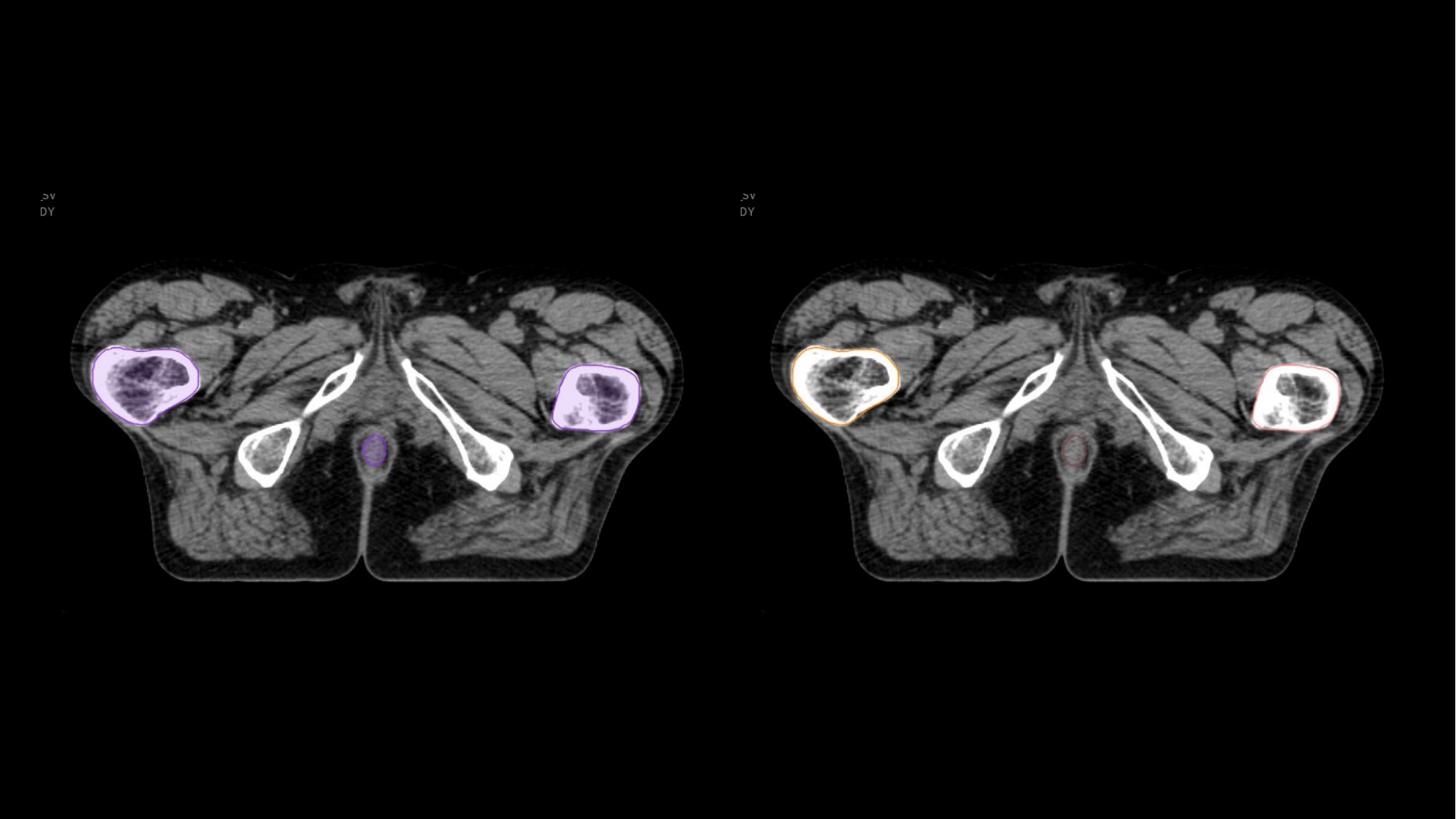

## Slide 65
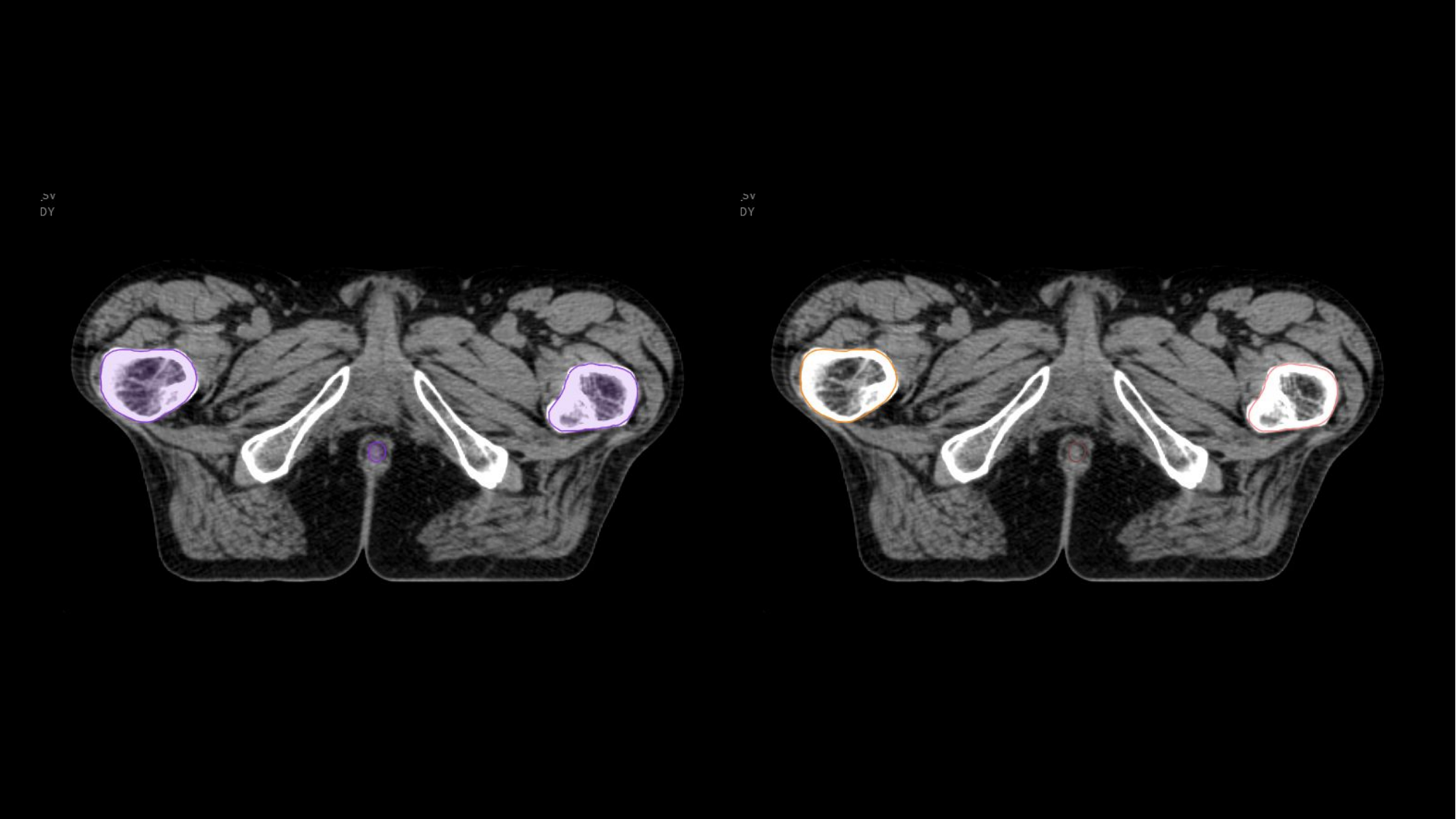

## Slide 66
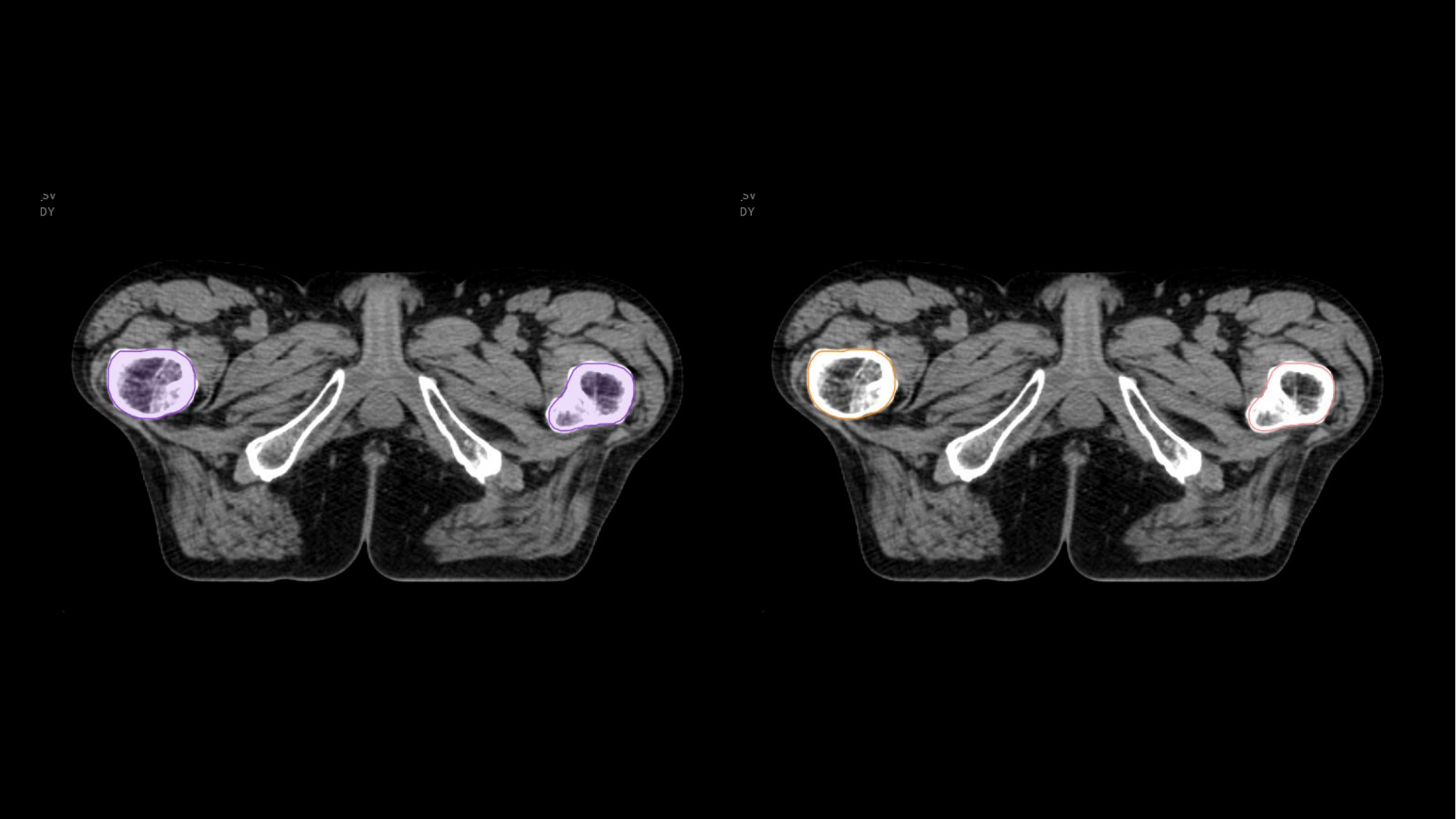

## Slide 67
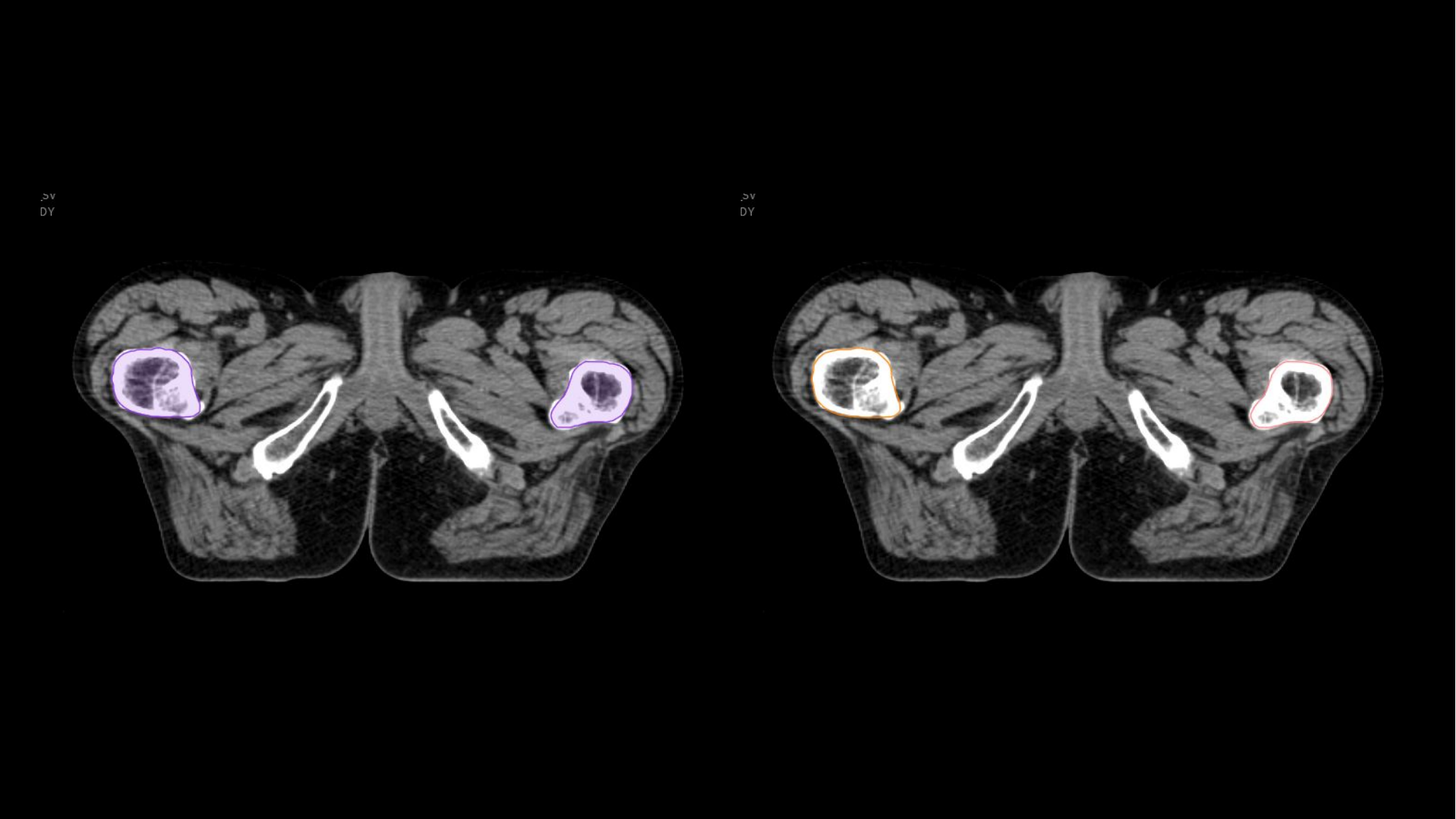

Supplement: Supplementary file 2 — Additional file 2. Additional plots correlating survey editing scores with Dice similariy coefficients and 95% Hausdorff distances. [file 13014_2021_1831_MOESM2_ESM.pptx]
