# Supplementary material for: Implementation of deep learning-based auto-segmentation for radiotherapy planning structures: a workflow study at two cancer centers
Source: Radiat Oncol. 2021 Jun 8;16:101. doi: 10.1186/s13014-021-01831-4 (PMC8186196; doi:10.1186/s13014-021-01831-4)
Supplement: Supplementary file 3 — Additional file 3. Examples cases with unedited and edited organs and risk and clinical target volume contours. [file 13014_2021_1831_MOESM3_ESM.docx]

Table S1. Summary of editing scores and comparison metrics for prostate organs at risk and clinical target volumes (CTV) in cases with rectal spacers. For editing scores: 1 = minimal editing required, 5 = significant editing required.

|  | **Editing Score** | | | **Number** | **95% Hausdorff distance (mm)** | | **Dice similarity coefficient** | |
| --- | --- | --- | --- | --- | --- | --- | --- | --- |
|  | **Number** | **Median (range)** | **Mean** |  | **Median (range)** | **Mean** | **Median (range)** | **Mean** |
| Bladder | 3 | 2 (1-2) | 1.7 | 5 | 0.26 (0.22-3.61) | 1.18 | 0.99 (0.95-1) | 1.0 |
| Left Femoral Head | 3 | 2 (1-3) | 2 | 5 | 0.21 (0.2-2.07) | 0.79 | 0.99 (0.97-1) | 1.0 |
| Right Femoral Head |  |  |  | 5 | 1.26 (0.2-8.88) | 2.55 | 0.99 (0.95-1) | 1.0 |
| Prostate | 1 | 2 (2-2) | 2 | 4 | 7.54 (6.23-9.31) | 7.65 | 0.8 (0.71-0.87) | 0.8 |
| Rectum | 3 | 3 (2-4) | 3 | 5 | 8.04 (2.64-12.14) | 8.00 | 0.84 (0.81-0.96) | 0.9 |

Table S2. Summary of editing scores for central nervous system, head and neck, and prostate organs at risk and clinical target volumes (CTV) by center. 1 = minimal editing required, 5 = significant editing required.

|  | **Center A** | | | **Center B** | | |
| --- | --- | --- | --- | --- | --- | --- |
|  | **Number** | **Median (range)** | **Mean** | **Number** | **Median (range)** | **Mean** |
| Brainstem | 28 | 1 (1-5) | 1.5 | 10 | 2 (1-3) | 1.9 |
| Globes | 58 | 1 (1-5) | 1.4 | 8 | 2 (1-5) | 2.5 |
| Optic Chiasm | 15 | 3 (1-5) | 3.2 | 5 | 4 (2-5) | 3.8 |
| Optic Nerve | 15 | 1 (1-2) | 1.2 | 7 | 2 (1-3) | 1.7 |
| Spinal Cord | 38 | 1 (1-4) | 1.2 | 8 | 2 (1-4) | 2.0 |
| Parotids | 19 | 1 (1-3) | 1.6 | 7 | 3 (1-5) | 3.0 |
| Submandibulars | 20 | 1 (1-3) | 1.5 | 0 | N/A | N/A |
| Mandible | 19 | 2 (1-4) | 2.3 | 7 | 2 (1-4) | 2.4 |
| Neck CTVs | 2 | 2 (1-3) | 2.0 | 0 | N/A | N/A |
| Bladder | 77 | 1 (1-3) | 1.4 | 9 | 2 (1-3) | 1.7 |
| Femoral Heads | 79 | 1 (1-3) | 1.6 | 10 | 2 (1-3) | 2.2 |
| Prostate | 9 | 3 (2-4) | 2.8 | 0 | N/A | N/A |
| Rectum | 74 | 1 (1-4) | 1.5 | 9 | 3 (2-4) | 2.7 |
| Seminal Vesicles | 10 | 2 (1-4) | 2.1 | 0 | N/A | N/A |

Table S3. Summary of comparison metrics from comparing unedited deep learning-based auto-segmented contours and final treatment approved contours for central nervous system, head and neck, and prostate organs at risk and clinical target volumes (CTV) by center.

|  | **Center A** | | | | | **Center B** | | | | |
| --- | --- | --- | --- | --- | --- | --- | --- | --- | --- | --- |
|  | **Number** | **95% Hausdorff distance (mm)** | | **Dice similarity coefficient** | | **Number** | **95% Hausdorff distance (mm)** | | **Dice similarity coefficient** | |
|  |  | **Median (range)** | **Mean** | **Median (range)** | **Mean** |  | **Median (range)** | **Mean** | **Median (range)** | **Mean** |
| Brainstem | 46 | 1.045 (0-7.76) | 1.80 | 0.98 (0.72-1) | 0.94 | 10 | 2.815 (0-3.67) | 2.20 | 0.93 (0.89-1) | 0.94 |
| Left Globe | 39 | 0.7 (0-2.81) | 0.90 | 0.98 (0.91-1) | 0.97 | 9 | 2.17 (0-3.27) | 1.76 | 0.96 (0.86-1) | 0.94 |
| Right Globe | 38 | 0.49 (0-3.5) | 0.89 | 0.98 (0.86-1) | 0.97 | 9 | 2.23 (0-2.93) | 1.61 | 0.94 (0.85-1) | 0.93 |
| Optic Chiasm | 20 | 6.32 (0-10.63) | 5.91 | 0.36 (0.15-1) | 0.48 | 10 | 3.61 (0-7.72) | 3.57 | 0.715 (0.39-1) | 0.69 |
| Left Optic Nerve | 22 | 1.445 (0-7.36) | 1.85 | 0.92 (0.61-1) | 0.86 | 10 | 1.81 (0-4.68) | 1.89 | 0.855 (0.62-1) | 0.87 |
| Right Optic Nerve | 23 | 0.75 (0-7.17) | 1.71 | 0.96 (0.54-1) | 0.87 | 10 | 1.56 (0-7.99) | 1.89 | 0.895 (0.66-1) | 0.88 |
| Mandible | 27 | 1.52 (0.2-2.98) | 1.45 | 0.95 (0.85-0.99) | 0.95 | 7 | 1.59 (0-2.13) | 1.54 | 0.96 (0.94-1) | 0.97 |
| Left Parotid | 28 | 2.45 (0.21-8.74) | 2.71 | 0.955 (0.78-0.99) | 0.94 | 7 | 3.6 (2.48-5.73) | 3.81 | 0.92 (0.87-0.96) | 0.92 |
| Right Parotid | 28 | 2.355 (0.22-7.64) | 2.58 | 0.945 (0.82-0.99) | 0.94 | 7 | 4.86 (1.82-7.34) | 4.48 | 0.95 (0.84-0.99) | 0.93 |
| Left Submandibular | 20 | 2.22 (0.26-7.56) | 2.51 | 0.96 (0.66-0.99) | 0.93 | 0 | N/A | | | |
| Right Submandibular | 23 | 2.34 (0.25-6.87) | 2.00 | 0.95 (0.78-0.99) | 0.94 | 0 | N/A | | | |
| Spinal Cord | 28 | 0.89 (0.23-3.81) | 1.29 | 0.96 (0.64-0.99) | 0.89 | 10 | 0.38 (0-1.65) | 0.66 | 0.98 (0.94-1) | 0.98 |
| Bladder | 60 | 0.635 (0-19.54) | 1.47 | 0.99 (0.93-1) | 0.99 | 11 | 0.7 (0.02-10.97) | 1.75 | 0.99 (0.92-1) | 0.99 |
| Left Femoral Head | 60 | 1.265 (0-7.24) | 1.48 | 0.99 (0.93-1) | 0.98 | 11 | 1.31 (0-5.02) | 1.49 | 0.98 (0.95-1) | 0.98 |
| Right Femoral Head | 60 | 1.26 (0-8.88) | 1.68 | 0.99 (0.93-1) | 0.98 | 11 | 1.73 (0.03-8.07) | 2.25 | 0.97 (0.94-1) | 0.97 |
| Prostate | 51 | 4.26 (0.2-50.15) | 6.29 | 0.9 (0.18-1) | 0.88 | 0 | N/A | | | |
| Rectum | 55 | 2.83 (0-17.3) | 4.54 | 0.96 (0.77-1) | 0.94 | 11 | 5.47 (2.64-11.27) | 5.87 | 0.93 (0.77-0.96) | 0.92 |
